# Photocatalytic Activation of Heterocyclic Iodonium Ylides for the Synthesis of Dihydrofuropyranones and Dihydrofuropyridones

**DOI:** 10.1021/acs.joc.5c01187

**Published:** 2025-09-11

**Authors:** Kelsey T. Sumter, Carly Slough, Hayley E. Johnson, Catherine S. Kesler, Mary Elisabeth Daub

**Affiliations:** Department of Chemistry, 3628 Furman University, 3300 Poinsett Highway, Greenville, South Carolina 29613, United States

## Abstract

β-Distabilized iodonium ylides undergo photocatalytic activation using neutral eosin Y and visible light to afford dihydrofuropyranones, dihydrofuropyridones, and dihydrofurans. Steady-state absorption spectroscopy and application of the method of continuous variations to complexation-induced chemical shifts provide evidence for the binary complexation of eosin Y and β-distabilized iodonium ylides. Preliminary mechanistic studies using transient absorption spectroscopy suggest monoanionic eosin Y as the active photocatalyst functioning via an oxidative quenching cycle. This simple approach to enhancing the reactivity of heterocyclic iodonium ylides expands the scope of their use as building blocks in the synthesis of heterocycles.

## Graphical abstract

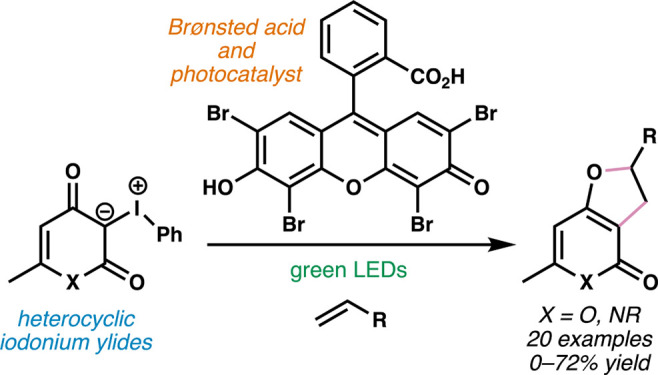

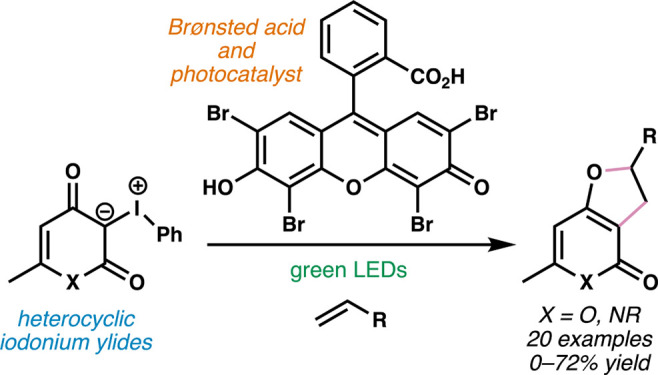

## Introduction

Furopyranone and furopyridone heterocycles comprise the core of several classes of natural products (Scheme 1a).^1–10^ Their structural complexity and diverse biological activities have inspired several syntheses of furopyranone and furopyridone natural products.^1,11–17^ Notably, an oxidative [3 + 2] cycloaddition approach to the furopyridones has never been reported, despite the strategic disconnection used in the synthesis of furopyranone natural products.^18–20^ The typical oxidative approaches, including ceric­(IV) ammonium nitrate,^21–28^ silver salts,^29^ and manganese­(III) acetate,^30–36^ that provide efficient access to furopyranones, furocoumarins, and furoquinolinones are often not amenable to the furopyridone system (Scheme 1b). Kobayashi and co-workers showed that ceric­(IV) ammonium nitrate failed to provide furopyridones, despite providing furopyranones in moderate to good yields.^24^ In an effort to develop a [3 + 2] cycloaddition amenable to both fused heterocycles, we identified an opportunity to explore the synthetic utility of iodonium ylides derived from 4-hydroxy-2-pyrones and 4-hydroxy-2-pyridones (e.g., **4** and **5**).^37^

**Scheme 1:**
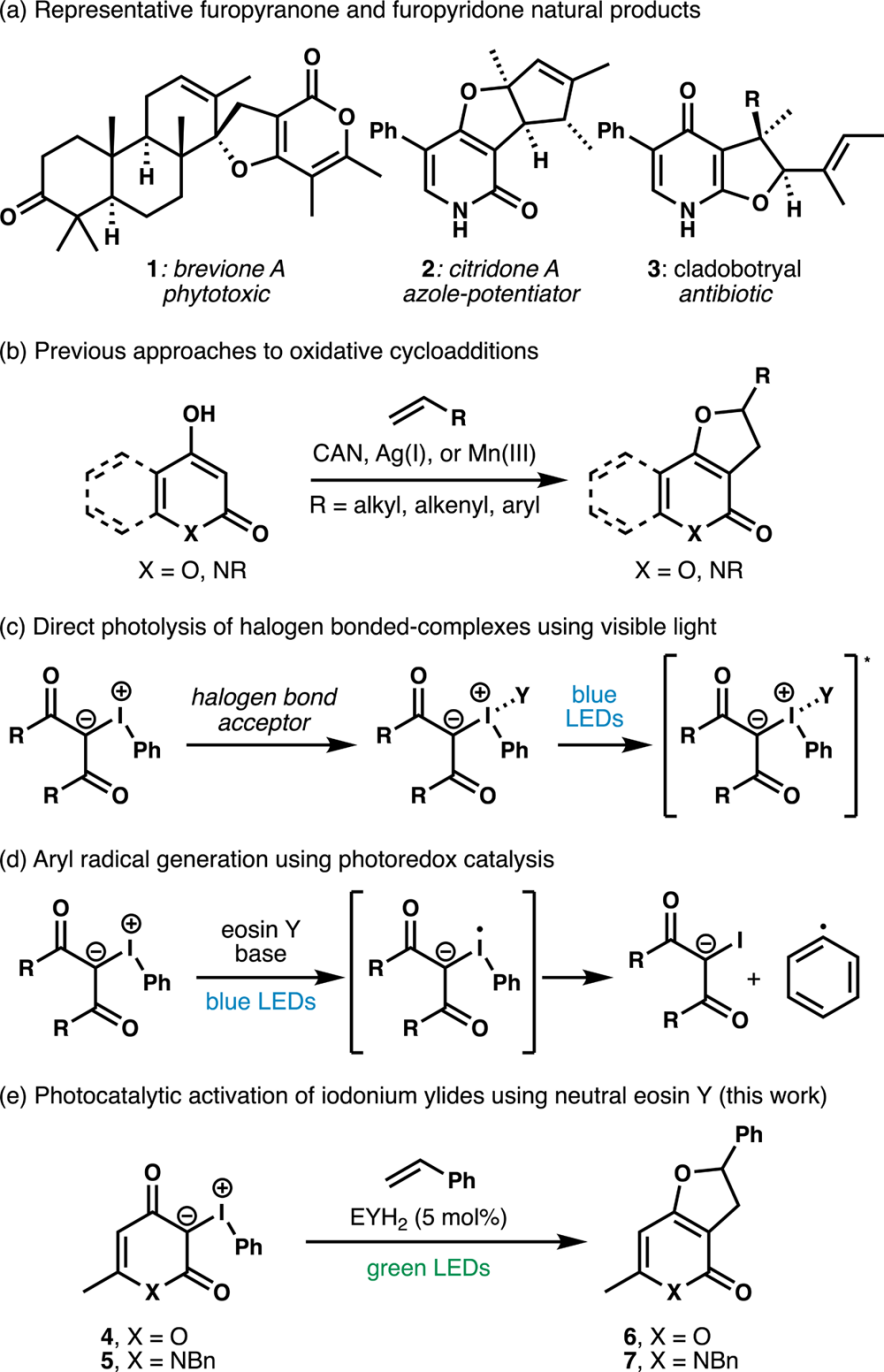
Representative Natural Products, Oxidative Approaches to the Furopyranone Core, and Photochemical Modes of Activating β-Distabilized Iodonium Ylides

Iodonium ylides derived from cyclic and acyclic 1,3-dicarbonyls are important building blocks in synthetic chemistry, most commonly as precursors to carbenes, given their similarities to diazo compounds.^38–51^ Several modes of activating β-distabilized iodonium ylides have been reported, including metal-catalyzed carbene formation,^39,52–57^ direct excitation with ultraviolet (UV) light,^58–60^ reaction with aryl-λ^3^-iodanes,^61,62^ Lewis acid catalysis,^63,64^ and Lewis base activation of σ-holes in iodonium ylides (i.e., halogen bonding).^65–67^ With growing interest in the use of hypervalent iodine reagents in visible light photocatalysis,^51,68–71^ two primary strategies for photocatalytic activation of iodonium ylides with visible light have emerged—direct irradiation of a halogen-bonded complex formed from an iodonium ylide and a halogen bond acceptor (Scheme 1c) and reduction of an iodonium ylide using photoredox catalysis (Scheme 1d).^72–78^ In 2019, Murphy and co-workers proposed a halogen-bonded complex formed from iodonium ylides and alkenes as an intermediate in a cyclopropanation reaction using blue light.^72^ Since then, the groups of Sen, Xuan, and Zhao have reported formation of EDA complexes between iodonium ylides and pyrroles, amines, and inorganic bases for C–H functionalization reactions using visible light.^73–75^ Work from the groups of Wu and He showed that iodonium ylides derived from dimedone have also served as a source of aryl radicals for C–H arylation reactions generated by reduction using visible light and organic photocatalysts, including eosin Y and 1,2,3,5-tetrakis­(carbazol-9-yl)-4,6-dicyanobenzene (4-CzIPN).^77,78^ Our investigations into the modes of photochemical activation of iodonium ylides led us to identify neutral eosin Y (EYH_2_) as a catalyst for [3 + 2] reactions of iodonium ylides and styrenes (Scheme 1e).

## Results and Discussion

Based on the reported reduction potentials of related iodonium ylides,^79^ we selected EYH_2_ (*E*_1/2_ = −1.15 V vs SCE) as a possible photocatalyst for the reduction of iodonium ylide **4**.^80^ The acidity of EYH_2_ along with the equilibrium between the quinonoid and lactone forms complicates the photophysical properties, although it is well-established that most photoredox reactions occur using the monoanionic (EYH^–^) or dianionic (EY^2–^) forms of eosin Y.^81–86^ The neutral form of eosin Y, however, has been shown to be an effective catalyst for hydrogen atom transfer (HAT).^87,88^ Furthermore, the predominant form of eosin Y in solution can change depending on the basicity of the solvent and solvent impurities.^83^ Therefore, we first measured the UV–Vis absorption of EYH_2_ in MeCN, which was consistent with a mixture of the neutral (λ_max_ = 473 nm in MeCN) and monoanionic (λ_max_ = 539 nm in MeCN) forms of eosin Y (see SI, Figure S2).^89^ In contrast, the 1:1 mixture of EYH_2_ and iodonium ylide **4** (λ_max_ = 539 nm in MeCN) exhibited a maximum absorption peak consistent with the characteristic absorption peak of monoanionic eosin Y, suggesting the formation of a complex between iodonium ylide **4** and eosin Y (see SI, Figure S2). Using the method of continuous variations to analyze complexation-induced chemical shifts in the ^1^H NMR spectrum revealed a 1:1 stoichiometry of the eosin Y–ylide complex (see SI, Figure S5).^90–92^

Irradiation of a mixture of iodonium ylide **4**, styrene, and EYH_2_ with 525 nm light provided furopyranone **6** in 65% yield (Table 1, entry 1). Using 467 nm LEDs as the light source decreased the yield of furopyranone **6** to 53% (entry 2). The acidity of eosin Y affected the efficiency of the reaction, as using the eosin Y disodium salt (EYNa_2_) led to **6** in only 31% yield (entry 3). Addition of 10 mol % of *p*-toluenesulfonic acid, however, improved the yield using EYNa_2_ as the photocatalyst (entry 4). We evaluated a transition metal photocatalyst with a similar excited-state reduction potential to eosin Y ([Ir­(Fppy)_2_(dtbbpy)]­(PF_6_), *E*_1/2_ = −1.04 V vs SCE)^93^ using 467 nm LEDs and observed furopyranone **6** in 42% yield (entry 5). A control reaction using 467 nm LEDs in the absence of photocatalyst (entry 6) revealed that furopyranone **6** can be formed via direct irradiation with blue light, albeit in lower yield.^72^

**Table 1:**
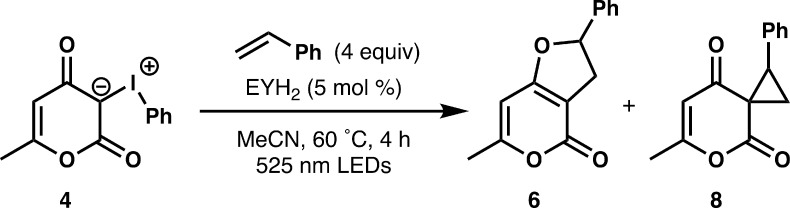
Optimization Studies of the [3 + 2] Photocycloaddition of Iodonium Ylides

entry	variation from standard conditions	yield **6** (%)^a^	yield **8** (%)^a^
1	none	65	0
2	467 nm LEDs *instead of* 525 nm LEDs	53	0
3	EYNa_2_ *instead of* EYH_2_	31	0
4	5 mol % EYNa_2_, 10 mol % *p*TsOH *instead of* EYH_2_	51	0
5	1 mol % [Ir(Fppy)_2_(dtbbpy)][(PF_6_)], 467 nm LEDs *instead of* EYH_2_, 525 nm LEDs	42	0
6	no EYH_2_, 467 nm LEDs *instead of* 525 nm LEDs	23	0
7	2 mol % EYH_2_	40	3
8	7 mol % EYH_2_	62	0
9	25 °C	11	16
10	45 °C	46	6
11	75 °C	57	0
12	irradiation for 30 min	17	21
13	acetone	57	0
14	DMF	39	0
15	DMSO	55	0
16	PhMe	0	0
17	MeOH	0^b^	0
18	no light	0	0
19	no EYH_2_	0	0

a Yield determined by ^1^H NMR analysis using phenanthrene as an internal standard and are the averaged results of two experiments. Reactions conducted on 0.15 mmol scale with respect to **4**.

b 8% of benzylic methoxylation product formed.

Modification of the catalyst loading led to a lower yield of **6** (entries 7 and 8), and examination of reaction temperature showed similarly decreased reaction efficiencies (entries 9–11). Notably, we observed cyclopropane **8** at 25 and 45 °C (entries 9 and 10), which suggested **8** as an intermediate in the reaction. To probe this further, we performed the reaction under the standard conditions with an irradiation time of only 30 min and observed both furopyranone **6** and cyclopropane **8** (entry 12). At longer reaction times, cyclopropane **8** rearranges to furopyranone **6** under the reaction conditions, a finding consistent with related work by Hadjiarapoglou and Murphy.^55,72^ Since the basicity of the solvent is known to impact the acid–base equilibria of the different forms of eosin Y, we evaluated several polar aprotic solvents of varying basicity (entries 13–15).^94^ The yields of furopyranone **6** were generally lower with acetone, DMF, and DMSO as the reaction solvent, which are more Lewis basic than acetonitrile.^95^ Both toluene and methanol proved to be poor solvent choices as furopyranone **6** was not observed in either case (entries 16 and 17). Control reactions revealed that both light and photocatalyst were required (entries 18 and 19), although a small quantity of the 3-iodo-4-phenoxy-2-pyrone resulting from the thermal rearrangement of **4** was observed in both cases.

Initial evaluation of the scope with respect to the styrene revealed competent reaction partners as well as limitations of the [3 + 2] photocycloaddition of iodonium ylides (Figure 1). Under the standard reaction conditions (0.6 mmol), we isolated furopyranone **6** in 49% yield, although this decreased to 41% on a larger scale (1 mmol). A variety of electron-rich and electron-deficient styrenes (**9**–**15**) provided the dihydrofuropyranone products in moderate yields, with electron-rich substituents generally providing higher yields. While 4-methoxystyrene led to low yields of the product owing to styrene decomposition under the reaction conditions, modulating the reactivity with a less electron-donating group (−OAc) restored the efficiency of the reaction (compare **13** and **14**). Furopyranones bearing a boronate ester (**15**) present a desirable target owing to the presence of a functional handle, but the reaction conditions have low compatibility with the boronate ester group. Substitution at the 2-position (**16**) and the β-position (**18**) of the styrene was well tolerated, although reaction yields were lower using α-methylstyrene as a substrate (**17**). Using cinnamyl alcohol provided furopyranone **19** in low yield along with cinnamaldehyde, indicating that alcohols are not compatible with the reaction conditions owing to competitive oxidation. Unfortunately, protecting the alcohol as acetate did not improve the yield of furopyranone **20**. For all 1,2-disubstituted styrenes, we observed exclusive formation of the *trans*-diastereomer, which is consistent with related oxidative reactions of 4-hydroxycoumarin and 1,2-disubstituted alkenes.^21^

**Figure 1:**
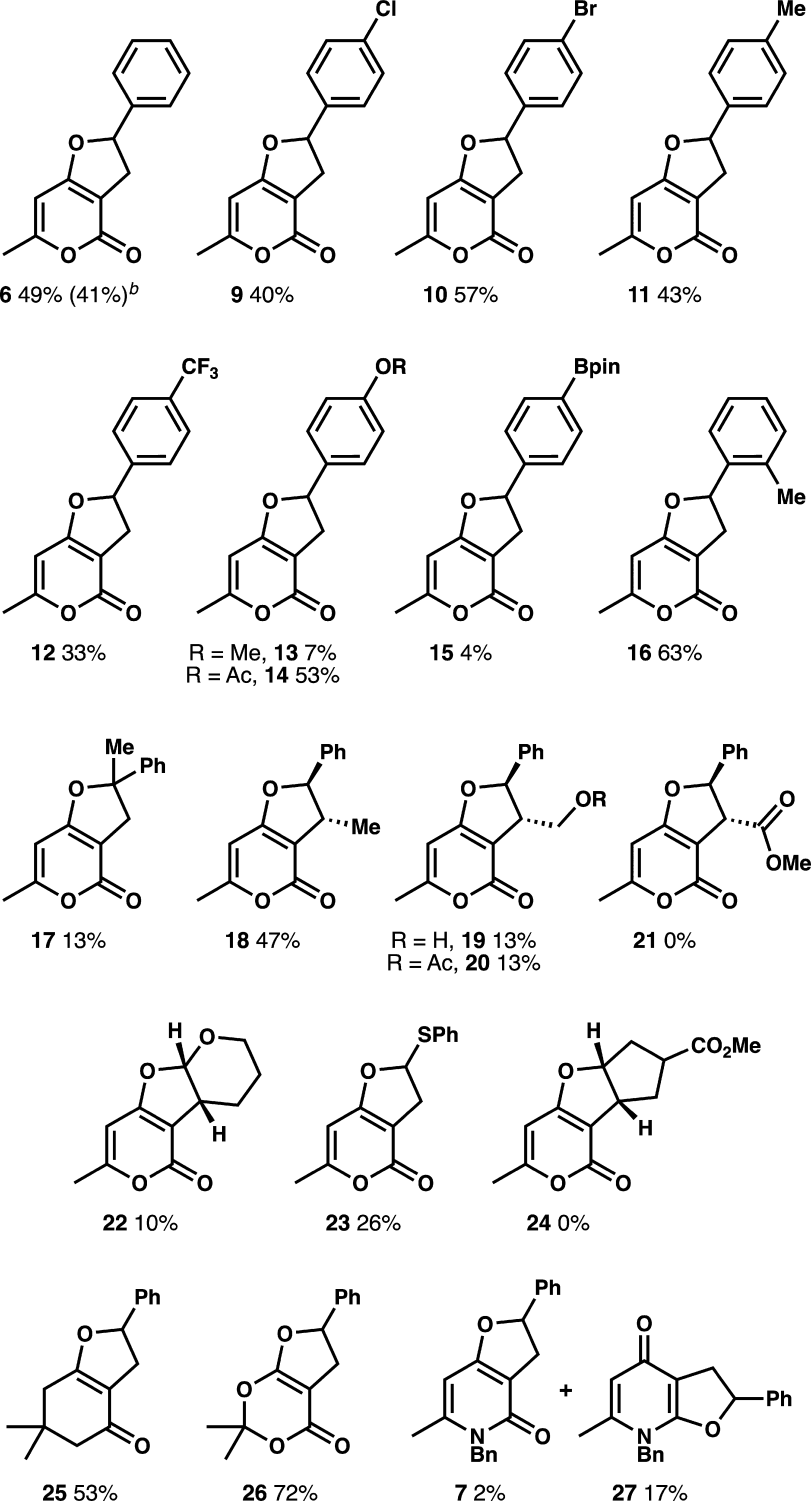
Scope studies for the [3 + 2] photocycloaddition of iodonium ylides.^*a a*^All reactions were conducted using 0.6 mmol iodonium ylide, 5 mol % EYH_2_, 4 equiv alkene, 6 mL MeCN, and irradiating for 4 h at 60 °C with two 525 nm Kessil lamps unless otherwise noted. ^*b*^Reaction was conducted on a 1 mmol scale.

We evaluated additional alkenes as reaction partners, including electron-deficient, electron-rich, and unactivated alkenes. Both methyl cinnamate and a substituted cyclopentene failed to provide cycloadducts (**21** and **24**). While electron-rich alkenes 3,4-dihydro-2*H*-pyran and phenyl vinyl sulfide were effective substrates, affording furopyranones **22** and **23**, the yields were low compared to those of styrenes. We conducted the [3 + 2] photocycloaddition using three other cyclic β-distabilized iodonium ylides. The iodonium ylide derived from dimedone was comparable to the reaction of heterocyclic iodonium ylide **4**, giving dihydrofuran **25** in 53%. The iodonium ylide derived from Meldrum’s acid, however, worked quite well compared to all other substrates evaluated, providing **26** in good yield. Lastly, extension of this method to an iodonium ylide derived from 4-hydroxy-2-pyridone (**5**) revealed furo­[2,3-*b*]­pyridine-4­(2*H*)-one **27** as the major product, albeit in low yield, along with a small quantity of furo­[3,2-*c*]­pyridine-4­(2*H*)-one **7**.

To provide insight into the reaction mechanism, we performed transient absorption spectroscopy on EYH_2_ (see SI, Figure S6) and a 1:1 mixture of EYH_2_ and iodonium ylide **4** (Figure 2).^96^ The transient absorption spectra of EYH_2_ in nondegassed MeCN exhibit triplet absorption peaks at 335 and 560–780 nm, which is consistent with previous reports for the neutral form of eosin Y.^88^ In contrast, the transient absorption spectra of the 1:1 mixture of EYH_2_ and iodonium ylide **4** show a ground state bleach (λ_GS_ = 540 nm), characteristic of the monoanionic form of eosin Y,^97^ and singlet (λ_S_ = 435 nm) and triplet (λ_S_ = 580 nm) absorption peaks consistent with previous reports for anionic forms of eosin Y.^97–101^ As the excited triplet monoanionic eosin Y decays (τ_T_ = 260 ns), an absorption peak at 460 nm rises with a time constant of 270 ns, corresponding to semioxidized eosin Y (EYH^•^).^97–100^ Overall, these results suggest that upon addition of iodonium ylide **4**, monoanionic eosin Y is formed, and the excited triplet monoanionic eosin Y is oxidatively quenched by ylide **4**.

**Figure 2:**
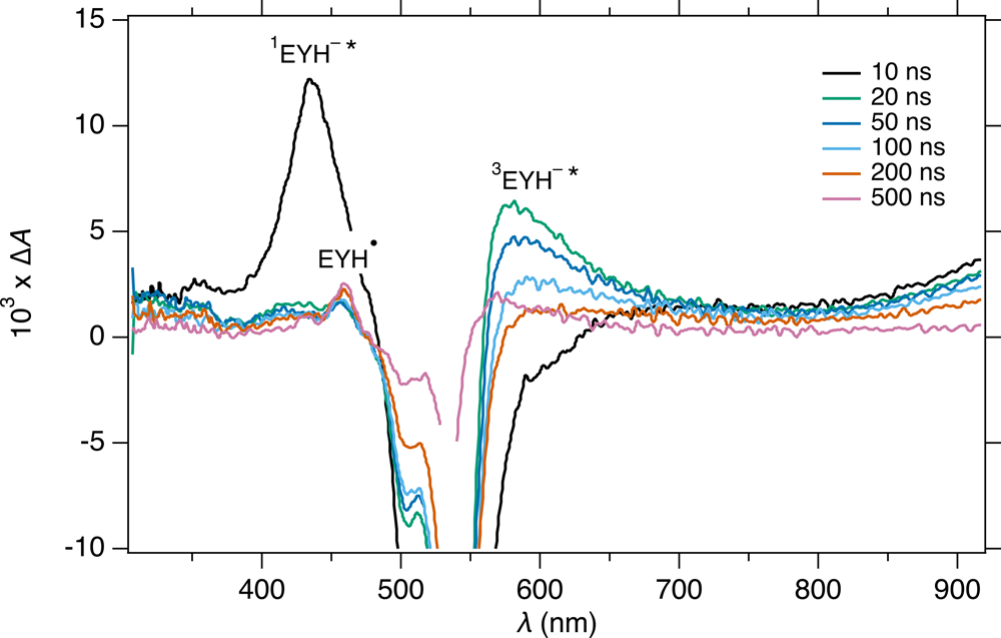
Transient absorption spectra of EYH_2_ (1 mM) and iodonium ylide **4** (1 mM) in nondegassed MeCN at different times after excitation (excitation wavelength 532 nm).

Additionally, radical trapping experiments with TEMPO (2,2,6,6-tetramethylpiperidine 1-oxyl) further supported a radical pathway (Scheme 2a). Upon addition of 2 equiv of TEMPO as a radical scavenger, we observed only TEMPO adduct **29** and 3-iodo-4-phenoxy-2-pyrone (**30**) in the crude ^1^H NMR spectrum, although TEMPO adduct **28** was detected using ESI-MS. Formation of adduct **29** suggests either radical addition of the reduced iodonium ylide to styrene or generation of an aryl radical following reduction of iodonium ylide **4**, as in related reactions of iodonium ylides.^77^ However, fragmentation of the reduced iodonium ylide is structure dependent. Iodonium ylides related to **4** (derived from 4-hydroxy-2-coumarins) are reported to liberate iodobenzene upon electrochemical reduction, whereas the iodonium ylide derived from dimedone primarily affords 2-iododimedone.^79^ Observation of dihydrofuran **25** as the only product of the [3 + 2] reaction using the iodonium ylide derived from dimedone leads us to favor the radical addition pathway over fragmentation.

**Scheme 2:**
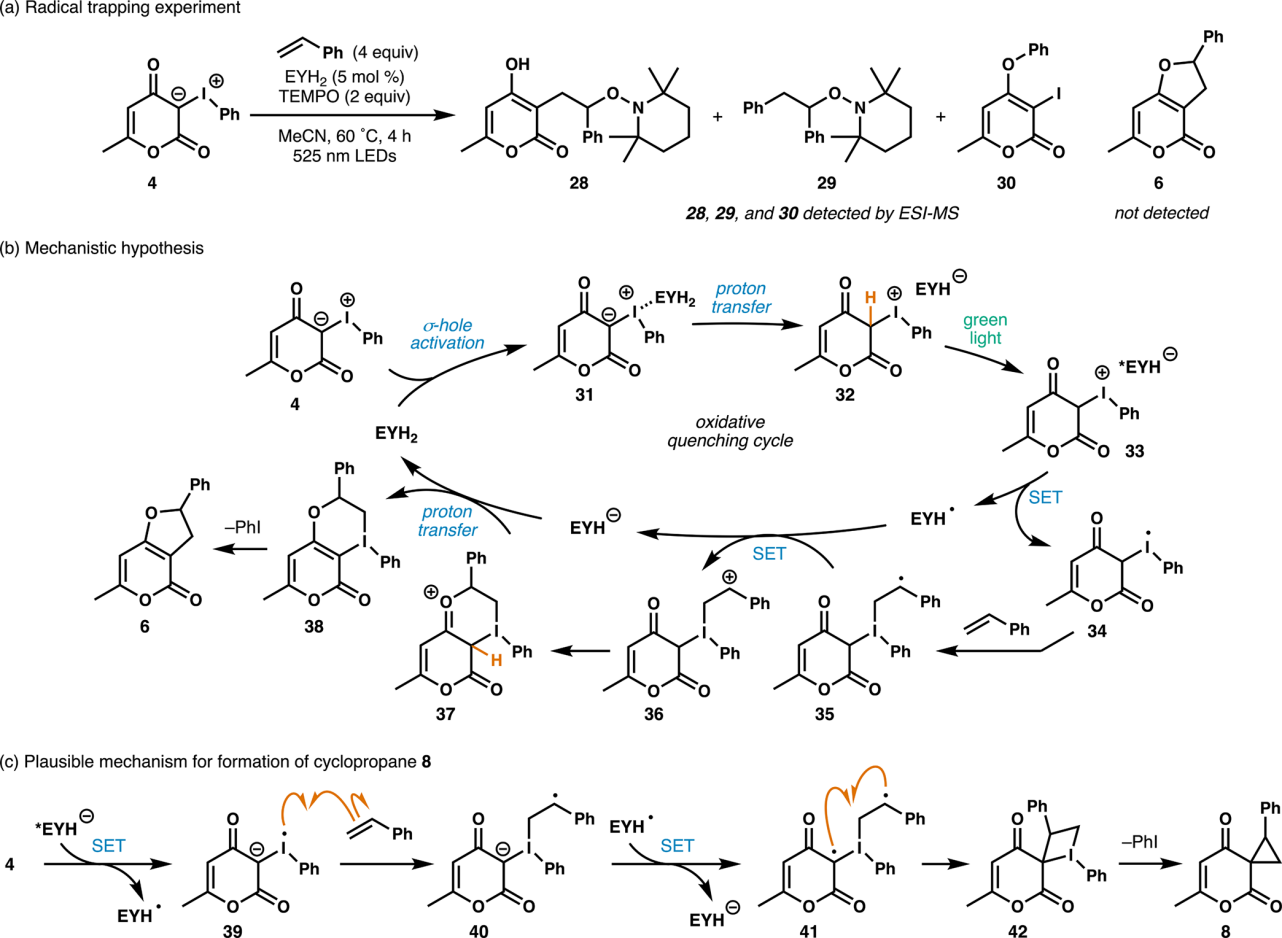
Mechanistic Proposal for the [3 + 2] Photocycloaddition of Iodonium Ylide **4** and Supporting Studies

Our current mechanistic proposal begins with σ-hole activation of iodonium ylide **4** by neutral eosin Y, forming halogen-bonded complex **31** (Scheme 2b). Subsequent proton transfer generates monoanionic eosin Y as part of acid–base complex **32**. While iodonium ylides are only weakly basic, Murphy and co-workers proposed that formation of a halogen bond between the σ-hole of an iodonium ylide and a weak acid enhances their acidity.^102^ After excitation with 525 nm light, excited-state monoanionic eosin Y donates an electron to the iodine center to generate radical **34**. From here, direct addition of radical **34** to styrene provides benzylic radical **35**. Oxidation of benzylic radical **35** by EYH^•^, nucleophilic capture of the carbocation, and a proton transfer gives iodinane **38** while regenerating neutral eosin Y. Finally, loss of iodobenzene affords furopyranone **6**.

Exclusive formation of the furo­[3,2-*c*]­pyran-4­(2*H*)-one ring system, arising from nucleophilic capture of carbocation **36** by the C4 carbonyl, is consistent with previous work on oxidative reactions of 4-hydroxy-2-pyrones.^24^ In contrast, upon generation of carbocation **36** using iodonium ylide **5** as the substrate, the C2 carbonyl outcompetes the C4 carbonyl for carbocation capture, resulting in furo­[2,3-*b*]­pyridine-4­(2*H*)-one **27** as the major product. Related work on oxidative cycloaddition of 4-hydroxy-2-quinolones with styrenes reports similar regioselectivity.^30^ Alkene substrates containing Lewis basic (*i.e*., **19**, **20**, **22**, and **23**) and acidic (*i.e*., **15**) sites generally provided lower yields of the corresponding furopyranone products, suggesting the proposed mode of complexation may be sensitive to the presence of other halogen bond acceptors and Lewis acids.

An alternate pathway that accounts for the observation of cyclopropane **8** at lower temperatures and shorter reaction times involves direct reduction of ylide **4** by excited triplet eosin Y to give radical **39** (Scheme 2c). After addition of the radical to styrene, oxidation of **40** by EYH^•^ generates biradical **41**.^62^ Finally, radical recombination and extrusion of iodobenzene lead to cyclopropane **8**, which subsequently rearranges to furopyranone **6** under the reaction conditions. Further studies to distinguish between these pathways are ongoing in our group.

## Conclusions

We have discovered a novel method for activating heterocyclic iodonium ylides using eosin Y as a Brønsted acid and a visible light photocatalyst. This work represents a fundamental step in exploring the reactivity of heterocyclic iodonium ylides and constitutes the first example of using visible light photocatalysis to initiate their decomposition. While the yields are generally low to moderate, thereby limiting the synthetic utility of the cycloaddition reaction, the starting materials and photocatalyst are readily accessible. Future work in our laboratory will explore additional mechanistic studies and the generality of using eosin Y to activate iodonium ylides.

## Supplementary Material



## Data Availability

The data underlying this study are available in the published article and its Supporting Information.

## Notes

Experimental procedures; characterization data; NMR spectra for all new compounds; job plot of complexation-induced chemical shifts, and steady-state and transient absorption spectra (PDF)
