# Supplementary material for: Photocatalytic Activation of Heterocyclic Iodonium Ylides for the Synthesis of Dihydrofuropyranones and Dihydrofuropyridones
Source: J Org Chem. 2025 Sep 11;90(38):13488–95. doi: 10.1021/acs.joc.5c01187 (PMC12481581; doi:10.1021/acs.joc.5c01187)

Photocatalytic Activation of Heterocyclic Iodonium Ylides for the Synthesis of Dihydrofuropyranones and Dihydrofuropyridones

Kelsey T. Sumter, Carly Slough, Hayley E. Johnson, Catherine S. Kesler, and Mary Elisabeth Daub*

Department of Chemistry, Furman University, 3300 Poinsett Highway, Greenville, South Carolina 29613, United States

marybeth.daub@furman.edu

Supporting Information

I. Table of Contents

I. Table of Contents…… S1

II. General Experimental Methods…… S2

III. Experimental Procedures and Characterization Data…… S4

IV. Radical Trapping Experiment…… S21

V. Steady-State UV–Vis Absorption Spectra …… S22

VI. Analysis of Complexation-Induced Chemical Shifts using MCV…… S23

VII. Transient Absorption Spectra…… S25

VIII. References…… S27

IX. NMR Spectra…… S28

II. General Experimental Methods

All reactions were performed under an inert atmosphere of nitrogen using oven-dried or flame-dried glassware and Teflon® coated stir bars. Commercial reagents were used as received unless noted otherwise. Anhydrous solvents (DMSO, CH_2Cl_2, acetone, DMF, PhMe, and MeOH) were purchased from commercial sources and used as received. Anhydrous acetonitrile (99.9%, extra dry, AcroSeal™) was purchased from Fisher Scientific (Catalog# AC610961000) and used for all experiments unless otherwise noted. All photochemical reactions were performed using PR160 Kessil LED lamps (467 or 525 nm). Reactions were monitored by thin-layer chromatography (TLC) performed on Supelco glass silica gel 60 TLC plates with 254 nm fluorescent indicator using UV light as a visualizing agent and ceric ammonium molybdate stain paired with heat as developing agent. Purification was performed via automated column chromatography using Sfär silica gel columns and a Biotage Isolera One Flash Chromatography system. NMR spectroscopy was recorded using a JEOL JHM-ECZ 500 MHz spectrometer. Chemical shifts are reported in parts per million using residual non-deuterated solvent or tetramethylsilane (TMS) as an internal standard (TMS: 0.00 ppm for ^{1}H and ^{13}C NMR; $CDCl_3$: 7.26 ppm for ^{1}H NMR and 77.16 ppm for ^{13}C NMR; DMSO-d_6: 2.50 ppm for ^{1}H NMR and 39.52 ppm). Data are reported as follows: chemical shift, multiplicity (ap = apparent, br = broad, s = singlet, d = doublet, t = triplet, q = quartet, quin = quintet, m = multiplet), coupling constant(s) in Hz, integration. NMR spectra were performed at 298 K unless otherwise noted. FT-IR spectra were recorded on a PerkinElmer Frontier FT-IR spectrometer and are reported in terms of frequency of absorption (cm^{-1}).

Melting points (mp) are uncorrected and were measured on a Mel-Temp melting point apparatus. Mass spectra were collected via electrospray ionization in positive ion mode using a

ThermoScientific LTQ Velos Pro linear ion trap mass spectrometer. The samples were dissolved in acetonitrile and infused via syringe pump at a flow rate of 5 μL/min. The spray voltage was set at +4 kV, and the inlet capillary temperature was set to 150 °C. Steady-state UV-Vis spectroscopy was recorded using a Cary 60 UV-Vis spectrophotometer from Agilent Technologies. Transient absorption measurements were performed at on a commercially available spectrometer (Ultrafast Systems EOS). The sample was placed in a quartz cuvette with a 2-mm path length. The excitation source is a tunable, diode-pumped solid-state laser and optical parametric generation (Ekspla PT403) with a pulse width of 20 ps. Pump energies were attenuated using neutral density filters to between 2-10 μJ per pulse. Surface Xplorer (Ultrafast Systems EOS) was used to analyze the data.

High resolution mass spectra (HRMS) for compounds **7**, **9**–**20**, **26**, and **27** were acquired at the Mass Spectrometry Center in the Department of Chemistry and Biochemistry at the University of South Carolina. HRMS were collected on a VG-70S magnetic sector mass spectrometer (Waters) by direct probe introduction and electron ionization at 70 eV or on a Thermo Orbitrap Velos Pro high resolution mass spectrometer operated in positive ion mode at a mass resolution of 30000. High resolution mass spectra (HRMS) for compounds **22** and **23** were acquired at the Mass Spectrometry Core Laboratory in the Department of Chemistry at the University of North Carolina at Chapel Hill. Samples were analyzed with a Q Exactive HF-X (ThermoFisher, Bremen, Germany) mass spectrometer. Samples were introduced via an atmospheric pressure chemical ionization (APCI) at a flow rate of 20 uL/min. APCI source conditions were set as: corona voltage 5.0 kV, sheath gas (nitrogen) 18 arb, auxillary gas (nitrogen) 3 arb, sweep gas (nitrogen) 0 arb, capillary temperature 250 degrees C, nebulizer temperature 350 degrees C, and S lens RF 35. The mass range was set to 100-1000 m/z. All measurements were recorded at a resolution setting of 120,000. Solutions were analyzed at 0.1 mg/mL or less based

on responsiveness to the APCI mechanism. Xcalibur (ThermoFisher, Breman, Germany) was used to analyze the data. Molecular formula assignments were determined with Molecular Formula Calculator (v 1.3.3). All observed species were singly charged, as verified by unit m/z separation between mass spectral peaks corresponding to the 12C and 13C12Cc-1 isotope for each elemental composition.

III. Experimental Procedures and Characterization Data

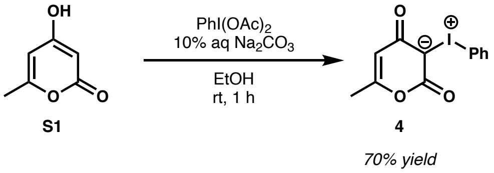

Iodonium Ylide 4. Ylide **4** was prepared according to a modified literature procedure.[1] To a solution of 4-hydroxy-6-methyl-2-pyrone (**S1**) (1.00 g, 7.93 mmol) in a 10% aqueous Na_2CO_3 solution (23.7 mL) was added a solution of phenyliodine(III) diacetate (2.55 g, 7.93 mmol) in ethanol (7.9 mL) *via* syringe. The transfer was completed using an additional three portions of ethanol (3 x 0.5 mL). After allowing the solution to stir for 1 hour, the reaction mixture was diluted with water (10 mL). The aqueous layer was extracted with CH_2Cl_2 (3 x 50 mL) or until all visible ylide was dissolved in the organic layer. The combined organic layers were then dried over $MgSO_4$, filtered, and concentrated *in vacuo*. Recrystallization from hot ethanol (78 ˚C) provided the title compound (1.83 g, 70%) as a white solid (mp 144–146 ˚C, lit.[1] mp 144–146 ˚C). The spectral data for this compound are consistent with those reported in the literature.[2] ^1H NMR (500 MHz, DMSO-d_6) δ 7.78 (ap dd, J = 8.3, 1.3 Hz, 2H), 7.53 (tt, J = 7.4, 1.3 Hz, 1H), 7.42 (ap t, J = 7.8 Hz, 2H), 5.65 (d, J = 1.0 Hz, 1H), 2.06 (d, J = 1.0 Hz, 3H); $^{13}C\{^1H\}$ NMR (126 MHz, DMSO-d_6) δ 176.0, 162.5, 161.5, 132.9, 131.1, 130.7, 115.0, 105.5, 81.4, 18.9.

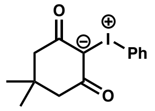

S2

2-Phenyliodonio-5,5-dimethyl-1,3-dioxacyclohexanemethylide (S2). The title compound was prepared according to the literature procedure.[3] The spectral data for this compound are consistent with those reported in the literature.[4] ^{1}H NMR (500 MHz, $CDCl_3$) δ 7.83 (ap dd, *J* = 8.5, 1.0 Hz, 2H), 7.53 (tt, *J* = 7.5, 1.3 Hz, 1H), 7.37 (ap t, *J* = 7.5 Hz, 2H), 2.51 (s, 4H), 1.07 (s, 6H); ^{13}C{^{1}H} NMR (126 MHz, $CDCl_3$)) δ 188.5, 133.9, 131.7, 131.5, 111.9, 94.5, 50.8, 32.1, 28.2.

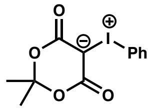

S3

2-Phenyliodonio-5,5-dimethyl-4,6-dioxa-1,3-dioxocyclohexane methylide (S3). The title compound was prepared according to the literature procedure.[3] The spectral data for this compound are consistent with those reported in the literature.[5] ^{1}H NMR (500 MHz, $CDCl_3$) δ 7.90 (ap dd, *J* = 8.3, 1.3 Hz, 2H), 7.60 (tt, *J* = 7.5, 1.0 Hz, 1H), 7.44 (ap t, *J* = 7.3 Hz, 2H), 1.72 (s, 6H); ^{13}C{^{1}H} NMR (126 MHz, $CDCl_3$)) δ 163.5, 133.6, 132.3, 132.1, 114.1, 104.7, 55.9, 25.9.

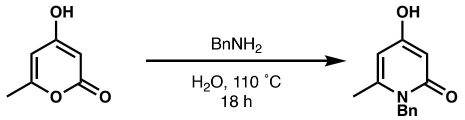

1-Benzyl-4-hydroxy-6-methylpyridin-2(1H)-one (S4). The following procedure was adapted from Vucicevic et al.[6] A suspension of 4-hydroxy-6-methyl-2-pyrone (750 mg, 5.95 mmol) and benzylamine (0.65 mL, 5.95 mmol) in deionized water (24 mL, 0.25 M) was allowed to heat at reflux (110 ºC) for 18 hours. The reaction mixture was allowed to cool to room temperature, filtered under vacuum, and washed using chilled diethyl ether. The crude material was dissolved in ethyl acetate (15 mL) and extracted using saturated sodium carbonate solution (3 x 15 mL). The

combined aqueous extracts were washed with ethyl acetate (1 x 15 mL) and acidified to a pH of 2 by dropwise addition of 12 M HCl. The resulting precipitate was filtered, washed with cold water, and allowed to dry in a dry box overnight, providing the title compound (0.741 g, 58%) as a white solid (mp 204–205 ˚C). The spectral data for this compound are consistent with those reported in the literature.[6] ^{1}H NMR (500 MHz, DMSO-d_6) δ 10.50 (s, 1H), 7.32 (ap t, J = 7.8 Hz, 2H), 7.24 (ap t, J = 7.3 Hz, 1H), 7.08 (ap d, J = 7.0 Hz, 2H), 5.79 (dd, J = 2.5, 1.0 Hz, 1H), 5.59 (d, J = 2.5 Hz, 1H), 5.19 (br s, 2H), 2.16 (s, 3H); ^{13}C{^{1}H} NMR (126 MHz, DMSO-d_6) δ 165.9, 164.0, 147.6, 137.8, 128.6, 126.9, 126.1, 100.4, 95.9, 45.3, 19.9.

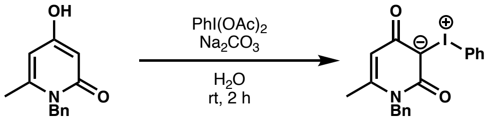

S4 5

98% yield

Iodonium Ylide 5. The title compound was prepared according to the literature procedure.[2] To a solution of 1-benzyl-4-hydroxy-6-methylpyridin-2(1H)-one (**S4**) (0.418 g, 1.94 mmol) and Na_2CO_3 (0.247 g, 1.94 mmol) in H_2O (9.7 mL) was added phenyliodine(III) diacetate (0.625 g, 2.33 mmol) in one portion. After allowing the reaction mixture to stir at room temperature for 2 hours, the precipitate was filtered, rinsed with water, and dried under vacuum. The title compound (0.791 mg, 98%) was afforded as a white solid (mp 205–206 ˚C) and used without further purification. The spectral data for this compound are consistent with those reported in the literature.[2] ^{1}H NMR (500 MHz, DMSO-d_6) δ 7.77 (d, J = 8.5 Hz, 2H), 7.51 (t, J = 7.25 Hz, 1H), 7.42 (t, J = 8.0 Hz, 2H), 7.31 (t, J = 7.5 Hz, 2H), 7.23 (t, J = 7.5 Hz, 1H), 7.10 (d, J = 7.5 Hz, 2H), 5.58 (s, 1H), 5.15 (br s, 2H), 2.08 (s, 3H); ^{13}C{^{1}H} NMR (126 MHz, DMSO-d_6) δ 174.5, 162.6, 147.4, 138.8, 132.3, 130.9, 130.3, 128.5, 126.8, 126.0, 114.7, 106.5, 90.8, 46.1, 19.7.

General Procedure A: [3+2] Photocycloaddition of Iodonium Ylides and Alkenes

To an oven dried 4-dram vial were added the iodonium ylide (1 equiv) and eosin Y (5 mol%). The vial was sealed with a Teflon lined screwcap and a septum and flushed with nitrogen for ~10 minutes. Acetonitrile (0.1 M) and styrene (4 equiv) were added *via* syringe. The vial was then placed in a heating block set to 60 ˚C and irradiated with two 525 nm Kessil lamps at a distance of 2 cm for 4 h. After removing the reaction mixture from the heating block and allowing to cool to room temperature, the reaction mixture was concentrated *in vacuo.* The crude residue was dissolved in EtOAc and filtered through a pad of silica (elution 30:1 EtOAc/Et_3N). The filtrate was concentrated *in vacuo*, and the crude residue was purified using automated flash chromatography, yielding the desired cycloadduct.

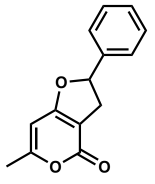

6

6-Methyl-2-phenyl-2,3-dihydro-4*H*-furo[3,2-*c*]pyran-4-one (6). The title compound was prepared according to General Procedure A using iodonium ylide **4** (197 mg, 0.600 mmol), eosin Y (19 mg, 0.030 mmol), styrene (0.27 mL, 2.40 mmol), and MeCN (6 mL). The crude material was purified by automated flash chromatography (SiO_2, 0–2% acetone in CH_2Cl_2 gradient) to yield the title compound (67 mg, 49%) as a viscous yellow oil. The spectral data for this compound are consistent with those reported in the literature.[7] ^{1}H NMR ($CDCl_3$, 500 MHz) δ 7.44 – 7.31 (m, 5H), 6.00 (d, J = 1.0 Hz, 1H), 5.89 (dd, J = 10.3, 7.8 Hz, 1H), 3.48 (dd, J = 15.3, 10.3 Hz, 1H), 3.06 (dd, J = 15.0, 8.0 Hz, 1H), 2.29 (s, 3H); ^{13}C{^{1}H} NMR (126 MHz, $CDCl_3$) δ 171.2, 165.6, 162.1, 140.0, 129.0, 126.0, 99.2, 95.7, 87.6, 34.0, 20.6.

1 mmol Scale Reaction: 6-Methyl-2-phenyl-2,3-dihydro-4*H*-furo[3,2-*c*]pyran-4-one (6). The title compound was prepared according to General Procedure A using iodonium ylide **4** (0.328 g, 1.00 mmol), eosin Y (0.032 g, 0.050 mmol), styrene (0.46 mL, 4.00 mmol), and MeCN (10 mL). The crude material was purified by automated flash chromatography (SiO_2, 0–2% acetone in CH_2Cl_2 gradient) to yield the title compound (94 mg, 41%) as a viscous yellow oil. The spectral data for this compound are consistent with those reported above.

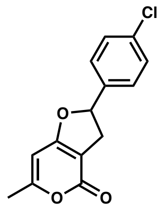

9

2-(4-Chlorophenyl)-6-methyl-2,3-dihydro-4*H*-furo[3,2-*c*]pyran-4-one (9). The title compound was prepared according to General Procedure A using iodonium ylide **4** (197 mg, 0.600 mmol), eosin Y (19 mg, 0.030 mmol), 4-chlorostyrene (0.29 mL, 2.40 mmol), and MeCN (6 mL). The crude material was purified by automated flash chromatography (SiO_2, 0–2% acetone in CH_2Cl_2 gradient) to yield the title compound (62 mg, 40%) as a reddish amorphous solid. The spectral data for this compound are consistent with those reported in the literature.[7] ^{1}H NMR ($CDCl_3$, 500 MHz) δ 7.38 (d, J = 8.5 Hz, 2H), 7.28 (d, J = 8.5 Hz, 2H), 6.00 (s, 1H), 5.86 (dd, J = 10.3, 7.8 Hz, 1H), 3.48 (dd, J = 15.0, 10.5 Hz, 1H), 3.01 (dd, J = 15.0, 8.0 Hz, 1H), 2.29 (s, 3H); ^{13}C{^{1}H} NMR (126 MHz, $CDCl_3$) δ 171.2, 165.9, 162.1, 138.6, 134.9, 129.3, 127.4, 99.1, 95.7, 86.7, 34.1, 20.6; IR (ATR) ν 3083, 3033, 2953, 2939, 2919, 2874, 1715, 1638, 1579, 1254, 1086, 979, 829, 815 cm^{-1}; HRMS (EI) m / z calcd for $C_{14}H_{11}ClO_3$ $(M)^+$ 262.0397, found 262.0387.

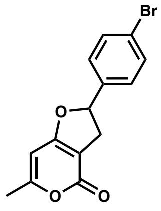

10

2-(4-Bromophenyl)-6-methyl-2,3-dihydro-4*H*-furo[3,2-*c*]pyran-4-one (10). The title compound was prepared according to General Procedure A using iodonium ylide **4** (197 mg, 0.600 mmol), eosin Y (19 mg, 0.030 mmol), 4-bromostyrene (0.31 mL, 2.40 mmol), and MeCN (6 mL). The crude material was purified by automated flash chromatography (SiO_2, 0–2% acetone in CH_2Cl_2 gradient) to yield the title compound (105 mg, 57%) as an orange solid (mp 123–124 ˚C). ^{1}H NMR ($CDCl_3$, 500 MHz) δ 7.53 (d, J = 8.5 Hz, 2H), 7.22 (d, J = 8.5 Hz, 2H), 6.00 (s, 1H), 5.85 (dd, J = 10.5, 8.0 Hz, 1H), 3.48 (dd, J = 15.0, 10.5 Hz, 1H), 3.00 (dd, J = 15.0, 8.0 Hz, 1H), 2.29 (s, 3H); $^{13}C\{^1H\}$ NMR (126 MHz, $CDCl_3$) δ 171.0, 165.7, 161.9, 139.0, 132.1, 127.5, 122.8, 99.0, 95.5, 86.6, 33.9, 20.5; IR (ATR) ν 3088, 2952, 2873, 1708, 1632, 1579, 1259, 1102, 977, 920, 822 cm^{-1}; HRMS (EI) m / z calcd for $C_{14}H_{11}BrO_3$ $(M)^+$ 305.9892, found 305.9895.

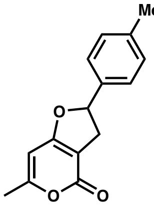

11

6-Methyl-2-(4-methylphenyl)-2,3-dihydro-4*H*-furo[3,2-*c*]pyran-4-one (11). The title compound was prepared according to General Procedure A using iodonium ylide **4** (197 mg, 0.600 mmol), eosin Y (19 mg, 0.030 mmol), 4-methylstyrene (0.32 mL, 2.40 mmol), and MeCN (6 mL). The crude material was purified by automated flash chromatography (SiO_2, 0–2% acetone in CH_2Cl_2 gradient) to yield the title compound (63 mg, 43%) as a yellow-orange viscous oil. The spectral data for this compound are consistent with those reported in the literature.[7] ^{1}H NMR

($CDCl_3$, 500 MHz) δ 7.24 (d, J = 8.5 Hz, 2H), 7.21 (d, J = 8.0 Hz, 2H), 5.98 (d, J = 0.5 Hz, 1H), 5.86 (dd, J = 10.3, 7.8 Hz, 1H), 3.45 (dd, J = 15.0, 10.0 Hz, 1H), 3.06 (dd, J = 15.0, 8.0 Hz, 1H), 2.37 (s, 3H), 2.28 (d, J = 1.0 Hz, 3H); $^{13}C\{^1H\}$ NMR (126 MHz, $CDCl_3$) δ 171.1, 165.4, 162.1, 138.9, 136.8, 129.6, 126.0, 99.2, 95.7, 87.6, 33.7, 21.2, 20.4; IR (thin film) ν 3087, 3027, 2923, 2867, 1708, 1637, 1578, 1450, 1254, 976, 813 cm^{-1}; HRMS (EI) *m* / z calcd for $C_{15}H_{14}O_3$ $(M)^+$ 242.0943, found 242.0950.

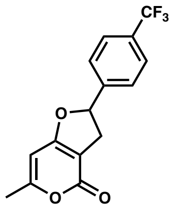

12

6-Methyl-2-(4-trifluoromethylphenyl)-2,3-dihydro-4*H*-furo[3,2-*c*]pyran-4-one (12). The title compound was prepared according to General Procedure A using iodonium ylide **4** (197 mg, 0.600 mmol), eosin Y (19 mg, 0.030 mmol), 4-trifluoromethylstyrene (0.35 mL, 2.40 mmol), and MeCN (6 mL). The crude material was purified by automated flash chromatography (SiO_2, Column 1: 0–2% acetone in CH_2Cl_2 gradient; Column 2: 0–60% EtOAc in pentane gradient) to yield the title compound (58 mg, 33%) as a yellow viscous oil. ^1H NMR ($CDCl_3$, 500 MHz) δ 7.66 (d, J = 8.5 Hz, 2H), 7.46 (d, J = 8.0 Hz, 2H), 6.03 (br s, 1H), 5.94 (dd, J = 10.3, 7.8 Hz, 1H), 3.53 (dd, J = 15.3, 10.3 Hz, 1H), 3.01 (dd, J = 15.3, 7.8 Hz, 1H), 2.30 (br s, 3H); $^{13}C\{^1H\}$ NMR (126 MHz, $CDCl_3$) δ 171.2, 166.0, 162.0, 144.0 (q, J = 1.5 Hz), 131.1 (q, J = 32.6 Hz), 126.2, 126.1 (q, J = 3.9 Hz), 124.0 (q, J = 272.8 Hz), 99.0, 95.6, 86.4, 34.4, 20.6; ^{19}F NMR (471 MHz, $CDCl_3$) δ –62.6 (s); IR (thin film) ν 3083, 2930, 2873, 1715, 1641, 1622, 1582, 1452, 1415, 1322, 1109, 1066, 977, 839 cm^{-1}; HRMS (EI) *m* / z calcd for $C_{15}H_{11}F_3O_3$ $(M)^+$ 296.0660, found 296.0672.

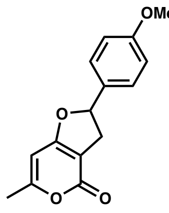

13

2-(4-Methoxyphenyl)-6-methyl-2,3-dihydro-4*H*-furo[3,2-*c*]pyran-4-one (13). The title compound was prepared according to General Procedure A using iodonium ylide **4** (197 mg, 0.600 mmol), eosin Y (19 mg, 0.030 mmol), 4-methoxystyrene (0.32 mL, 2.40 mmol), and MeCN (6 mL). The crude material was purified by automated flash chromatography (SiO_2, Column 1: 10–80% EtOAc in hexane gradient; Column 2: 3–30% *i*PrOH in hexane gradient; Column 3: 10–80% EtOAc in pentane gradient) to yield the title compound (0.010 g, 7%) as a pale yellow solid (mp 129–130 ˚C). ^1H NMR ($CDCl_3$, 500 MHz) δ 7.28 (d, *J* = 9.0 Hz, 2H), 6.92 (d, *J* = 8.5 Hz, 2H), 5.97 (s, 1H), 5.84 (dd, *J* = 10.0, 8.0 Hz, 1H), 3.82 (s, 3H), 3.43 (dd, *J* = 15.3, 10.3 Hz, 1H), 3.07 (dd, *J* = 15.5, 8.0 Hz, 1H), 2.28 (s, 3H); $^{13}C\{^1H\}$ NMR (126 MHz, $CDCl_3$) δ 171.1, 165.6, 162.2, 160.2, 131.9, 127.8, 114.4, 99.3, 95.8, 87.7, 55.5, 33.7, 20.5; IR (ATR) ν 3080, 3001, 2957, 2933, 2838, 1712, 1638, 1579, 1515, 1451, 1247, 1177, 978, 832 cm^{-1}; HRMS (EI) *m* / z calcd for $C_{15}H_{14}O_4$ $(M)^+$ 258.0892, found 258.0902.

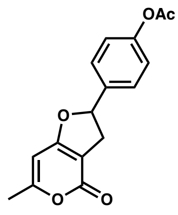

14

2-(4-Acetoxyphenyl)-6-methyl-2,3-dihydro-4*H*-furo[3,2-*c*]pyran-4-one (14). The title compound was prepared according to General Procedure A using iodonium ylide **4** (197 mg, 0.600 mmol), eosin Y (19 mg, 0.030 mmol), 4-acetoxystyrene (0.37 mL, 2.40 mmol), and MeCN (6 mL). The crude material was purified by automated flash chromatography (SiO_2, 0–2% acetone in

CH_2Cl_2 gradient) to yield the title compound (91 mg, 53%) as a yellow-orange viscous oil. ^1H NMR ($CDCl_3$, 500 MHz) δ 7.37 (d, *J* = 8.5 Hz, 2H), 7.13 (d, *J* = 8.5 Hz, 2H), 5.99 (d, *J* = 1.0 Hz, 1H), 5.89 (dd, *J* = 10.5, 8.0 Hz, 1H), 3.48 (dd, *J* = 15.3, 10.3 Hz, 1H), 3.06 (dd, *J* = 15.0, 8.0 Hz, 1H), 2.31 (s, 3H), 2.29 (d, *J* = 0.5 Hz, 3H); $^{13}C\{^1H\}$ NMR (126 MHz, $CDCl_3$) δ 171.0, 169.3, 165.6, 161.9, 150.9, 137.5, 127.2, 122.1, 99.0, 95.6, 86.8, 33.9, 21.1, 20.5; IR (thin film) ν 3080, 2926, 2870, 1754, 1710, 1638, 1579, 1508, 1452, 1189, 1165, 978, 909, 728 cm^{-1}; HRMS (EI) *m* / z calcd for $C_{16}H_{14}O_5$ $(M)^+$ 286.0841, found 286.0837.

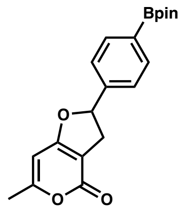

15

6-Methyl-2-(4-(4,4,5,5-tetramethyl-1,3,2-dioxaborolan-2-yl)phenyl)-2,3-dihydro-4*H*-furo[3,2-*c*]pyran-4-one (15). The title compound was prepared according to General Procedure A using iodonium ylide **4** (197 mg, 0.600 mmol), eosin Y (19 mg, 0.030 mmol), 4,4,5,5-tetramethyl-2-(4-vinylphenyl)-1,3,2-dioxaborolane (0.63 mL, 2.40 mmol), and MeCN (6 mL). The crude material was purified by automated flash chromatography (SiO_2, 0–2% acetone in CH_2Cl_2 gradient) to yield the title compound (7.6 mg, 4%) as a yellow-orange viscous oil. ^1H NMR ($CDCl_3$, 500 MHz) δ 7.84 (d, *J* = 8.0 Hz, 2H), 7.33 (d, *J* = 8.0 Hz, 2H), 6.01 (d, *J* = 1.0 Hz, 1H), 5.90 (dd, *J* = 10.5, 8.0 Hz, 1H), 3.48 (dd, *J* = 15.0, 10.5 Hz, 1H), 3.03 (dd, *J* = 15.0, 8.0 Hz, 1H), 2.29 (d, *J* = 1.0 Hz, 3H), 1.35 (s, 12H); $^{13}C\{^1H\}$ NMR (126 MHz, $CDCl_3$) δ 171.2, 165.5, 162.1, 142.8, 135.3, 125.0, 99.1, 95.7, 87.3, 84.0, 34.0, 24.9, 20.5 (The carbon directly attached to the boron atom was not detected owing to quadrupolar broadening); IR (thin film) ν 2980, 2929, 1714,

1641, 1614, 1583, 1259, 1143, 1088, 906, 725 cm^{-1}; HRMS (EI) *m* / z calcd for $C_{20}H_{23}BO_5$ $(M)^+$ 354.1639, found 354.1645.

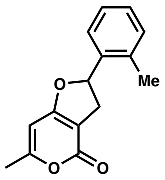

16

6-Methyl-2-(2-methylphenyl)-2,3-dihydro-4*H*-furo[3,2-*c*]pyran-4-one (16). The title compound was prepared according to General Procedure A using iodonium ylide **4** (197 mg, 0.600 mmol), eosin Y (19 mg, 0.030 mmol), 2-methylstyrene (0.31 mL, 2.40 mmol), and MeCN (6 mL). The crude material was purified by automated flash chromatography (SiO_2, 0–2% acetone in CH_2Cl_2 gradient) to yield the title compound (91 mg, 63%) as a reddish viscous oil. ^{1}H NMR ($CDCl_3$, 500 MHz) δ 7.31–7.19 (m, 4H), 6.09 (dd, *J* = 10.5, 8.0 Hz, 1H), 6.04 (s, 1H), 3.49 (dd, *J* = 14.8, 10.3 Hz, 1H), 2.93 (dd, *J* = 15.3, 8.3 Hz, 1H), 2.34 (s, 3H), 2.30 (s, 3H); $^{13}C\{^1H\}$ NMR (126 MHz, $CDCl_3$) δ 171.4, 165.7, 162.2, 138.3, 134.6, 131.0, 128.6, 126.6, 124.9, 99.2, 95.8, 85.2, 33.4, 20.6, 19.3; IR (thin film) ν 3080, 2925, 1706, 1632, 1578, 1448, 1258, 976, 919, 742 cm^{-1}; HRMS (EI) *m* / z calcd for $C_{15}H_{14}O_3$ $(M)^+$ 242.0943, found 242.0935.

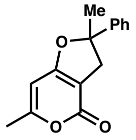

17

2,6-Dimethyl-2-phenyl-2,3-dihydro-4*H*-furo[3,2-*c*]pyran-4-one (17). The title compound was prepared according to General Procedure A using iodonium ylide **4** (197 mg, 0.600 mmol), eosin Y (19 mg, 0.030 mmol), α-methylstyrene (0.31 mL, 2.40 mmol), and MeCN (6 mL). The crude material was purified by automated flash chromatography (SiO_2, Column 1: 6–50% EtOAc in hexane gradient; Column 2: 0–2% acetone in CH_2Cl_2) to yield the title compound (19 mg, 13%)

as a white solid (mp 86–87 ˚C, lit.[7] mp 91–92 ˚C). The spectral data for this compound are consistent with those reported in the literature.[7] ^{1}H NMR ($CDCl_3$, 500 MHz) δ 7.38 (ap d, *J* = 4.5 Hz, 4H), 7.31 (ap sextet, *J* = 4.5 Hz, 1H), 6.02 (s, 1H), 3.27 (d, *J* = 14.5 Hz, 1H), 3.18 (d, *J* = 14.5 Hz, 1H), 2.28 (s, 3H), 1.79 (s, 3H); $^{13}C\{^{1}H\}$ NMR (126 MHz, $CDCl_3$) δ 170.2, 165.5, 162.3, 144.9, 128.7, 127.9, 124.3, 98.9, 96.0, 94.2, 40.6, 29.5, 20.6; IR (thin film) ν 3086, 2974, 2926, 2866, 1719, 1639, 1582, 1447, 1271, 979, 700 cm^{-1}; HRMS (EI) *m* / z calcd for $C_{15}H_{14}O_3$ $(M)^+$ 242.0943, found 242.0945.

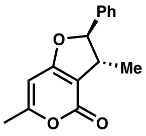

18

(2*R,3*R**)-3,6-Dimethyl-2-phenyl-2,3-dihydro-4*H*-furo[3,2-*c*]pyran-4-one (18).** The title compound was prepared according to General Procedure A using iodonium ylide **4** (197 mg, 0.600 mmol), eosin Y (19 mg, 0.030 mmol), β-methylstyrene (0.31 mL, 2.40 mmol), and MeCN (6 mL). The crude material was purified by automated flash chromatography (SiO_2, Column 1: 6–50% EtOAc in hexane gradient; Column 2: 0–10% *i*PrOH in pentane gradient; Column 3: 0–4% % *i*PrOH in CH_2Cl_2 gradient) to yield the title compound (68 mg, 47%) as a yellow viscous oil. ^{1}H NMR ($CDCl_3$, 500 MHz) δ 7.44–7.34 (m, 3H), 7.34–7.29 (m, 2H), 5.99 (s, 1H), 5.30 (d, *J* = 7.0 Hz, 1H), 3.42 (quin, *J* = 7.0 Hz, 1H), 2.28 (s, 3H), 1.47 (d, *J* = 7.0 Hz, 3H); $^{13}C\{^{1}H\}$ NMR (126 MHz, $CDCl_3$) δ 170.6, 165.8, 162.0, 139.5, 129.02, 129.00, 125.9, 103.7, 95.8, 95.1, 42.9, 20.6, 18.4; IR (thin film) ν 3088, 3034, 2962, 2926, 2872, 1707, 1633, 1578, 1445, 1416, 1247, 977, 920, 892, 740, 697 cm^{-1}; HRMS (EI) *m* / z calcd for $C_{15}H_{14}O_3$ $(M)^+$ 242.0943, found 242.0952. The *J* value for the protons indicated below (*J* = 7.0 Hz) is consistent with the *trans*-product.

Formation of only the *trans*-product is consistent with related oxidative cycloaddition reactions of 4-hydroxycoumarin and β-methylstyrene.[8]

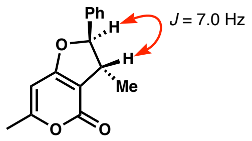

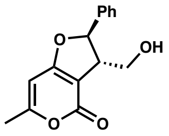

19

(2*R,3*S**)-3-Hydroxymethyl-6-methyl-2-phenyl-2,3-dihydro-4*H*-furo[3,2-*c*]pyran-4-one (19).** The title compound was prepared according to General Procedure A using iodonium ylide **4** (197 mg, 0.600 mmol), eosin Y (19 mg, 0.030 mmol), cinnamyl alcohol (0.32 g, 2.40 mmol), and MeCN (6 mL). The crude material was purified by automated flash chromatography (SiO_2, Column 1: 10–80% EtOAc in hexane gradient; Column 2: 2–18% acetone in CH_2Cl_2 gradient) to yield the title compound (0.020 g, 13%) as a yellow viscous oil. ^1H NMR ($CDCl_3$, 500 MHz) δ 7.45–7.32 (m, 5H), 6.05 (s, 1H), 5.48 (d, J = 8.0 Hz, 1H), 3.92 (t, J = 5.8 Hz, 2H), 3.67–3.57 (m, 2H), 2.32 (s, 3H); $^{13}C\{^1H\}$ NMR (126 MHz, $CDCl_3$) δ 171.9, 166.2, 163.4, 138.8, 129.1, 129.0, 126.0, 101.4, 96.1, 90.0, 64.0, 50.7, 20.5; IR (thin film) ν 3416, 3089, 3067, 3034, 2926, 2877, 1698, 1638, 1580, 1450, 1256, 1070, 1029, 982, 760, 699 cm^{-1}; HRMS (EI) m / z calcd for $C_{15}H_{14}O_4$ $(M)^+$ 258.0892, found 258.0897. The J value for the protons indicated below (J = 8.0 Hz) is consistent with the *trans*-product. Formation of only the *trans*-product is consistent with related oxidative cycloaddition reactions of 4-hydroxycoumarin and 1,2-disubstituted alkenes.[8]

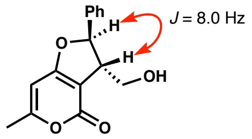

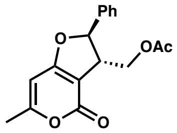

20

((2*R,3*S**)-6-Methyl-4-oxo-2-phenyl-2,3-dihydro-4*H*-furo[3,2-*c*]pyran-3-yl)methyl acetate (20).** The title compound was prepared according to General Procedure A using iodonium ylide **4** (197 mg, 0.600 mmol), eosin Y (19 mg, 0.030 mmol), cinnamyl acetate (0.40 mL, 2.40 mmol), and MeCN (6 mL). The crude material was purified by automated flash chromatography (SiO_2, Column 1: 0–6% MeOH in CH_2Cl_2 gradient; Column 2: 1–8% acetone in CH_2Cl_2 gradient) to yield the title compound (23 mg, 13%) as a colorless oil. ^{1}H NMR ($CDCl_3$, 500 MHz) δ 7.43–7.34 (m, 3H), 7.32–7.28 (m, 2H), 6.03 (s, 1H), 5.65 (d, J = 6.0 Hz, 1H), 4.57 (dd, J = 11.3, 3.8 Hz, 1H), 4.35 (dd, J = 11.0, 7.5 Hz, 1H), 3.69 (td, J = 7.4, 3.7 Hz, 1H), 2.31 (s, 3H), 2.06 (s, 3H); ^{13}C{^{1}H} NMR (126 MHz, $CDCl_3$) δ 171.9, 171.0, 166.7, 161.5, 139.4, 129.10, 129.09, 125.7, 98.4, 95.7, 90.6, 63.8, 47.7, 21.0, 20.7; IR (thin film) ν 3072, 3032, 2956, 2923, 2852, 1736, 1712, 1634, 1579, 1449, 1241, 1229, 1056, 978, 901, 763, 710 cm^{-1}; HRMS (EI) m / z calcd for $C_{17}H_{16}O_5$ (M)$^+$ 300.0998, found 300.0997. The J value for the protons indicated below (J = 6.0 Hz) is consistent with the *trans*-product. Formation of only the *trans*-product is consistent with related oxidative cycloaddition reactions of 4-hydroxycoumarin and 1,2-disubstituted alkenes.[8]

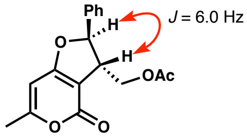

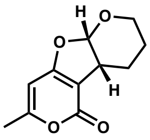

22

(4a*R,9a*S**)-7-methyl-3,4,4a,9a-tetrahydro-2*H*,5*H*-furo[2,3-*b*:4,5-*c*']dipyran-5-one (22).** The title compound was prepared according to General Procedure A using iodonium ylide **4** (197 mg, 0.600 mmol), eosin Y (19 mg, 0.030 mmol), 3,4-dihydro-2*H*-pyran (0.22 mL, 2.40 mmol), and MeCN (6 mL). The crude material was purified by automated flash chromatography (SiO_2, 10–50% EtOAc in hexane gradient) to yield the title compound (12 mg, 10%) as a yellow oil. ^1H NMR ($CDCl_3$, 500 MHz) δ 6.10 (d, J = 7.5 Hz, 1H), 5.99 (s, 1H), 3.84 (ddd, J = 11.5, 7.5, 5.5 Hz, 1H), 3.76 (ap dt, J = 11.3, 7.0 Hz, 1H), 3.33 (ap q, J = 6.0 Hz, 1H), 2.28 (s, 3H), 1.99–1.94 (m, 2H), 1.78–1.69 (m, 1H), 1.66–1.56 (m, 1H); $^{13}C\{^1H\}$ NMR (126 MHz, $CDCl_3$) δ 170.3, 165.8, 161.7, 107.6, 101.7, 95.6, 60.5, 35.1, 20.5, 19.3, 18.8; IR (thin film) ν 3091, 2952, 1710, 1636, 1577, 1451, 1236, 1118, 977, 837 cm^{-1}; HRMS (APCI) m / z calcd for $C_{11}H_{13}O_4$ $(M+H)^+$ 209.0814, found 209.0809.

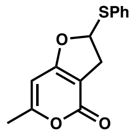

23

6-Methyl-2-(phenylthio)-2,3-dihydro-4*H*-furo[3,2-*c*]pyran-4-one (23). The title compound was prepared according to General Procedure A using iodonium ylide **4** (197 mg, 0.600 mmol), eosin Y (19 mg, 0.030 mmol), phenyl vinyl sulfide (0.31 mL, 2.40 mmol), and MeCN (6 mL). The crude material was purified by automated flash chromatography (SiO_2, Column 1: 8–70% EtOAc in hexane gradient; Column 2: 0–2% acetone in CH_2Cl_2 gradient) to yield the title compound (0.040 g, 26%) as a white amorphous solid. ^1H NMR ($CDCl_3$, 500 MHz) δ 7.55–7.51 (m, 2H), 7.38–7.33 (m, 3H), 6.23 (dd, J = 9.8, 6.3 Hz, 1H), 5.97 (s, 1H), 3.48 (dd, J = 16.0, 9.5 Hz, 1H),

3.00 (dd, J = 10.6, 6.5 Hz, 1H), 2.27 (s, 3H); $^{13}C\{^1H\}$ NMR (126 MHz, $CDCl_3$) δ 170.0, 165.6, 161.5, 132.4, 132.2, 129.2, 128.5, 99.3, 95.7, 92.7, 32.7, 20.5; IR (thin film) ν 3079, 2923, 2856, 1709, 1639, 1580, 1448, 1415, 1251, 1165, 1104, 1025, 974, 924, 876, 781, 742, 691 cm^{-1}; HRMS (APCI) *m* / z calcd for $C_{14}H_{13}O_3S$ $(M+H)^+$ 261.0585, found 261.0580.

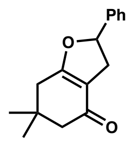

25

6,6-Dimethyl-2-phenyl-2,3,6,7-tetrahydrobenzofuran-4(5*H*)-one (25). The title compound was prepared according to General Procedure A using iodonium ylide **S2** (205 mg, 0.600 mmol), eosin Y (19 mg, 0.030 mmol), styrene (0.27 mL, 2.40 mmol), and MeCN (6 mL). The crude material was purified by automated flash chromatography (SiO_2, 7–60% EtOAc in hexane gradient) to yield the title compound (77 mg, 53%) as a yellow oil. The spectral data for this compound are consistent with those reported in the literature.[9] ^1H NMR ($CDCl_3$, 500 MHz) δ 7.42–7.37 (m, 2H), 7.37–7.20 (m, 3H), 5.77 (dd, J = 10.5, 8.0 Hz, 1H), 3.30 (ddt, J = 14.0, 10.5, 2.0 Hz, 1H), 2.89 (ddt, J = 14.5, 8.0, 2.0 Hz, 1H), 2.38 (t, J = 2.0 H, 2H), 2.29, 2.27 (ABq, J_{AB} = 16.3 Hz, 2H), 1.15 (s, 3H), 1.13 (s, 3H); $^{13}C\{^1H\}$ NMR (126 MHz, $CDCl_3$) δ 194.8, 176.1, 140.7, 128.8, 128.5, 125.8, 111.5, 86.6, 51.0, 37.8, 34.2, 33.9, 28.9, 28.6.

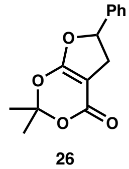

26

2,2-Dimethyl-6-phenyl-5,6-dihydro-4*H*-furo[2,3-*d*][1,3]dioxin-4-one (26). The title compound was prepared according to General Procedure A using iodonium ylide **S3** (208 mg, 0.600 mmol), eosin Y (19 mg, 0.030 mmol), styrene (0.27 mL, 2.40 mmol), and MeCN (6 mL). The crude material was purified by automated flash chromatography (SiO_2, 3–30% EtOAc in hexane

gradient) to yield the title compound (107 mg, 72%) as a white amorphous solid. ^{1}H NMR ($CDCl_3$, 500 MHz) δ 7.38–7.30 (m, 5H), 3.45 (t, J = 9.3 Hz, 1H), 2.70 (dd, J = 9.3, 4.8 Hz, 1H), 2.55 (dd, J = 9.6, 4.5 Hz, 1H), 1.734 (s, 3H), 1.727 (s, 3H); ^{13}C{^{1}H} NMR (126 MHz, $CDCl_3$) δ 167.8, 163.5, 131.1, 129.5, 128.8, 128.4, 105.0, 44.6, 33.1, 28.0, 27.7, 22.9; IR (ATR) ν 3063, 3065, 3006, 2949, 1761, 1730, 1502, 1459, 1436, 1398, 1385, 1322, 1289, 1223, 1120, 1175, 1051, 1035, 1020, 958, 874, 835, 781, 765, 719, 697, 661 cm^{-1}; HRMS (ESI) *m* / z calcd for $C_{14}H_{15}O_4$ $(M+H)^+$ 247.0965, found 247.0961.

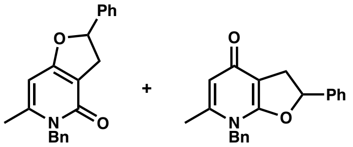

7 27

5-Benzyl-6-methyl-2-phenyl-3,5-dihydro-4*H*-furo[3,2-*c*]pyridin-4(2*H*)-one (7) and 7-Benzyl-6-methyl-2-phenyl-3,7-dihydrofuro[2,3-*b*]pyridin-4(2*H*)-one (27). The title compounds were prepared according to a modified General Procedure A using iodonium ylide **5** (0.250 g, 0.600 mmol), eosin Y (19 mg, 0.030 mmol), styrene (0.27 mL, 2.40 mmol), and MeCN (6 mL). After irradiation for 4 h at 60 ˚C with two 525 nm Kessil lamps at a distance of 2 cm, the reaction mixture was concentrated *in vacuo.* The crude material was purified by automated flash chromatography (SiO_2, 50% 3:1 EtOAc:Ethanol in hexanes to separate **7** and **27**; furo[3,2-*c*]pyridine-4(2*H*)-one **7** further purified using 5–40% EtOAc in pentane gradient followed by 10–100% *tert*-butyl methyl ether in hexane gradient; furo[2,3-*b*]pyridine-4(2*H*)-one **27** further purified using 0–10% acetone in CH_2Cl_2 gradient) to yield furo[3,2-*c*]pyridine-4(2*H*)-one **7** (3.4 mg, 2%) as a yellow oil and furo[2,3-*b*]pyridine-4(2*H*)-one **27** (33 mg, 17%) as a yellow oil.

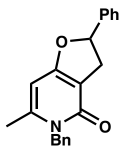

7

Furo[3,2-*c*]pyridine-4(2*H*)-one **7**: ^{1}H NMR ($CDCl_3$, 500 MHz) δ 7.42–7.37 (m, 4H), 7.36–7.28 (m, 3H), 7.26–7.22 (m, 1H), 7.17 (d, J = 7.0 Hz, 2H), 5.94 (s, 1H), 5.86 (dd, J = 10.0, 8.0 Hz, 1H), 5.40 (d, J = 15.5 Hz, 1H), 5.29 (d, J = 15.5 Hz, 1H), 3.60 (dd, J = 15.5, 10.5 Hz, 1H), 3.17 (dd, J = 15.3, 7.8 Hz, 1H), 2.28 (s, 3H); $^{13}C\{^{1}H\}$ NMR (126 MHz, $CDCl_3$) δ 166.8, 161.8, 148.3, 141.0, 137.0, 128.76, 128.75, 128.4, 127.2, 126.4, 125.9, 106.1, 95.8, 86.4, 46.7, 35.4, 21.3; IR (thin film) ν 3063, 3032, 2924, 2854, 1655, 1567, 1453, 1433, 1326, 1256, 1104, 952, 699 cm^{-1}; HRMS (ESI) *m* / z calcd for $C_{21}H_{20}NO_2$ $(M+H)^+$ 318.1489, found 318.1488.

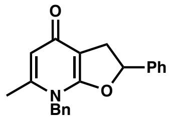

27

Furo[2,3-*b*]pyridine-4(2*H*)-one **27**: ^{1}H NMR ($CDCl_3$, 500 MHz) δ 7.37–7.24 (m, 8H), 7.07 (d, J = 6.5 Hz, 2H), 6.14 (s, 1H), 5.88 (dd, J = 10.0, 7.5 Hz, 1H), 5.14, 5.11 (ABq, J_{AB} = 16.5 Hz, 2H), 3.67 (dd, J = 14.9, 10 Hz, 1H), 3.26 (dd, J = 15.0, 7.5 Hz, 1H), 2.22 (s, 3H); $^{13}C\{^{1}H\}$ NMR (126 MHz, $CDCl_3$) δ 176.4, 160.1, 143.6, 139.9, 135.4, 129.1, 128.79, 128.74, 128.0, 125.89, 125.84, 117.4, 103.9, 86.5, 48.8, 34.9, 18.6; IR (thin film) ν 3088, 3063, 3032, 3006, 2959, 2927, 2868, 1646, 1575, 1533, 1508, 1454, 1348, 1220, 733, 699 cm^{-1}; HRMS (ESI) *m* / z calcd for $C_{21}H_{20}NO_2$ $(M+H)^+$ 318.1489, found 318.1488.

IV. Radical Trapping Experiment

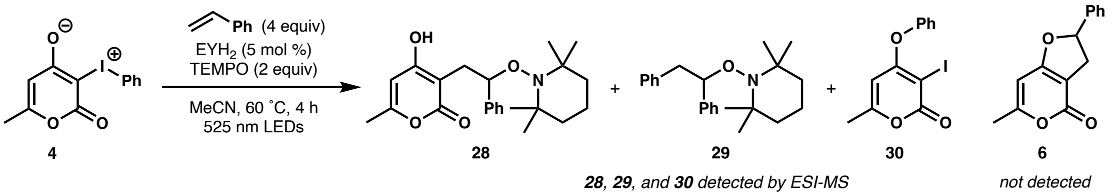

To an oven-dried, 1-dram vial was added ylide **4** (49.2 mg, 0.150 mmol), eosin Y (4.9 mg, 0.0075 mmol), and TEMPO (46.9 mg, 0.300 mmol). The vial was sealed with a Teflon lined screwcap and a septum and flushed with nitrogen for ~10 minutes. Acetonitrile (1.5 mL) and styrene (0.07 mL, 0.60 mmol) were added via syringe. The vial was placed in a heating block set to 60 ˚C and irradiated with one 525 nm Kessil lamp at a distance of 2 cm for 4 h. An aliquot of the reaction mixture was diluted using MeCN prior to analysis by ESI-MS. Furopyranone **6** was not detected nor observed in the crude ^{1}H NMR spectrum. TEMPO adducts **28** and **29** and 3-iodo-4-phenoxy-2-pyrone (**30**) were detected by ESI-MS.

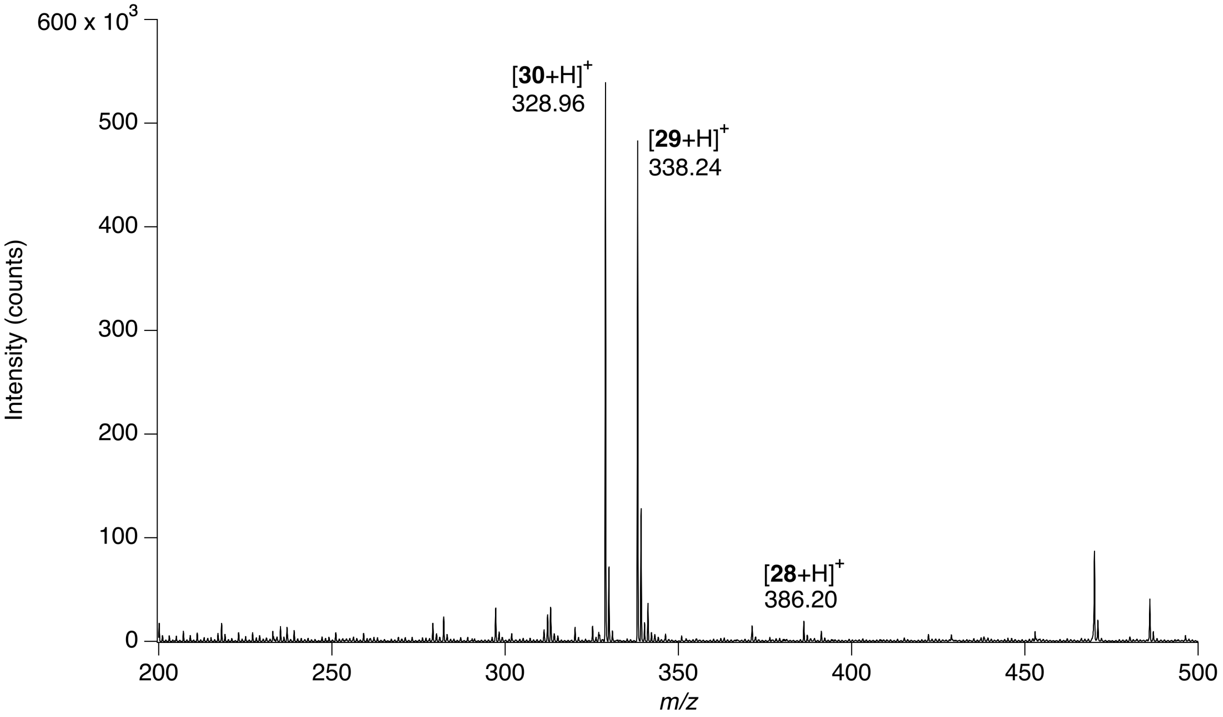

Figure S1. ESI-MS of the crude reaction mixture from the [3+2] photocycloaddition reaction in the presence of TEMPO.

V. Steady-State UV–Vis Absorption Spectra

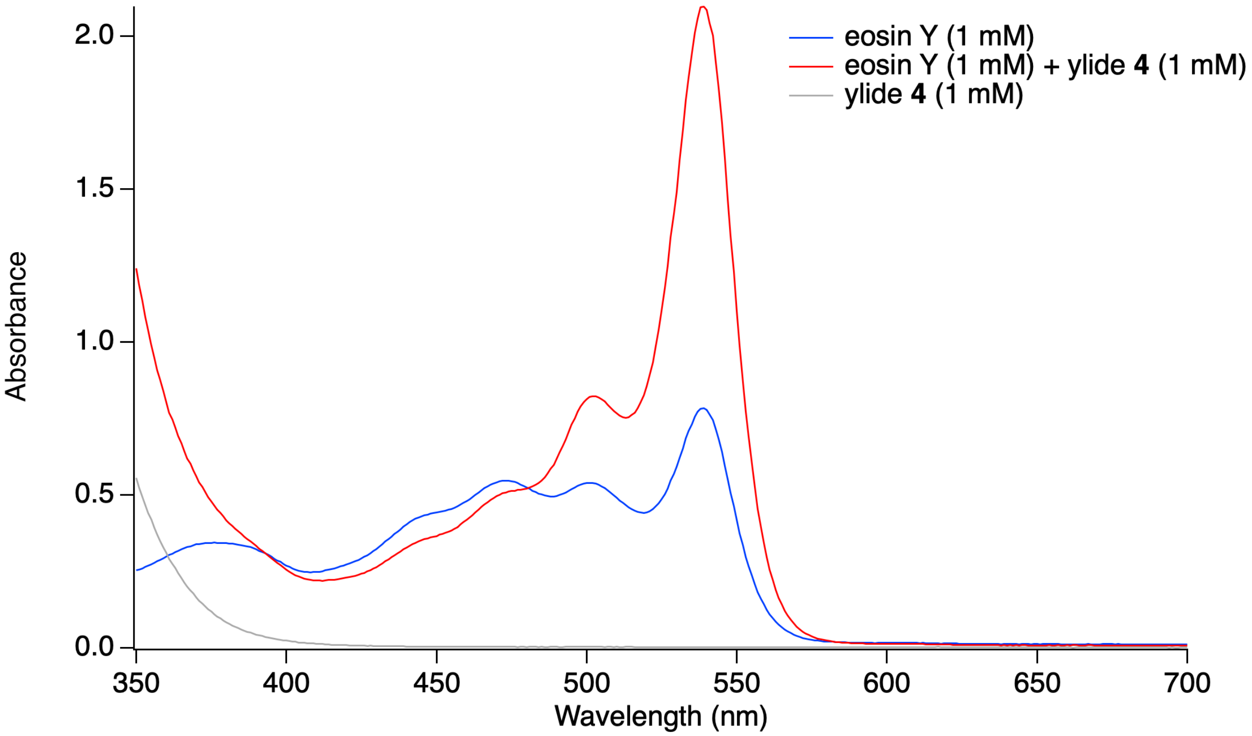

Figure S2. UV–Vis absorption spectra of neutral eosin Y (1 mM), neutral eosin Y (1 mM) with ylide **4** (1 equiv), and ylide **4** (1 mM) in MeCN.

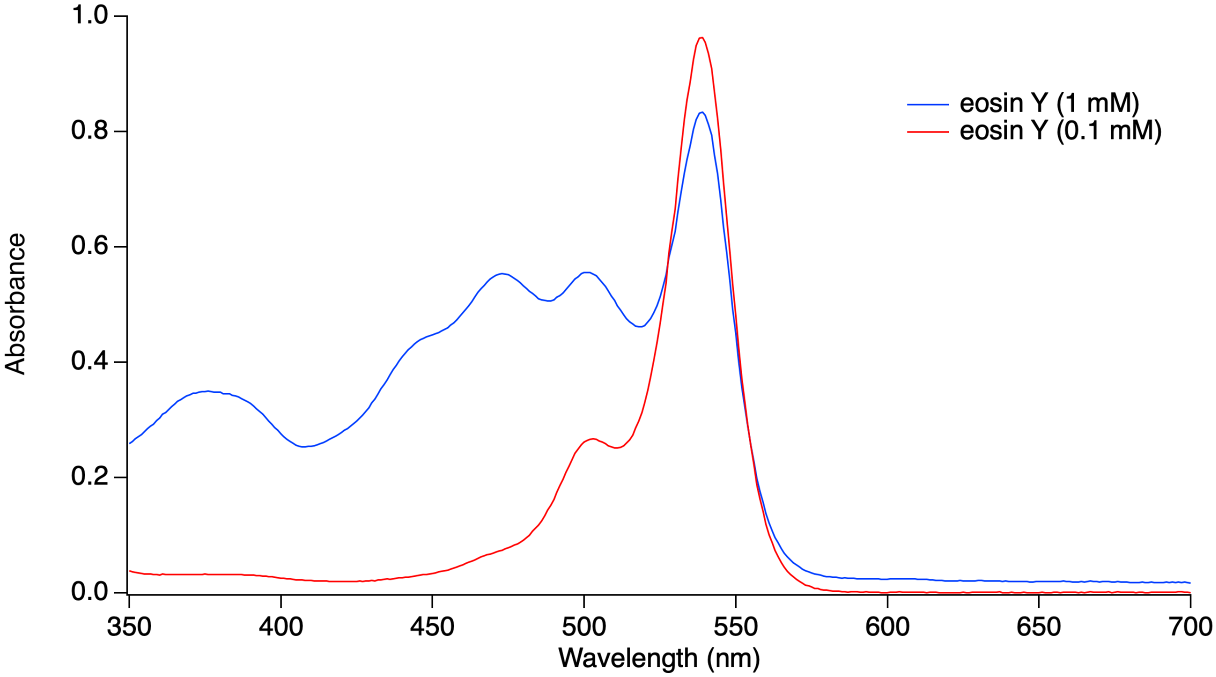

Figure S3. UV–Vis absorption spectra of neutral eosin Y at different concentrations in MeCN.

VI. Analysis of Complexation-Induced Chemical Shifts using MCV

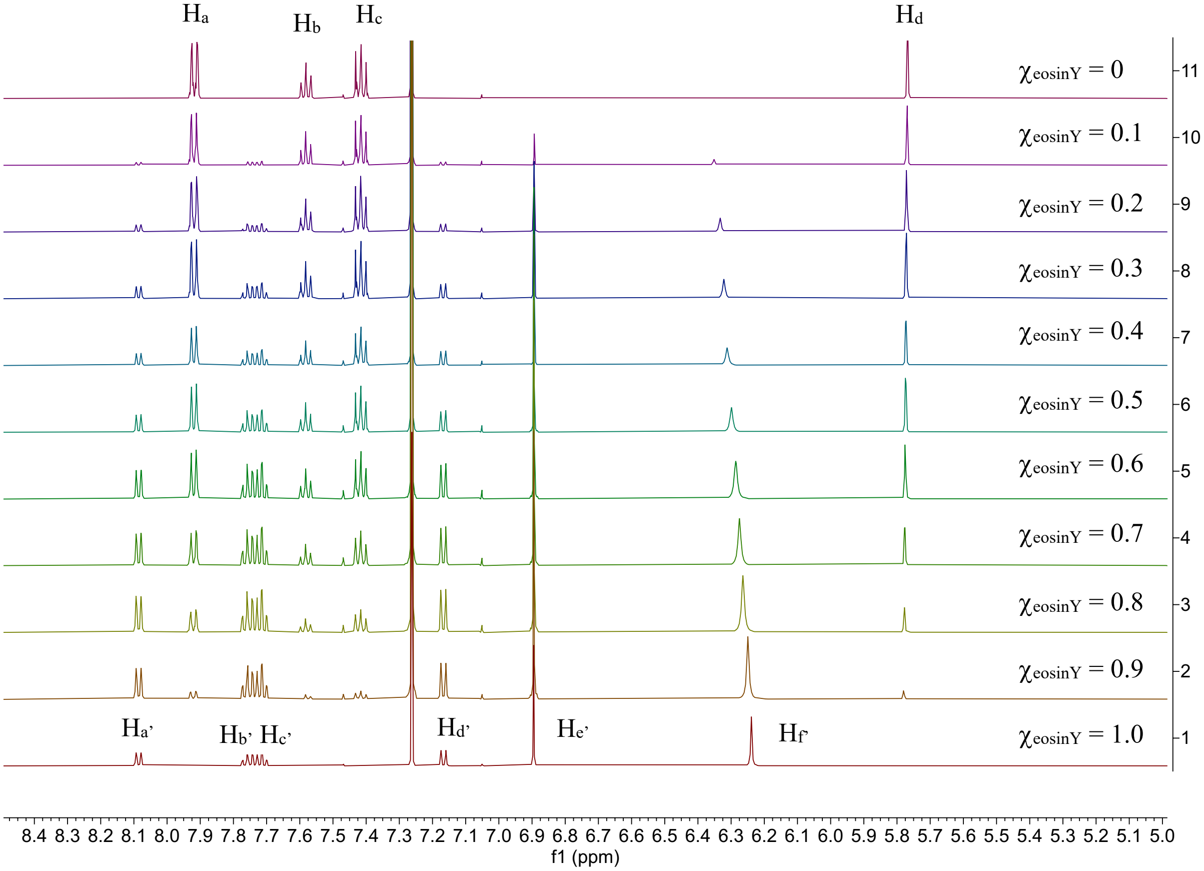

Figure S4. ^{1}H NMR spectra ($CDCl_3$, 500 MHz) of ylide **4** and neutral eosin Y with increasing mole fraction of eosin Y.

Table S1. Chemical Shift of $H_{f'}$ with Increasing Mole Fraction of Eosin Y.

Entry	χ_{eosinY}	δ_{obs} $H_{f'}$ (ppm)	χ_{eosinY} x (δ_{obs} $H_{f'}$ – δ_{free} $H_{f'}$)
1	0	--	0
2	0.1	6.352	0.0113
3	0.2	6.333	0.0188
4	0.3	6.322	0.0249
5	0.4	6.312	0.0292
6	0.5	6.299	0.0300
7	0.6	6.286	0.0282
8	0.7	6.275	0.0252
9	0.8	6.264	0.0200
10	0.9	6.249	0.0090
11	1.0	6.239	0

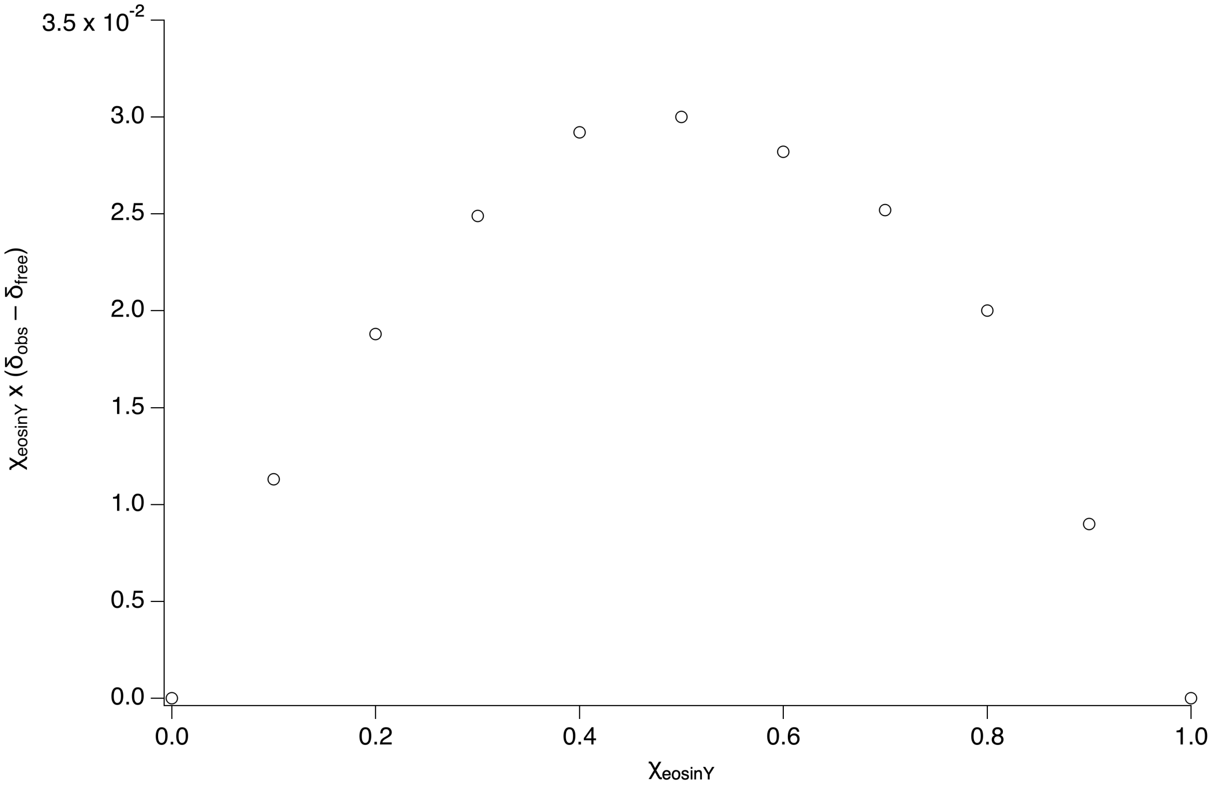

Figure S5. Job plot for mixtures of neutral eosin Y and iodonium ylide **4** in $CDCl_3$.

VII. Transient Absorption Spectra

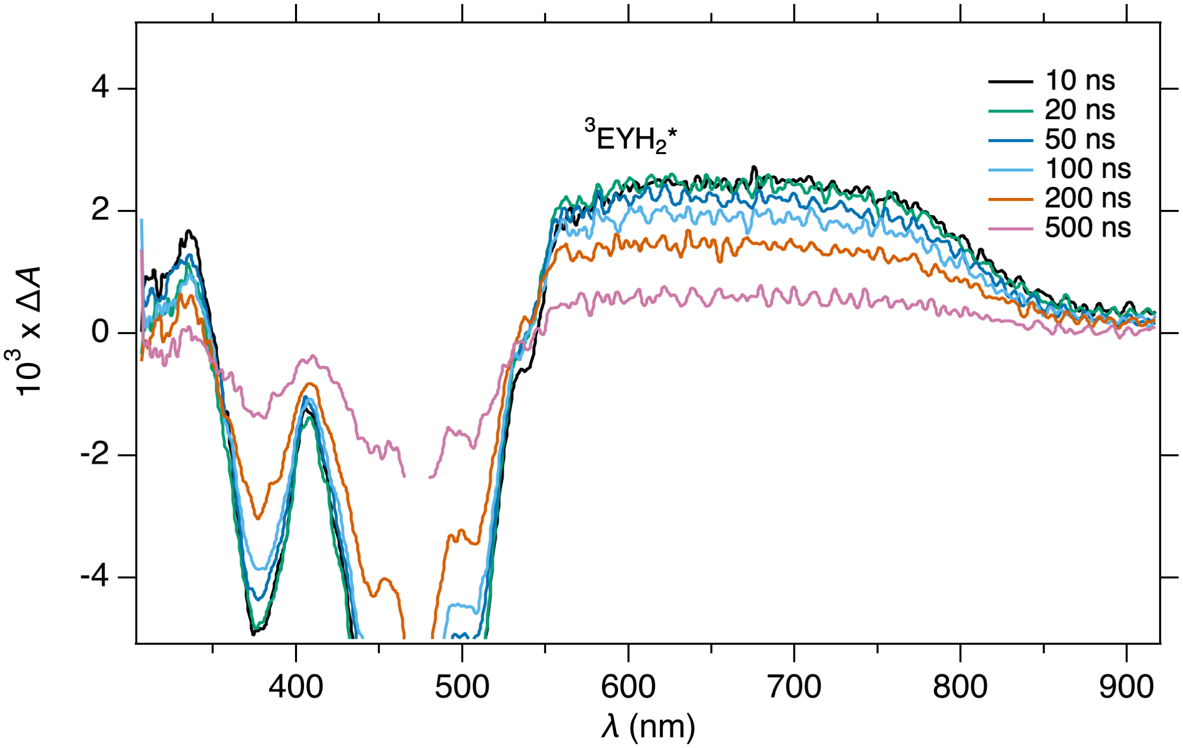

Figure S6. Transient absorption spectra of EYH_2 (1 mM) in nondegassed MeCN at different times after excitation (excitation wavelength 470 nm).

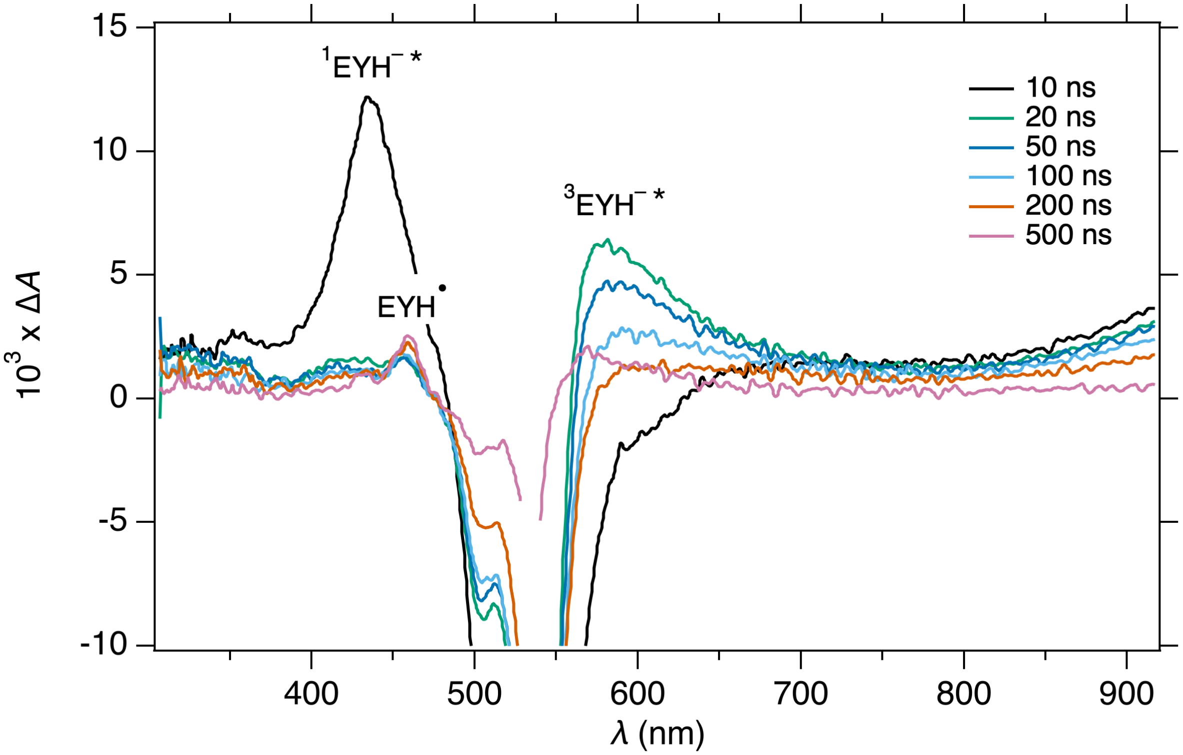

Figure S7. Transient absorption spectra of EYH_2 (1 mM) and iodonium ylide **4** (1 mM) in nondegassed MeCN at different times after excitation (excitation wavelength 532 nm).

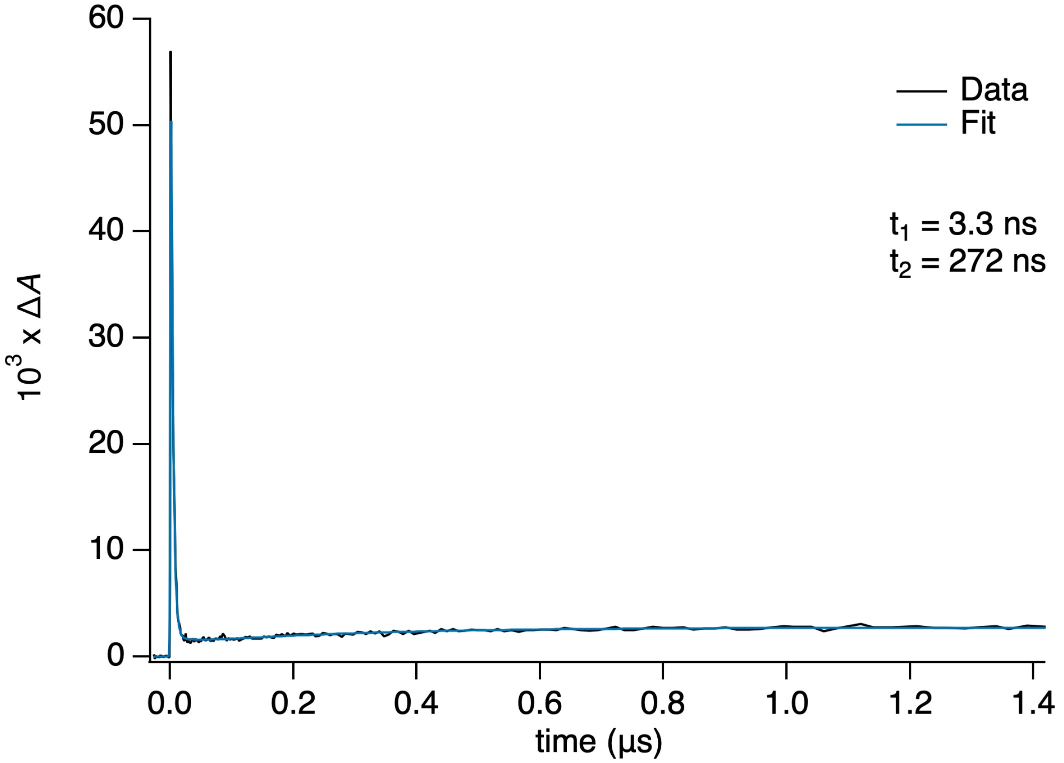

Figure S8. Kinetic trace of EYH_2 (1 mM) and iodonium ylide **4** (1 mM) in nondegassed MeCN and the corresponding fit at 460 nm.

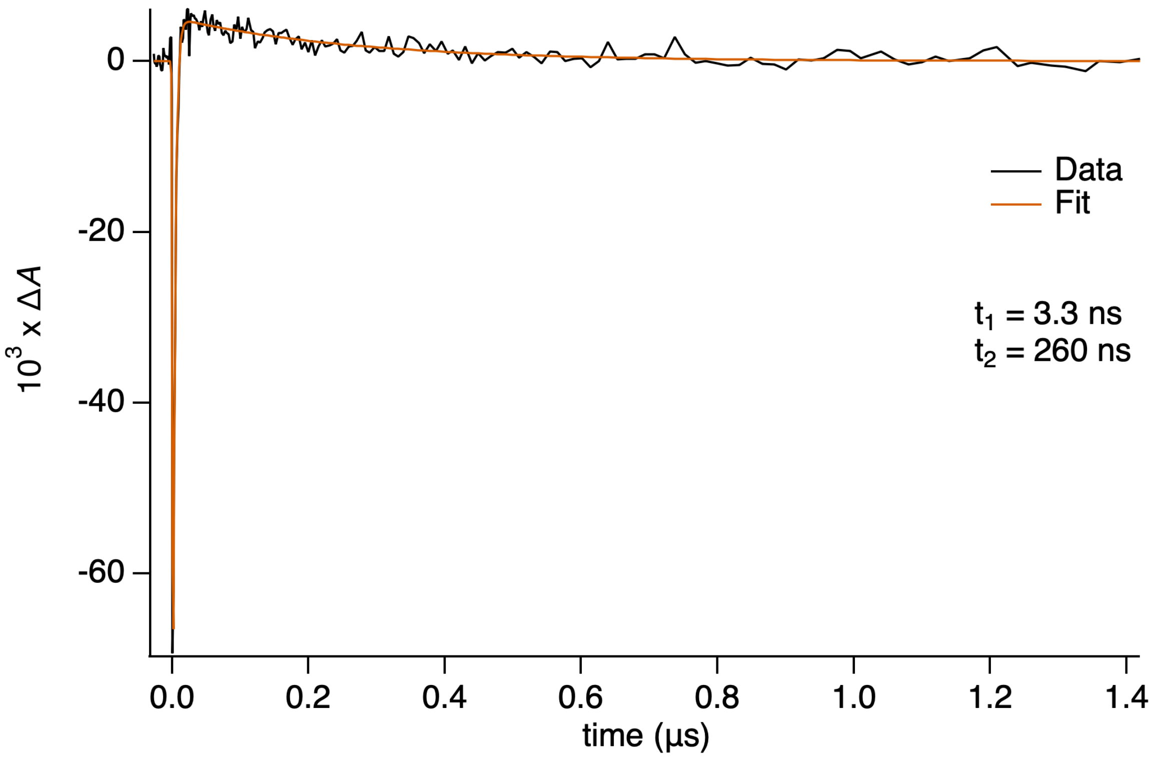

Figure S9. Kinetic trace of EYH_2 (1 mM) and iodonium ylide **4** (1 mM) in nondegassed MeCN and the corresponding fit at 588 nm.

VIII. References

(1) Schank, K.; Lick, C. Ozonolytic Fragmentation of Phenyliodonium β-Diketonates; A Convenient Synthesis of Unsolvated Vic-Triketones. *Synthesis* **1983**, *1983* (05), 392–395. DOI: 10.1055/s-1983-30350.

(2) Liang, M.; He, M.; Zhong, Z.; Wan, B.; Du, Q.; Mai, S. Catalytic and Base-free Suzuki-type α-Arylation of Cyclic 1,3-Dicarbonyls via a Cyclic Iodonium Ylide Strategy. *Angew. Chem., Int. Ed.* **2024**, *63* (17), e202400741. DOI: 10.1002/anie.202400741.

(3) Chidley, T.; Jameel, I.; Rizwan, S.; Peixoto, P. A.; Pouységu, L.; Quideau, S.; Hopkins, W. S.; Murphy, G. K. Blue LED Irradiation of Iodonium Ylides Gives Diradical Intermediates for Efficient Metal-free Cyclopropanation with Alkenes. *Angew. Chem., Int. Ed.* **2019**, *58* (47), 16959–16965. DOI: 10.1002/anie.201908994.

(4) Zhu, C.; Yoshimura, A.; Solntsev, P.; Ji, L.; Wei, Y.; Nemykin, V. N.; Zhdankin, V. V. New Highly Soluble Dimedone-Derived Iodonium Ylides: Preparation, X-Ray Structure, and Reaction with Carbodiimide Leading to Oxazole Derivatives. *Chem. Commun.***2012**, *48* (81), 10108. DOI: 10.1039/c2cc35708a.

(5) Goudreau, S. R.; Marcoux, D.; Charette, A. B. General Method for the Synthesis of Phenyliodonium Ylides from Malonate Esters: Easy Access to 1,1-Cyclopropane Diesters. *J. Org. Chem.* **2009**, *74* (1), 470–473. DOI: 10.1021/jo802208q.

(6) Vucicevic, J.; Srdic-Rajic, T.; Pieroni, M.; Laurila, J. M. M.; Perovic, V.; Tassini, S.; Azzali, E.; Costantino, G.; Glisic, S.; Agbaba, D.; Scheinin, M.; Nikolic, K.; Radi, M.; Veljkovic, N. A Combined Ligand- and Structure-Based Approach for the Identification of Rilmenidine-Derived Compounds Which Synergize the Antitumor Effects of Doxorubicin. *Bioorg. Med. Chem.* **2016**, *24* (14), 3174–3183. DOI: 10.1016/j.bmc.2016.05.043.

(7) Kobayashi, K.; Sakashita, K.; Akamatsu, H.; Tanaka, K.; Uchida, M.; Uneda, T.; Kitamura, T.; Morikawa, O.; Konishi, H. CAN-Mediated Formation of Furopyranones and Furoquinolinones. *Heterocycles* **1999**, *51* (12), 2881–2905. DOI: 10.3987/COM-99-8688.

(8) Appendino, G.; Cravotto, G.; Palmisano, G.; Annunziata, R. Oxidative Addition of 4-Hydroxycoumarin to Alkenes. An Expeditious Entry to 2,3-Dihydro-4H-furo-[3,2*c*][1]benzopyran-4-ones[1]. *Synth. Commun.* **1996**, *26* (18), 3359–3371. DOI: 10.1080/00397919608003739.

(9) Kalpogiannaki, D.; Martini, C.-I.; Nikopoulou, A.; Nyxas, J. A.; Pantazi, V.; Hadjiarapoglou, L. P. Fused Dihydrofurans from the One-Pot, Three-Component Reaction of 1,3-Cyclohexanedione, Iodobenzene Diacetate and Alkenes. *Tetrahedron* **2013**, *69* (5), 1566–1575. DOI: 10.1016/j.tet.2012.12.006.

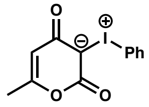

4

^{1}H NMR
500 MHz, DMSO-d_6

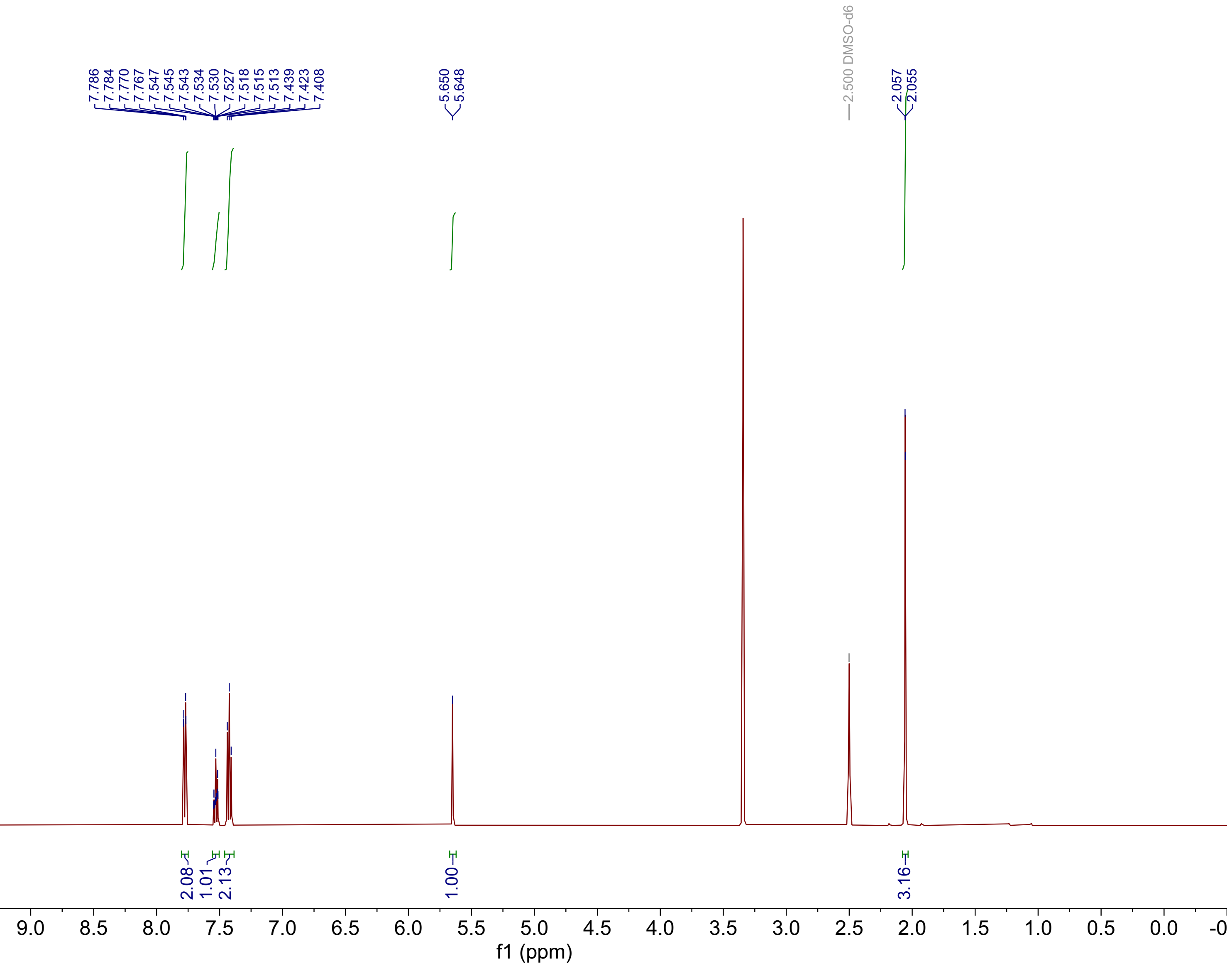

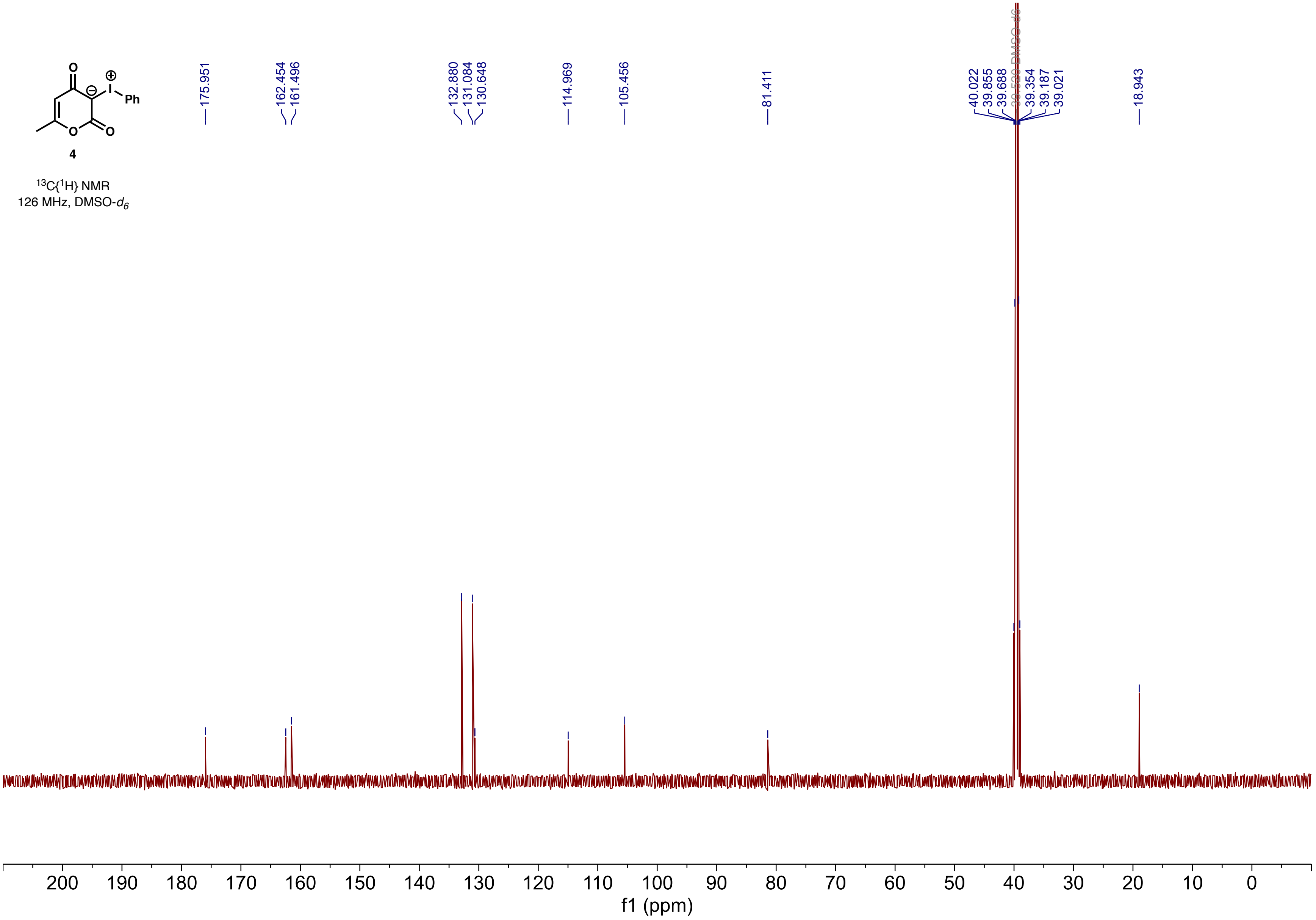

4
$^{13}C\{^{1}H\}$ NMR
126 MHz, DMSO-d_6
175.951
162.454
161.496
132.880
131.084
130.648
114.969
105.456
81.411
40.022
39.855
39.688
DMSO-d6
39.354
39.187
39.021
18.943
200 190 180 170 160 150 140 130 120 110 100 90 80 70 60 50 40 30 20 10 0
f1 (ppm)

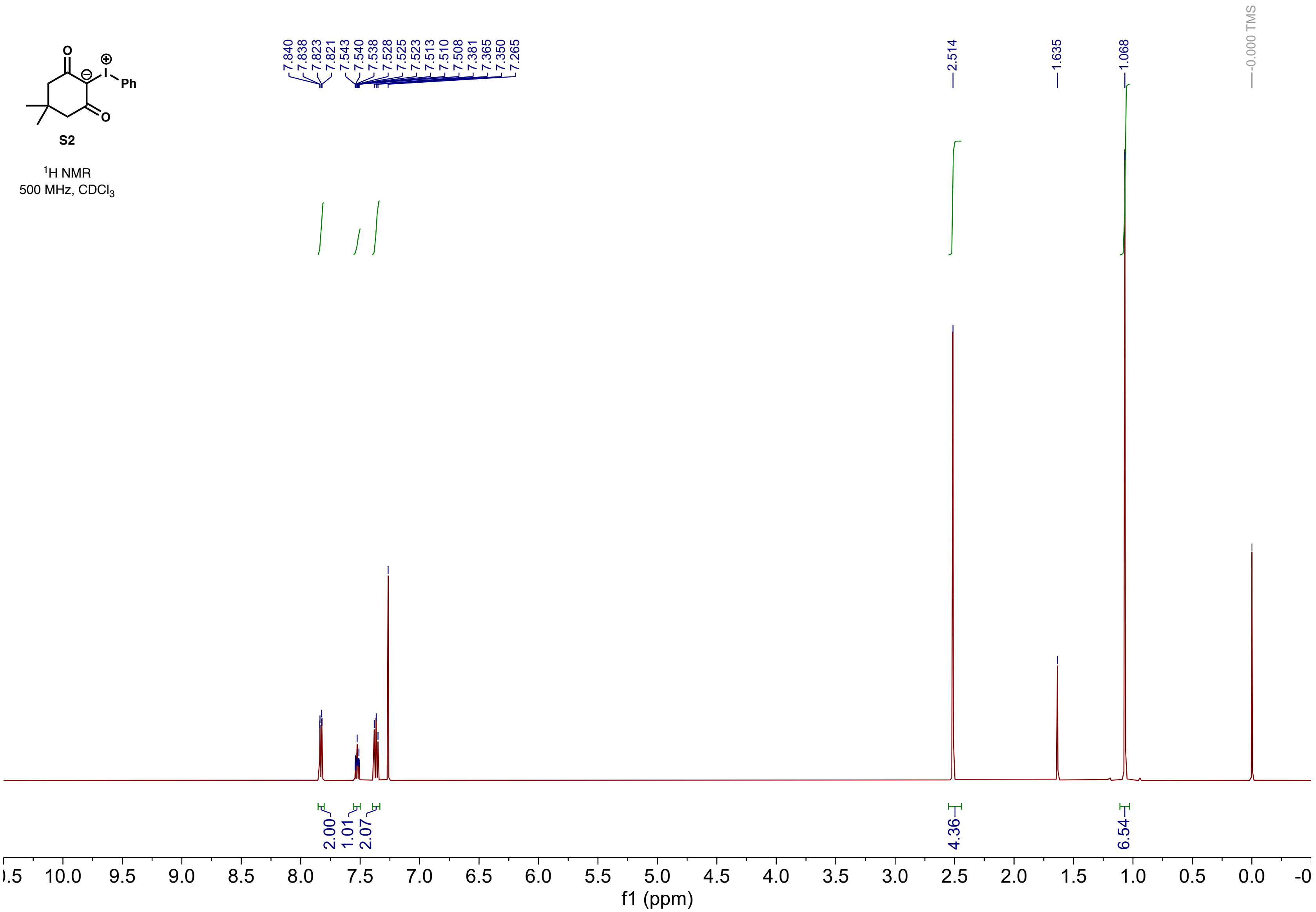
S2
¹H NMR
500 MHz, CDCl3
7.840
7.838
7.823
7.821
7.543
7.540
7.538
7.528
7.525
7.523
7.513
7.510
7.508
7.381
7.365
7.350
7.265
2.514
1.635
1.068
0.000 TMS
2.00
1.01
2.07
4.36
6.54
f1 (ppm)

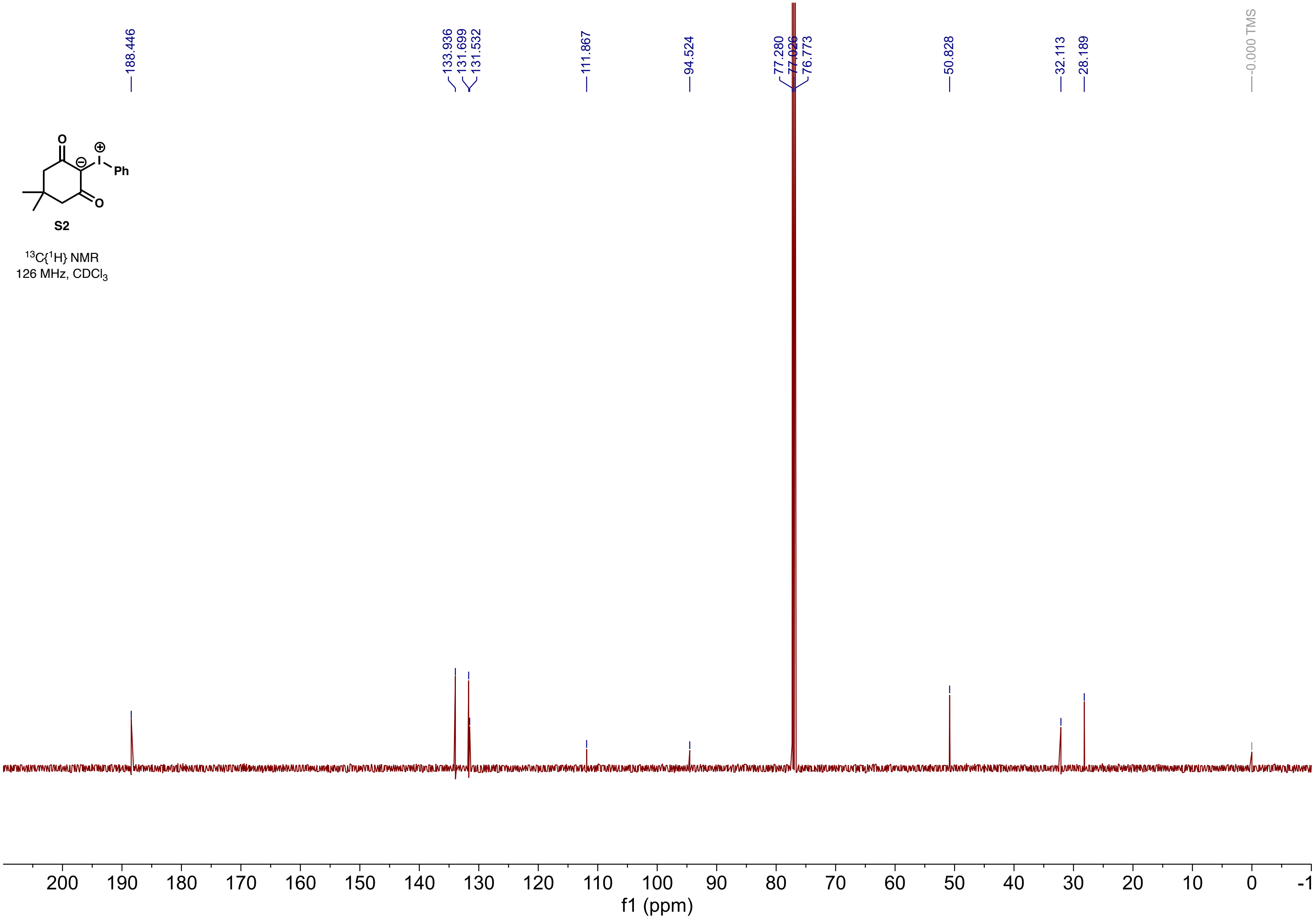

188.446
133.936
131.699
131.532
111.867
94.524
77.280
77.026
76.773
50.828
32.113
28.189
0.000 TMS
O
I
Ph
O
S2
^{13}C{^{1}H} NMR
126 MHz, CDCl$_3$
200
190
180
170
160
150
140
130
120
110
100
90
80
70
60
50
40
30
20
10
0
-1
f1 (ppm)

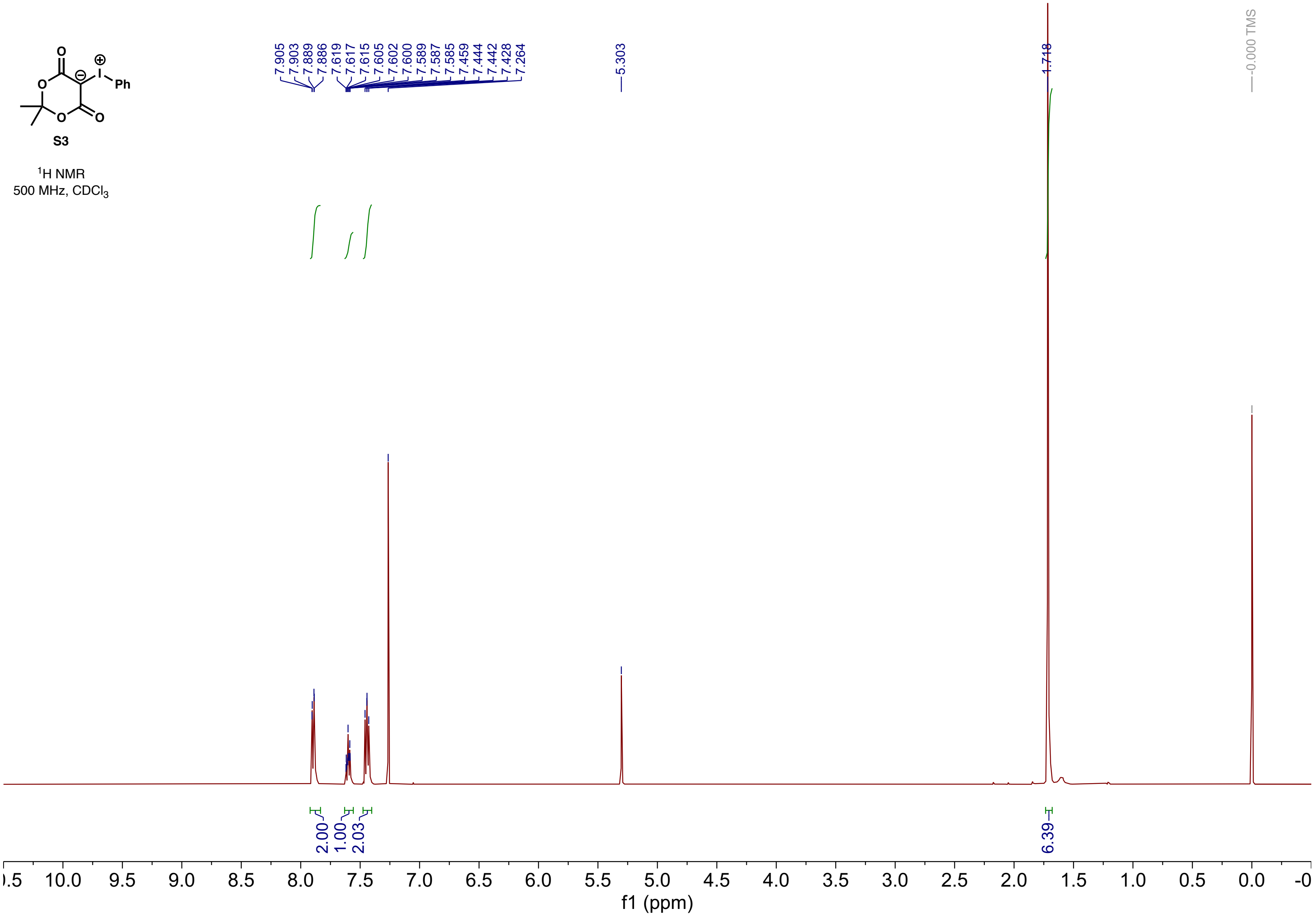
S3
1H NMR
500 MHz, CDCl3
7.905
7.903
7.889
7.886
7.619
7.617
7.615
7.605
7.602
7.600
7.589
7.587
7.585
7.459
7.444
7.442
7.428
7.264
5.303
1.718
-0.000 TMS
2.00
1.00
2.03
6.39
f1 (ppm)

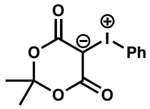

S3

$^{13}C\{^1H\}$ NMR
126 MHz, $CDCl_3$

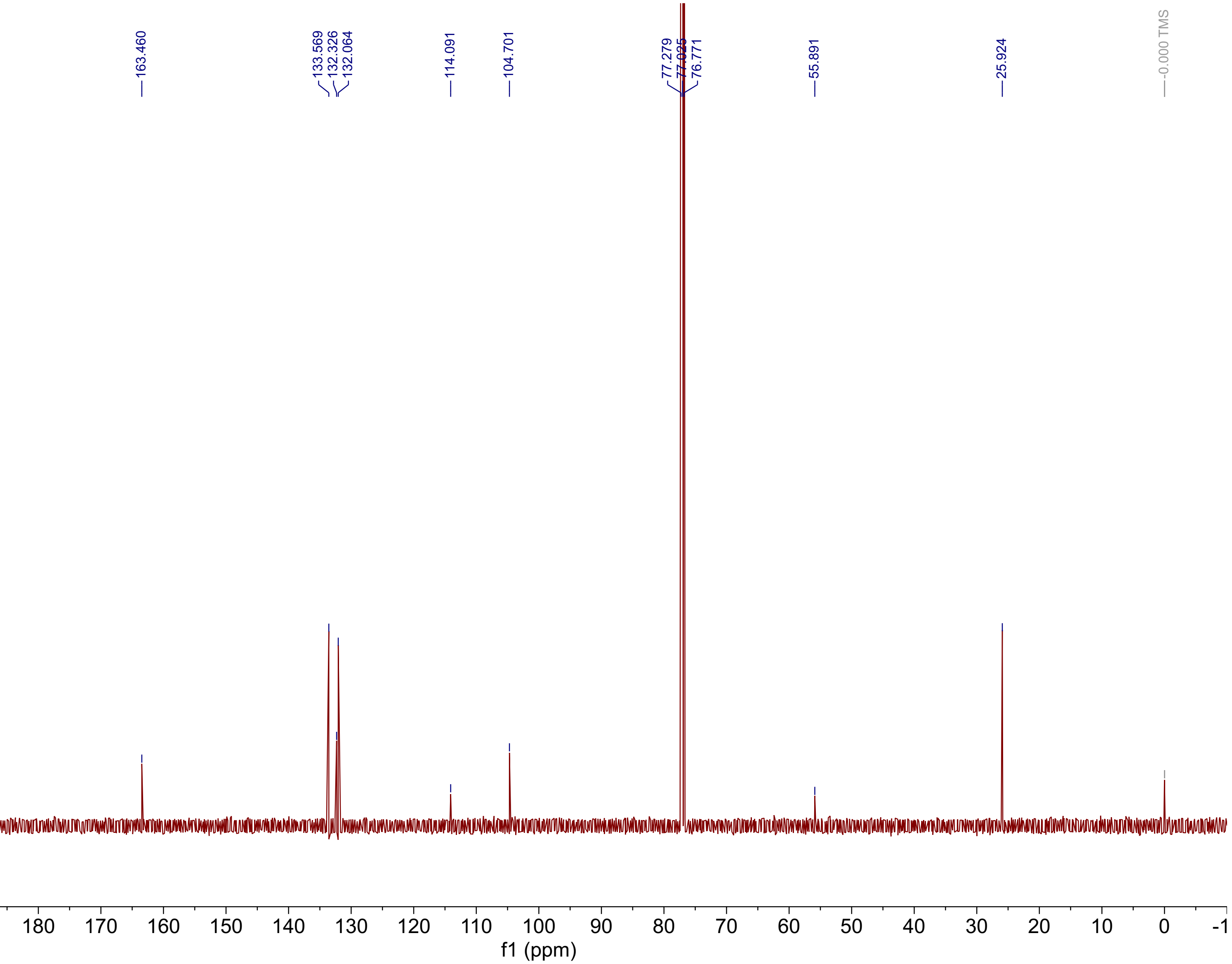

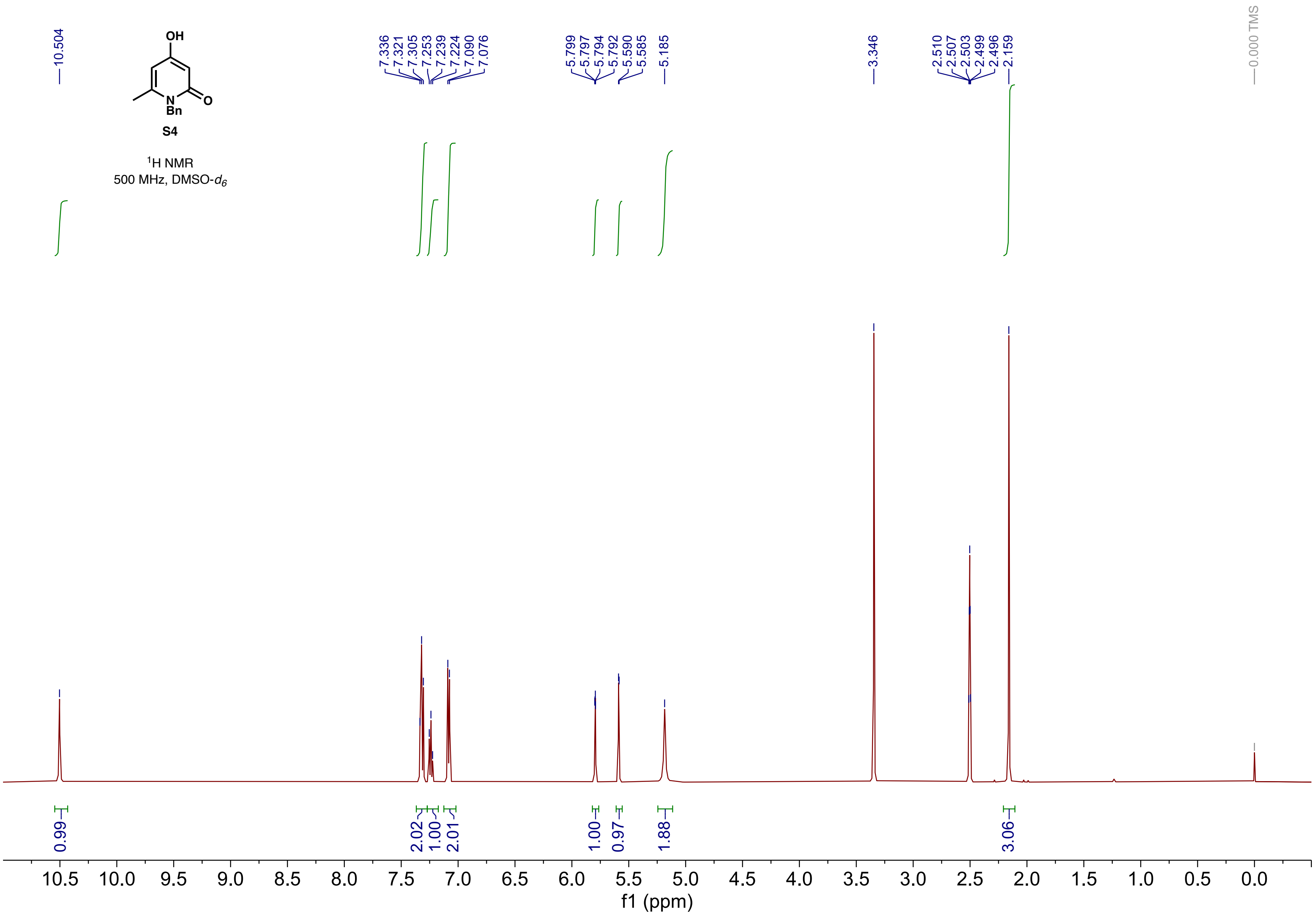

OH
N
Bn
O
S4
¹H NMR
500 MHz, DMSO-d6
10.504
7.336
7.321
7.305
7.253
7.239
7.224
7.090
7.076
5.799
5.797
5.794
5.792
5.590
5.585
5.185
3.346
2.510
2.507
2.503
2.499
2.496
2.159
0.000 TMS
0.99
2.02
1.00
2.01
1.00
0.97
1.88
3.06
f1 (ppm)

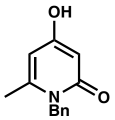

S4

$^{13}C\{^1H\}$ NMR
126 MHz, DMSO-d_6

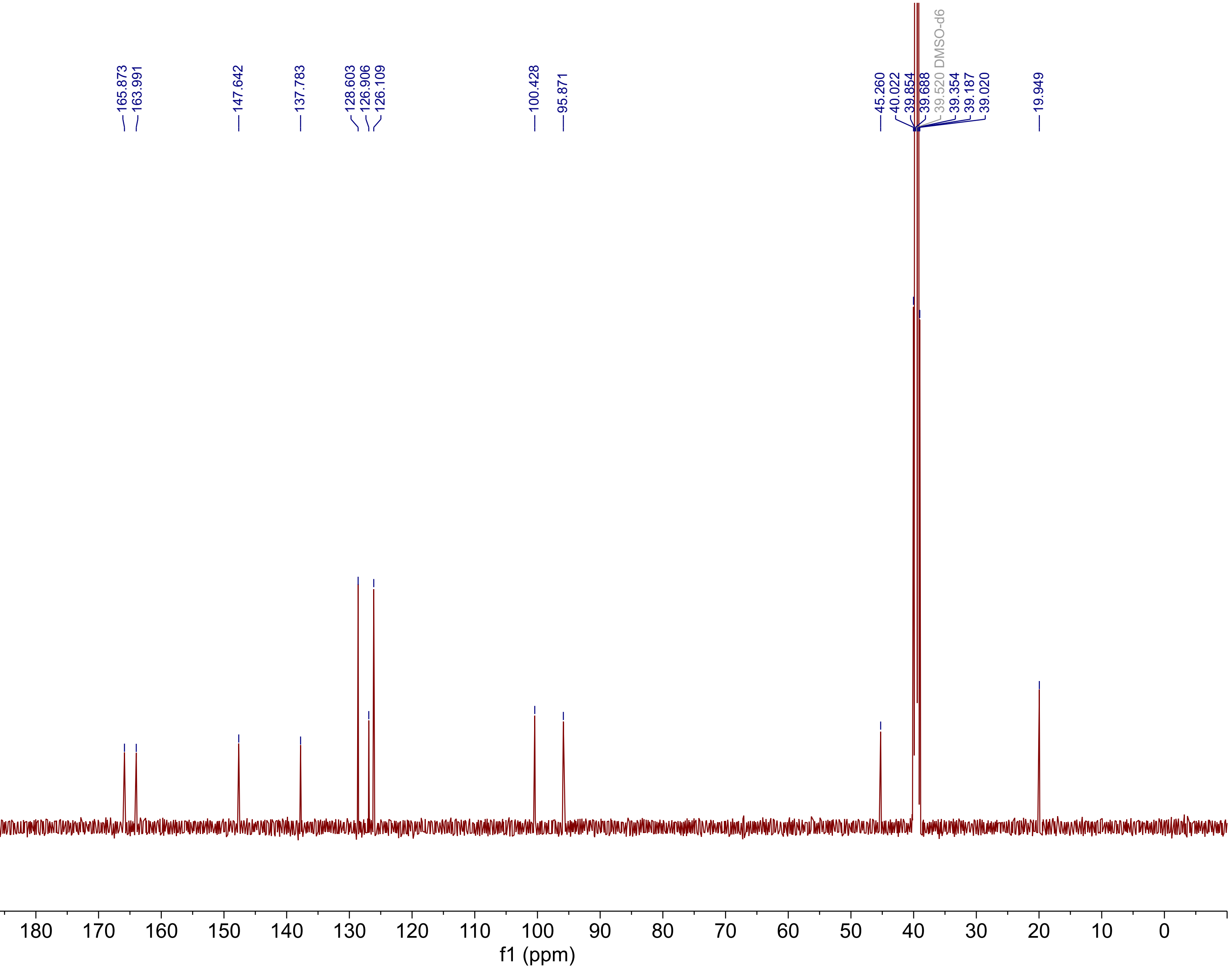

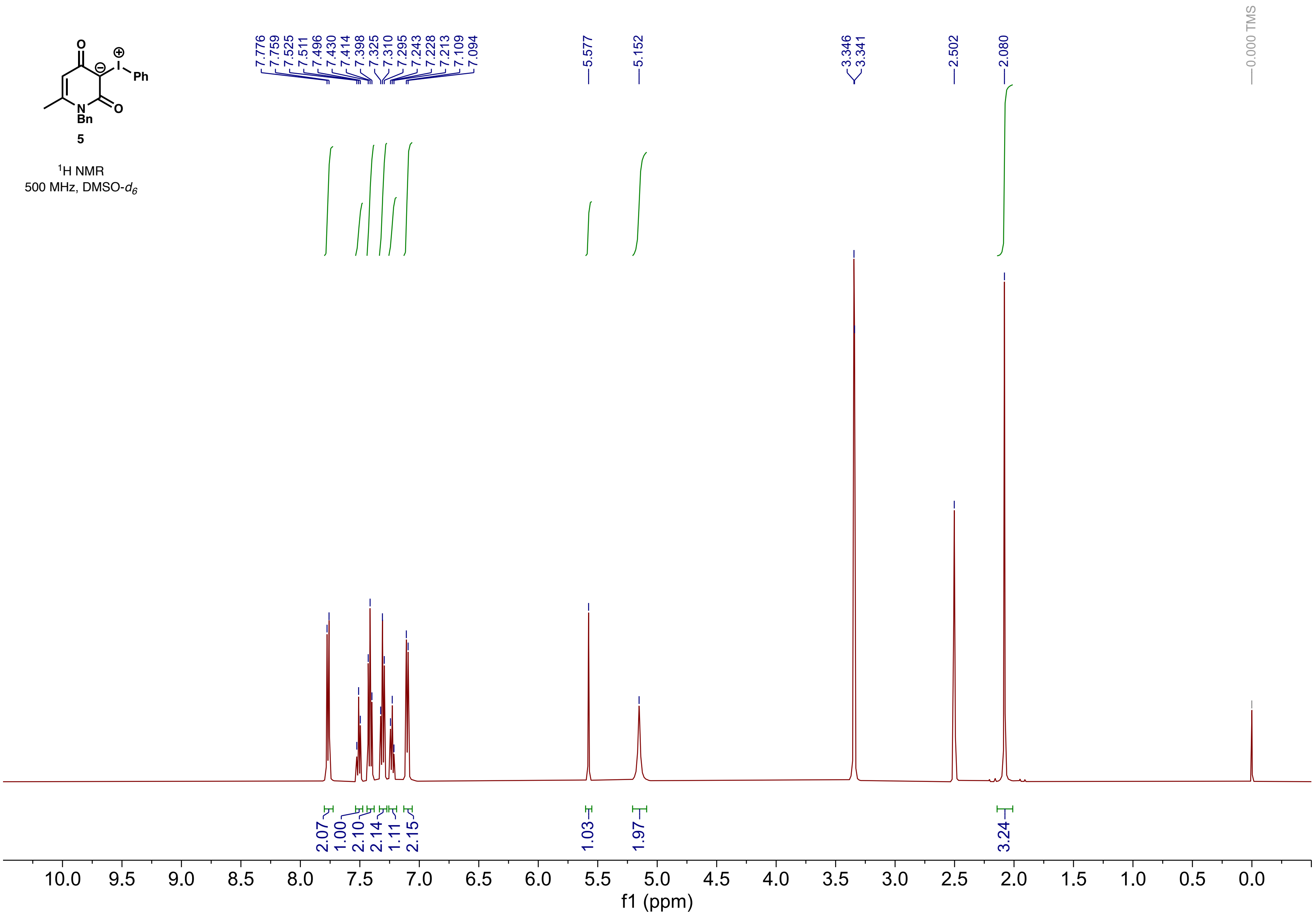
5
^{1}H NMR
500 MHz, DMSO-d_6
7.776
7.759
7.525
7.511
7.496
7.430
7.414
7.398
7.325
7.310
7.295
7.243
7.228
7.213
7.109
7.094
5.577
5.152
3.346
3.341
2.502
2.080
0.000 TMS
2.07
1.00
2.10
2.14
1.11
2.15
1.03
1.97
3.24
f1 (ppm)

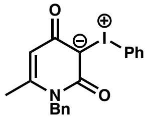

5

$^{13}C\{^{1}H\}$ NMR
126 MHz, DMSO-d_6

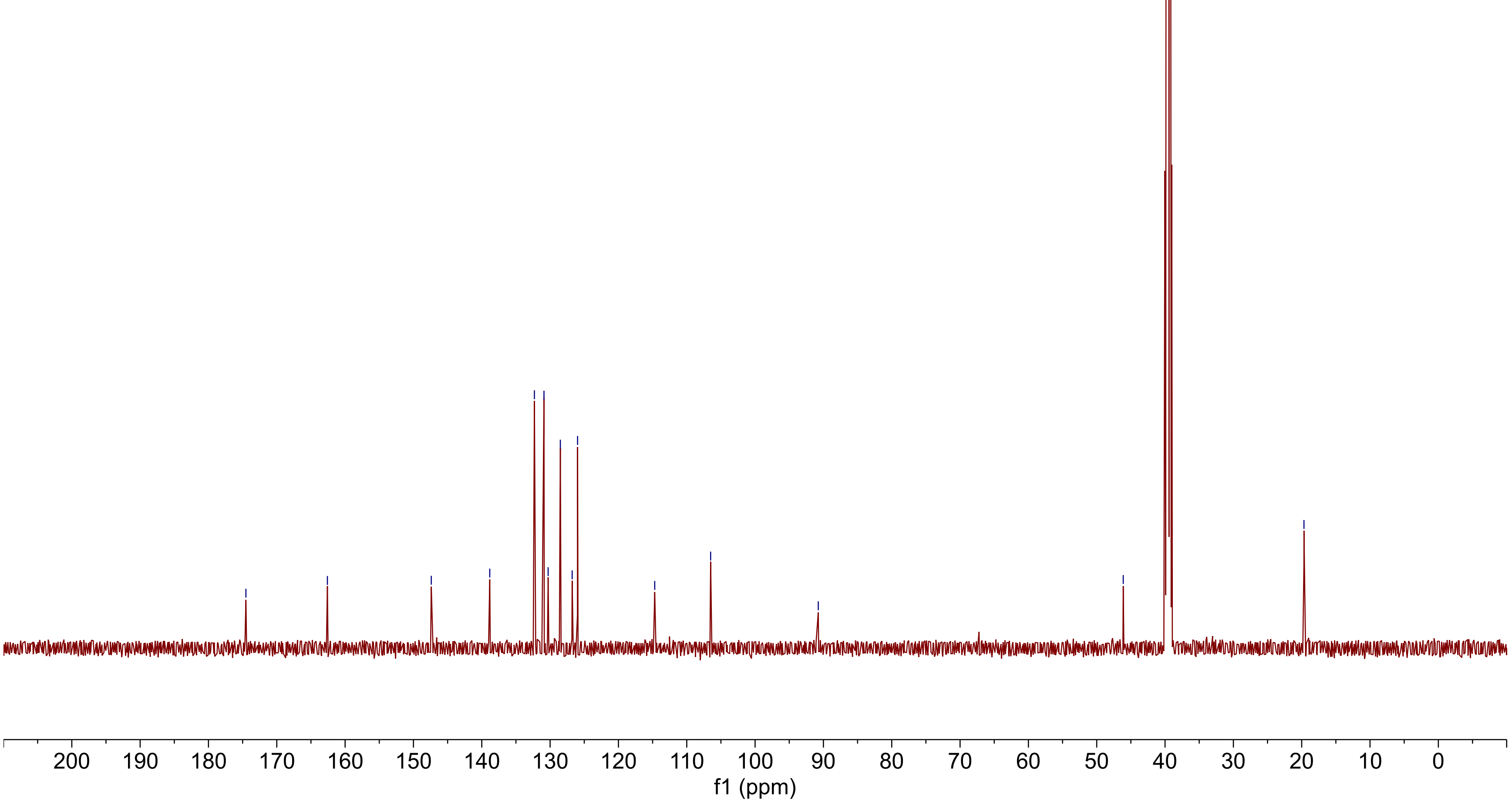

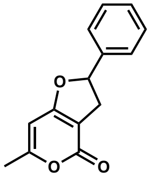

6

^{1}H NMR
500 MHz, $CDCl_3$

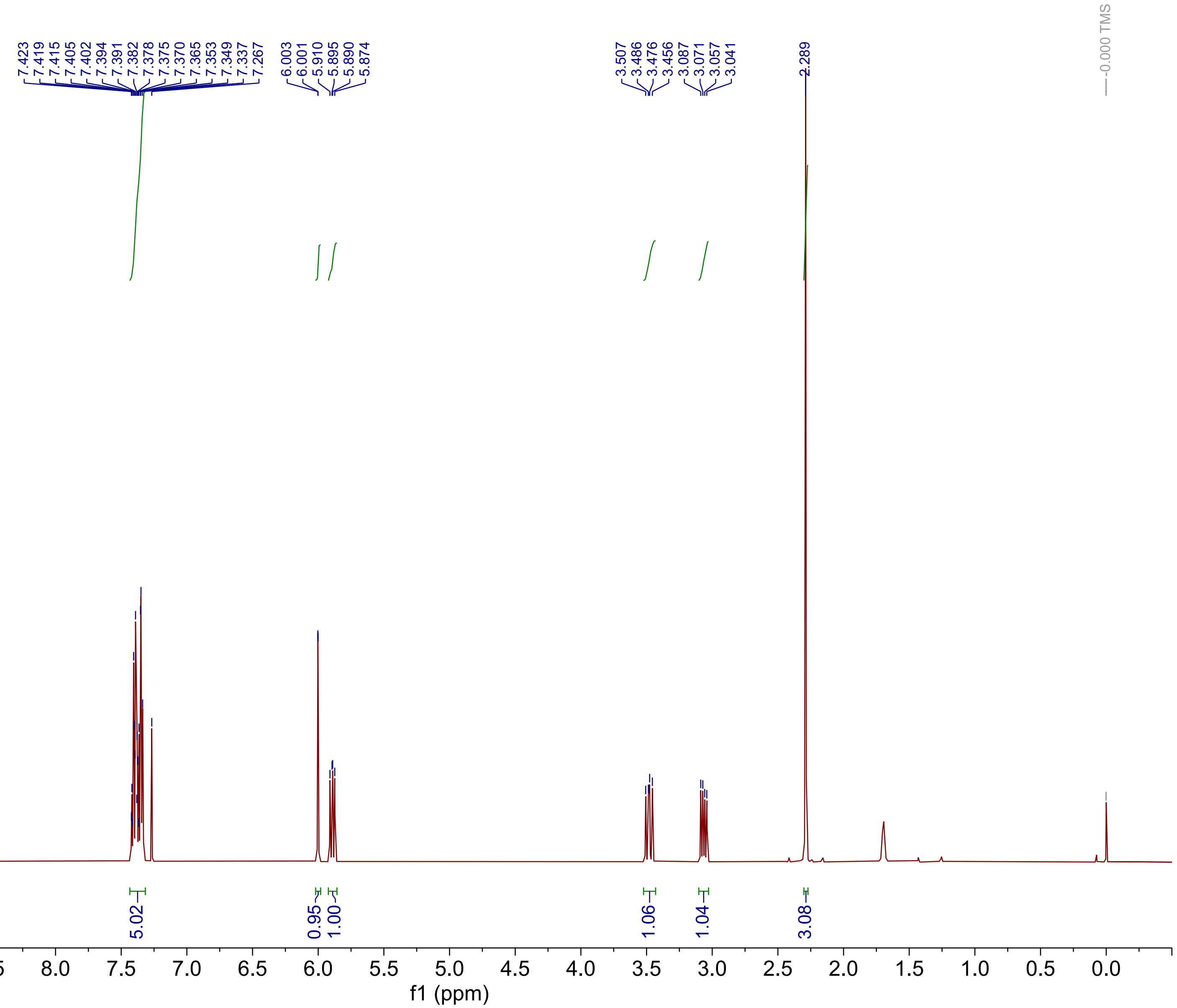

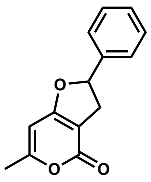

6

$^{13}C\{^1H\}$ NMR
126 MHz, $CDCl_3$

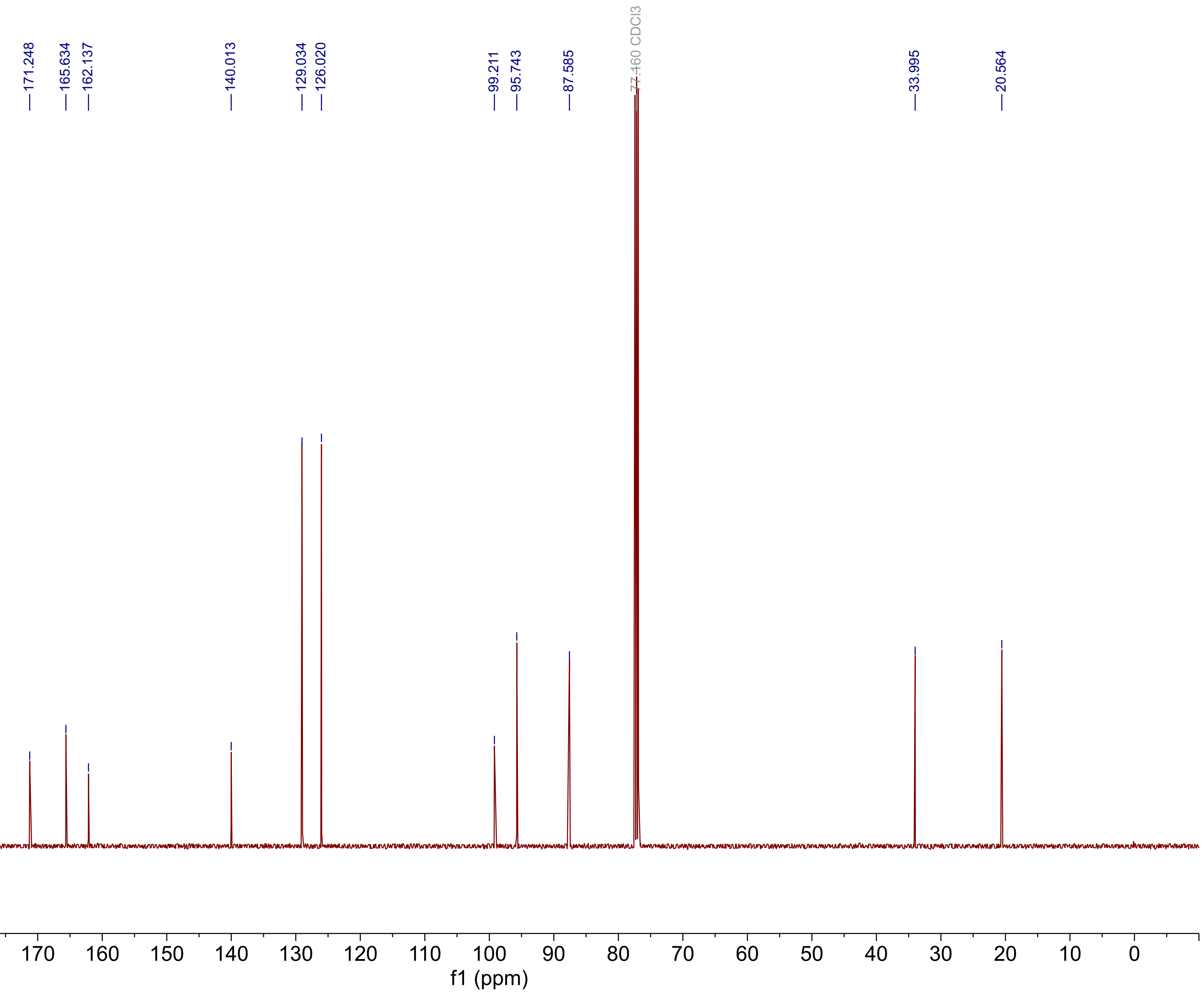

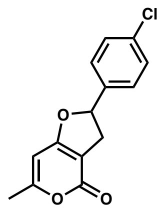

9

^1H NMR
500 MHz, $CDCl_3$

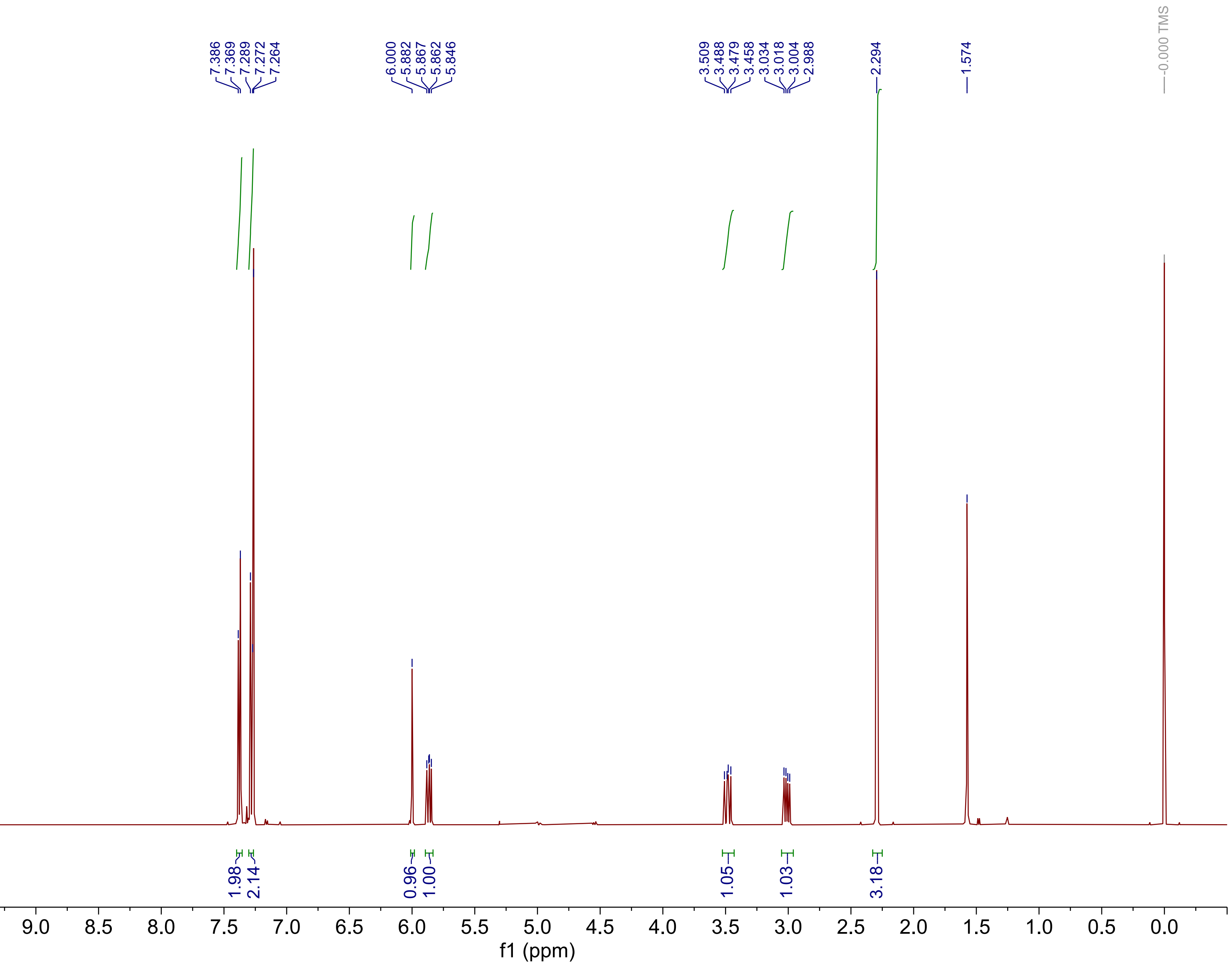

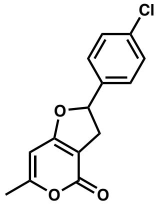

9

$^{13}C\{^{1}H\}$ NMR
126 MHz, $CDCl_3$

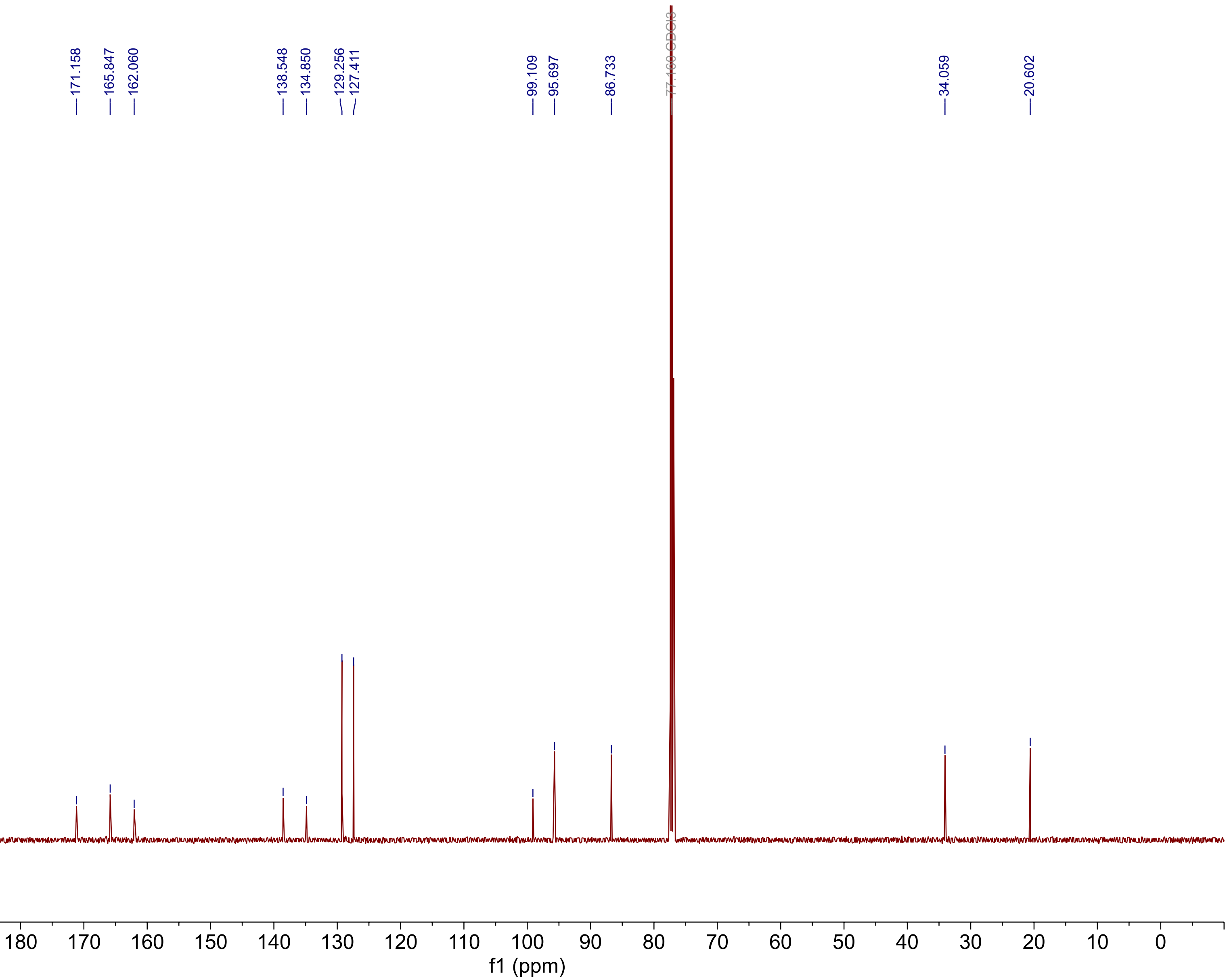

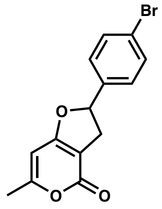

10

^1H NMR
500 MHz, $CDCl_3$

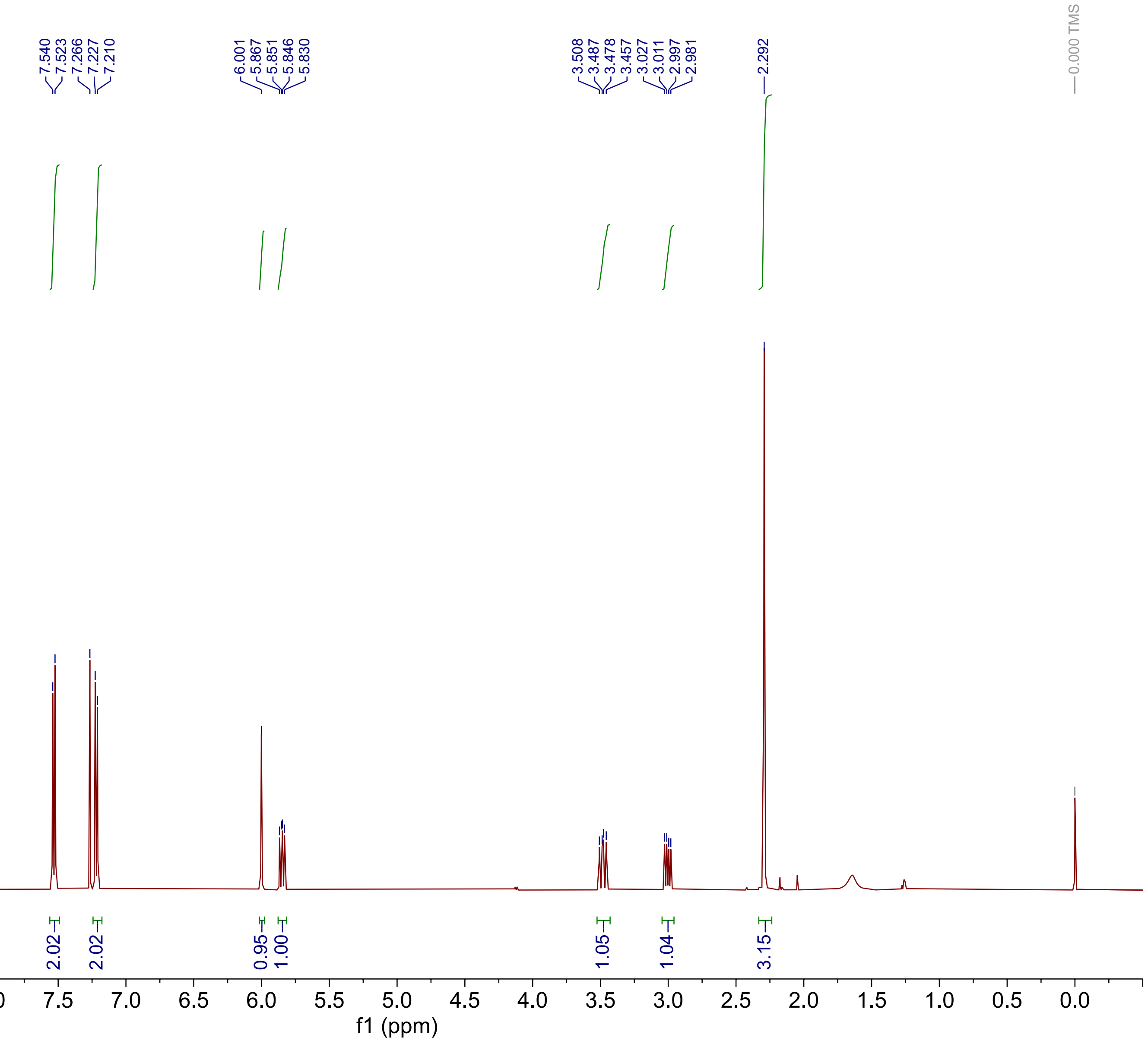

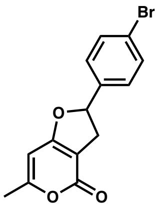

10

$^{13}C\{^{1}H\}$ NMR
126 MHz, $CDCl_3$

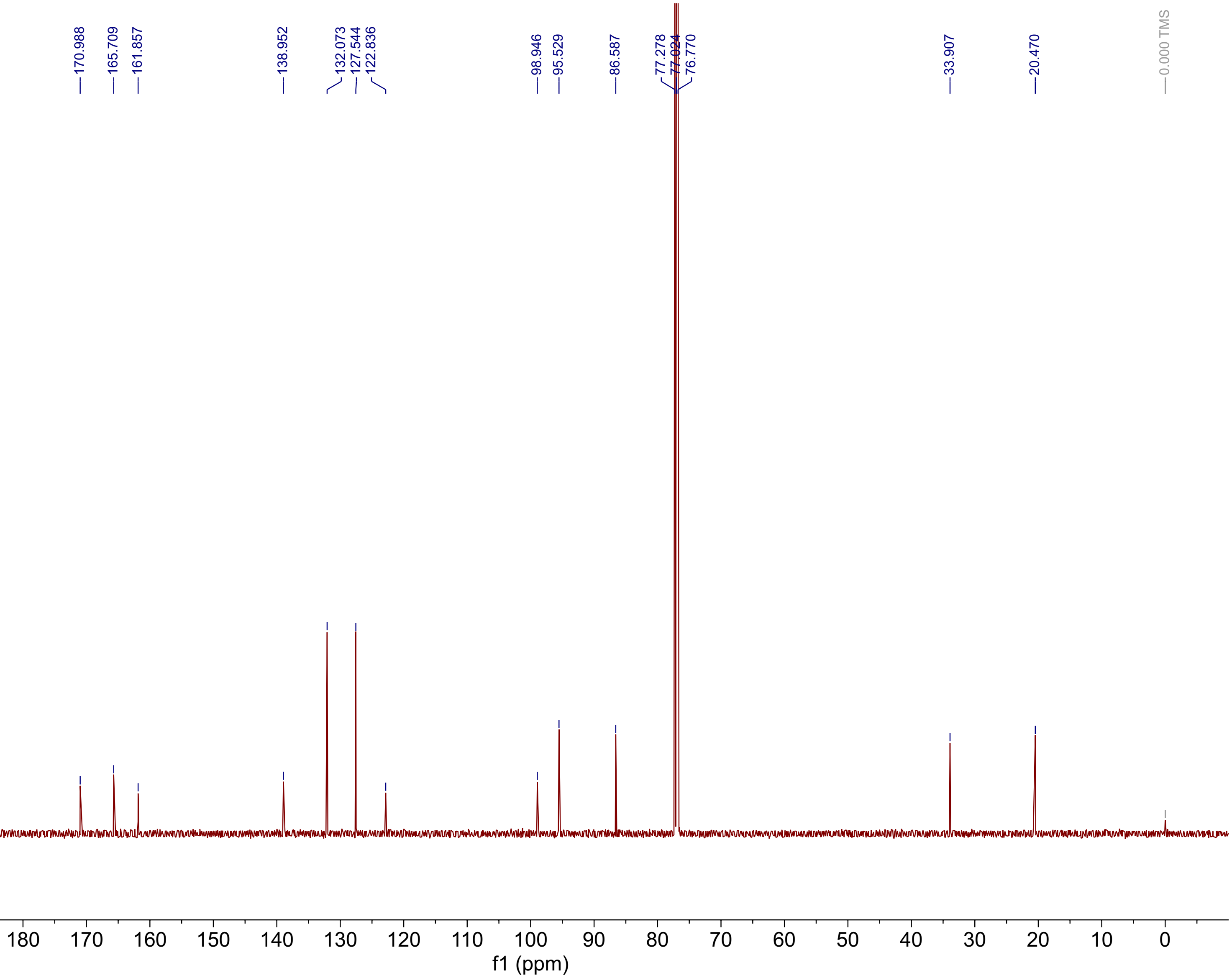

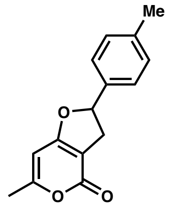

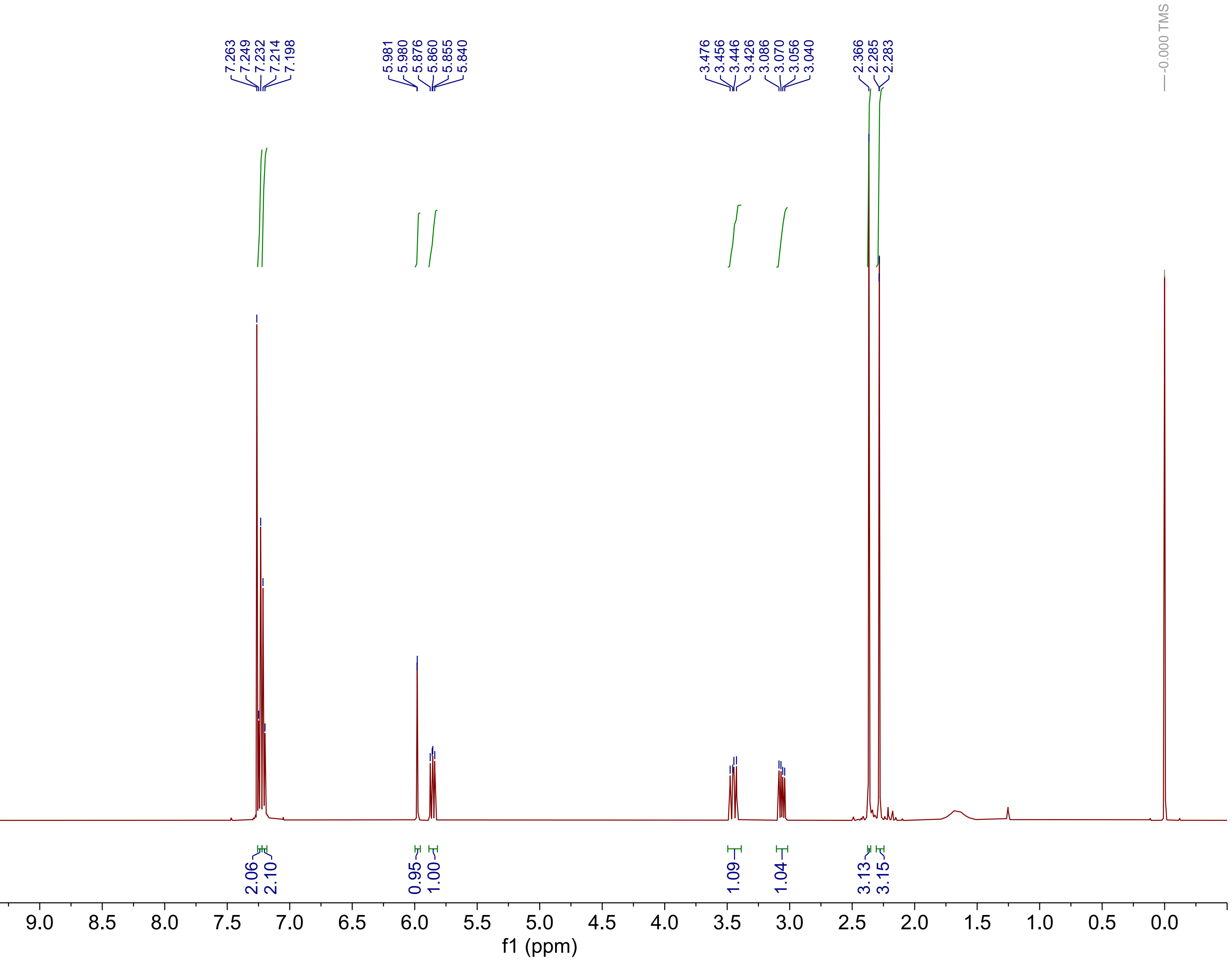

11

^{1}H NMR
500 MHz, $CDCl_3$

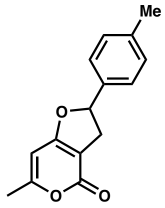

11

$^{13}C\{^{1}H\}$ NMR
126 MHz, $CDCl_3$

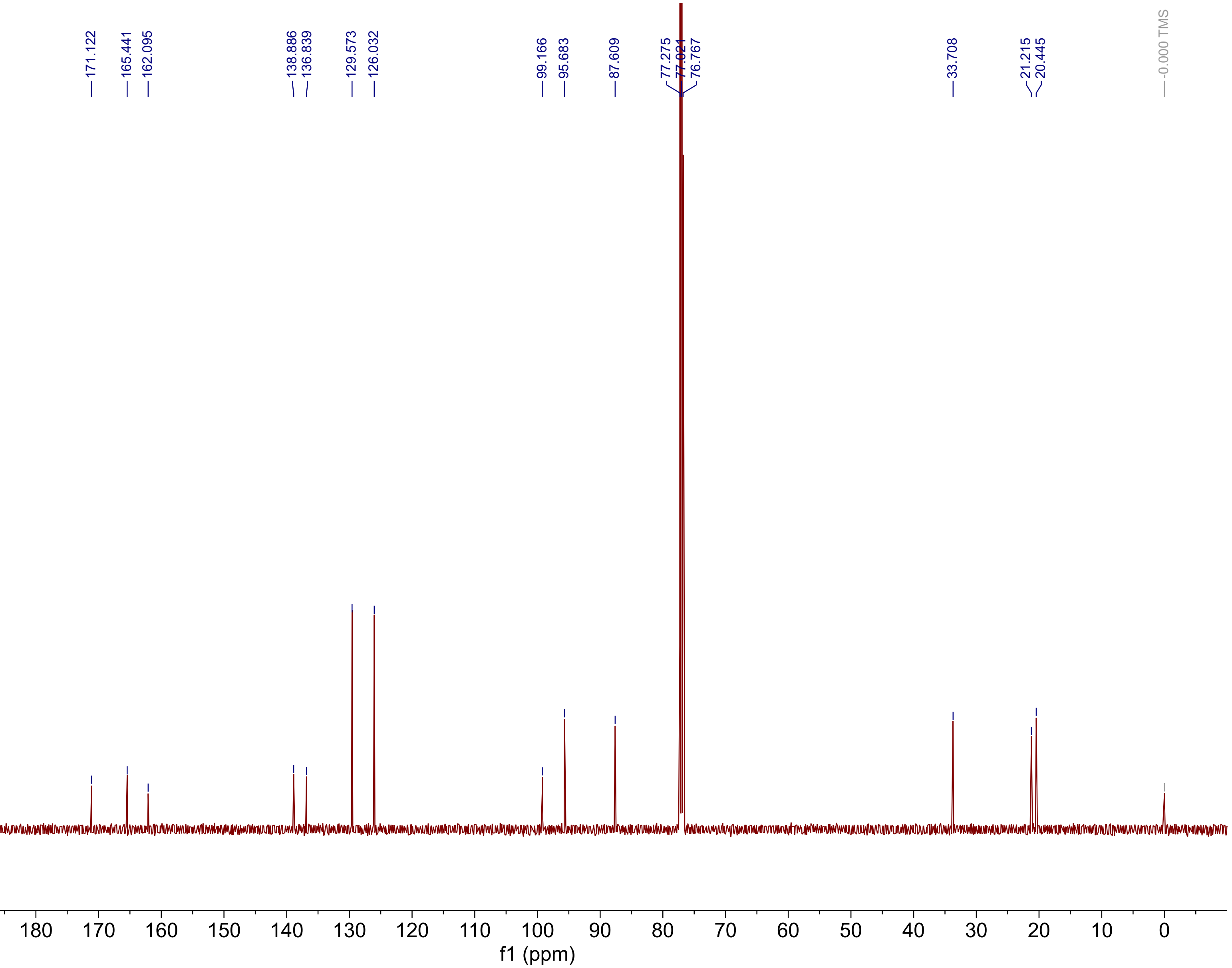

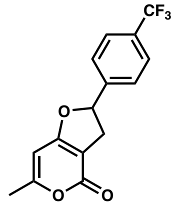

12

^1H NMR
500 MHz, $CDCl_3$

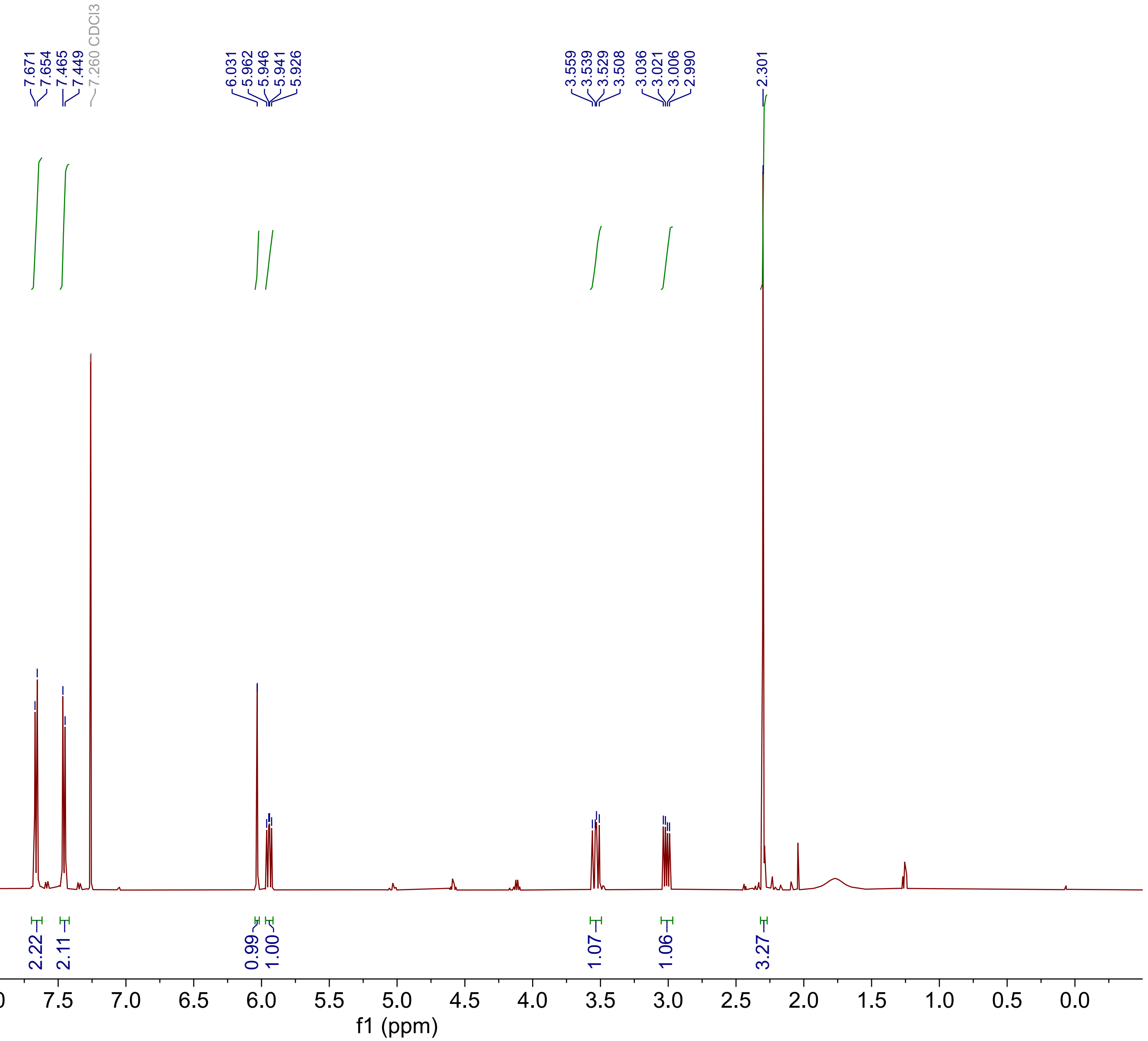

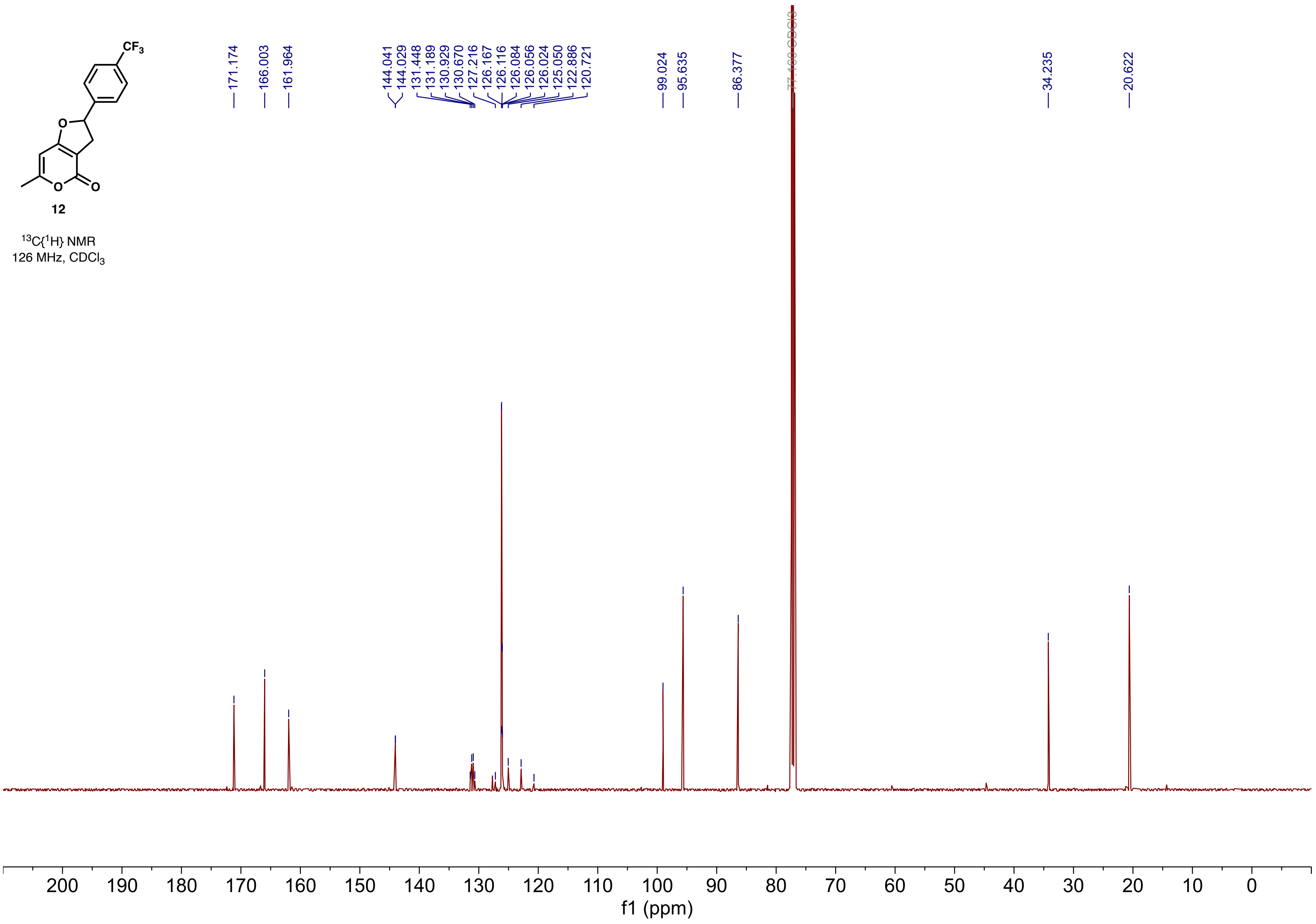
CF3
O
O
O
12
13C{1H} NMR
126 MHz, CDCl3
171.174
166.003
161.964
144.041
144.029
131.448
131.189
130.929
130.670
127.216
126.167
126.116
126.084
126.056
126.024
125.050
122.886
120.721
99.024
95.635
86.377
77.160 CDCl3
34.235
20.622
200
190
180
170
160
150
140
130
120
110
100
90
80
70
60
50
40
30
20
10
0
f1 (ppm)

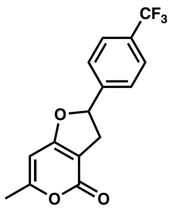

12

^{19}F NMR
471 MHz, $CDCl_3$

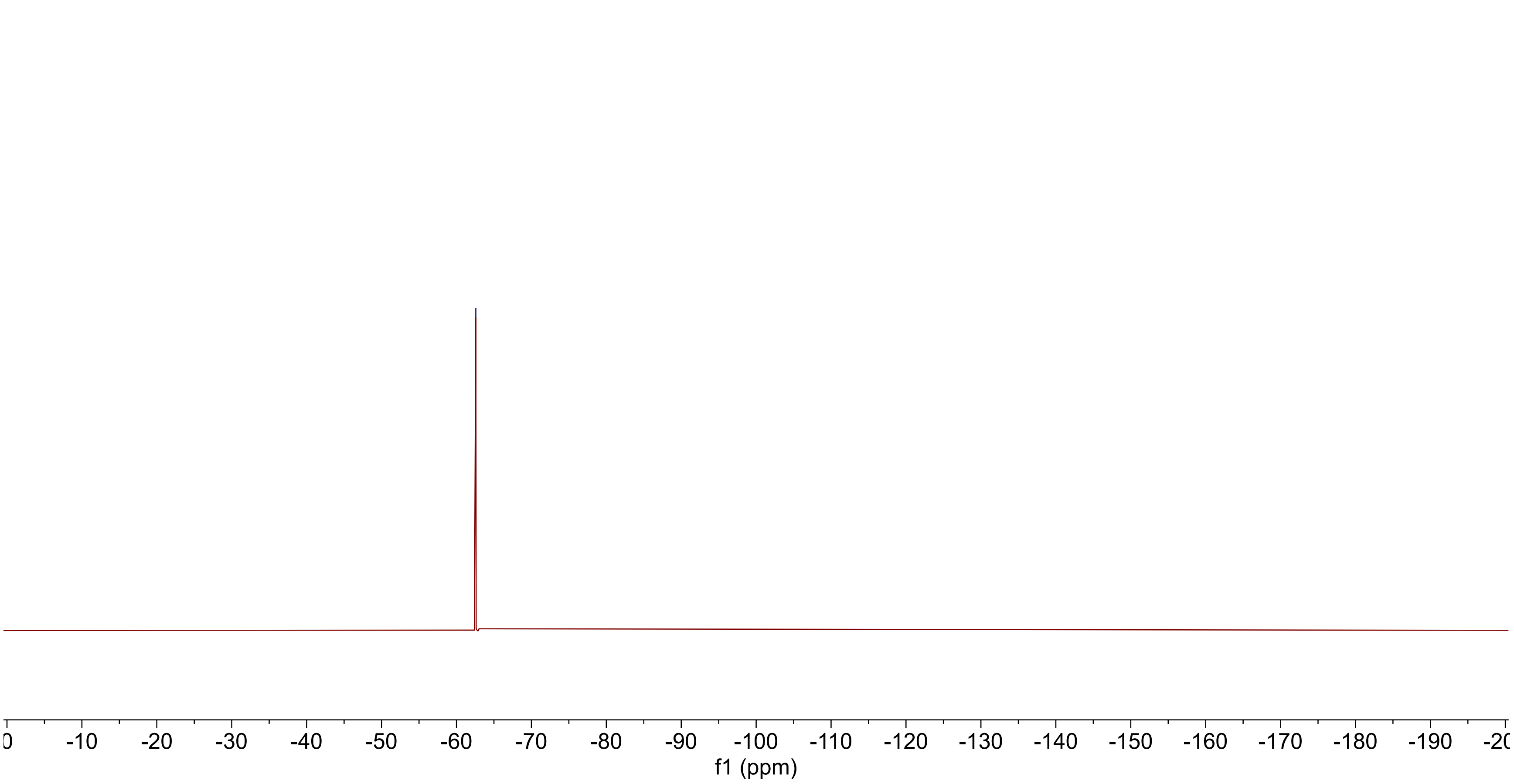

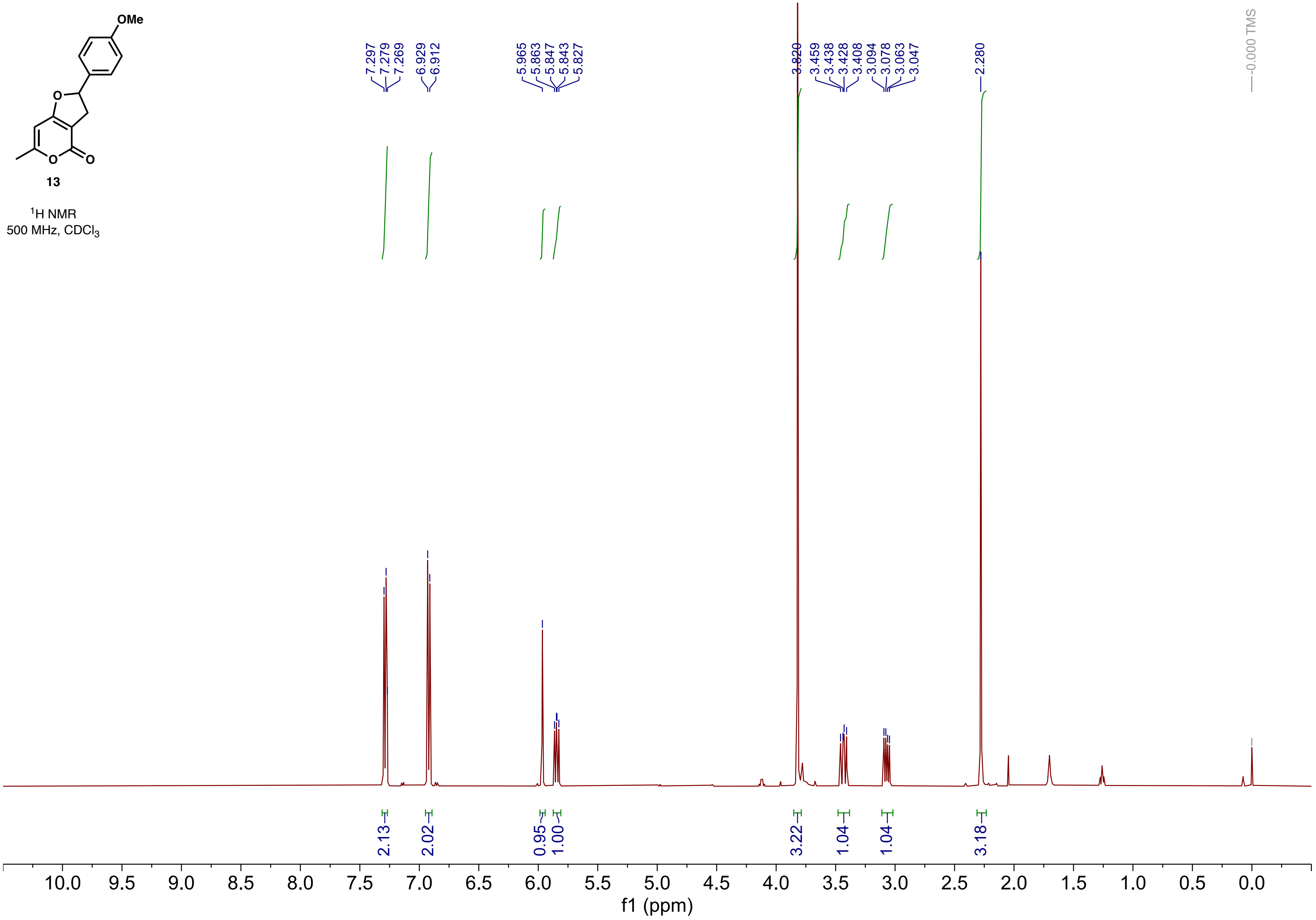
OMe
O
O
O
13
1H NMR
500 MHz, CDCl3
7.297
7.279
7.269
6.929
6.912
5.965
5.863
5.847
5.843
5.827
3.820
3.459
3.438
3.428
3.408
3.094
3.078
3.063
3.047
2.280
0.000 TMS
2.13
2.02
0.95
1.00
3.22
1.04
1.04
3.18
10.0
9.5
9.0
8.5
8.0
7.5
7.0
6.5
6.0
5.5
5.0
4.5
4.0
3.5
3.0
2.5
2.0
1.5
1.0
0.5
0.0
f1 (ppm)

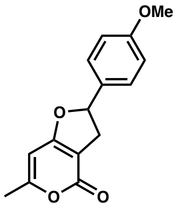

13

$^{13}C\{^1H\}$ NMR
126 MHz, $CDCl_3$

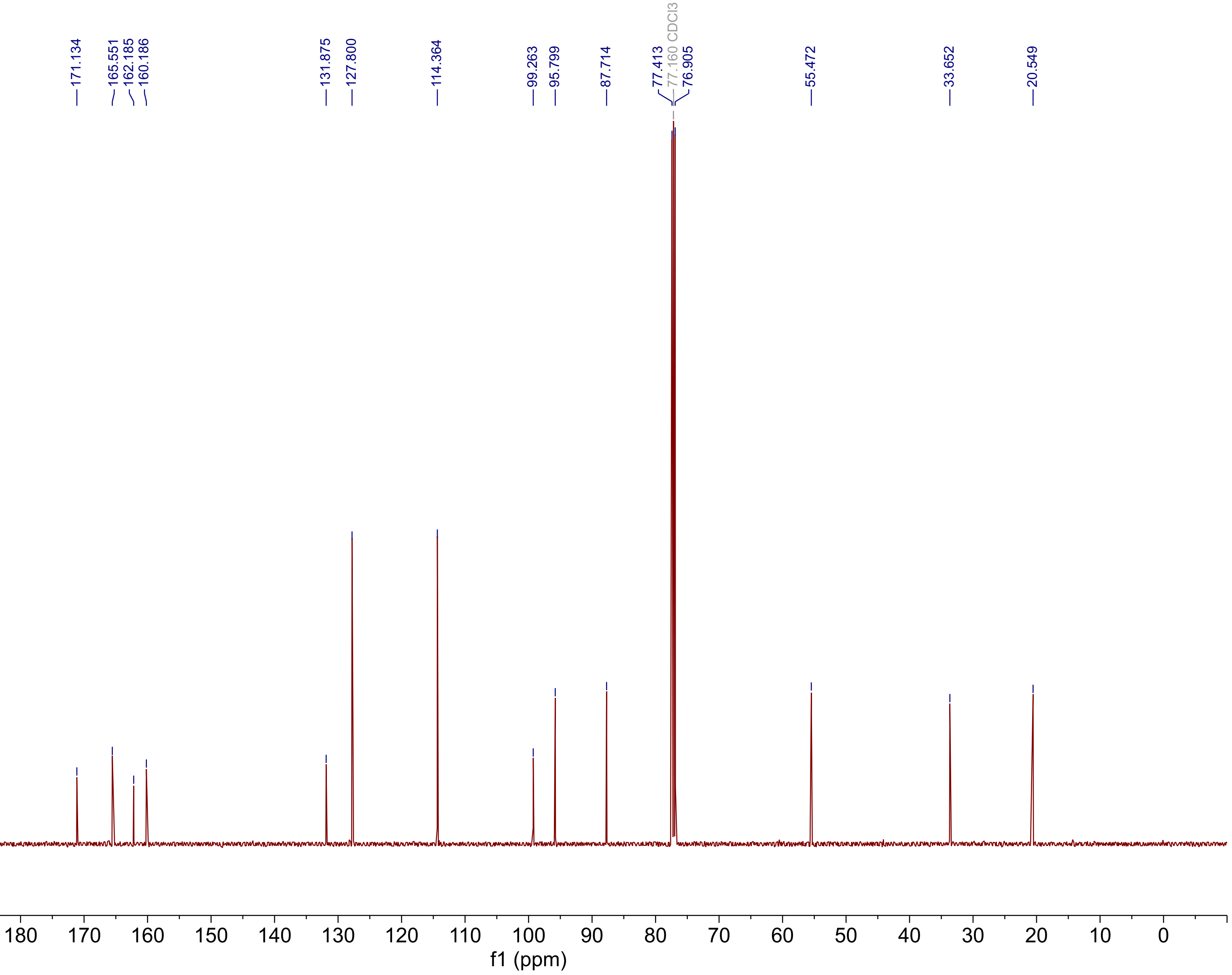

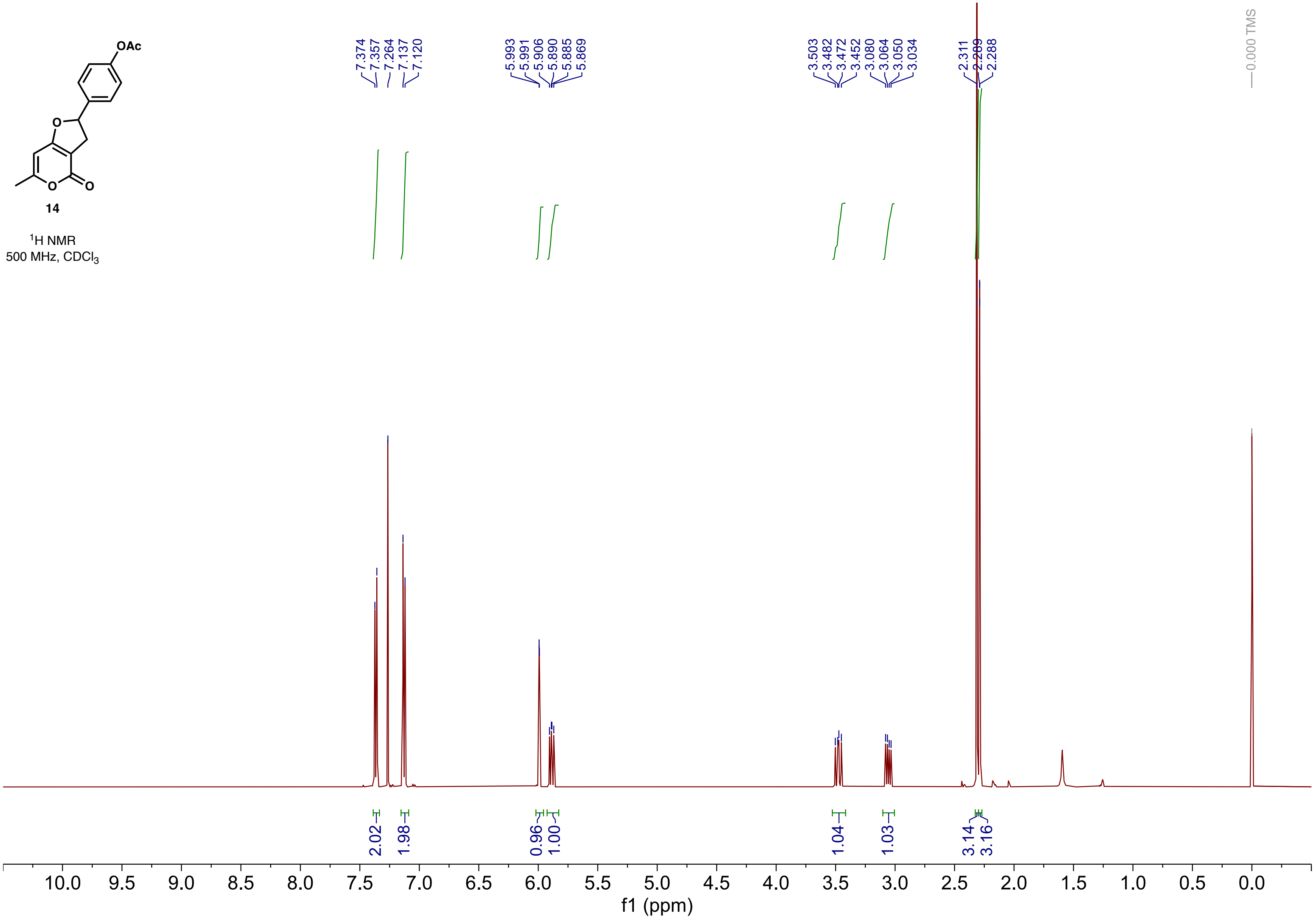
OAc
O
O
O
14
1H NMR
500 MHz, CDCl3
7.374
7.357
7.264
7.137
7.120
5.993
5.991
5.906
5.890
5.885
5.869
3.503
3.482
3.472
3.452
3.080
3.064
3.050
3.034
2.311
2.289
2.288
0.000 TMS
2.02
1.98
0.96
1.00
1.04
1.03
3.14
3.16
10.0 9.5 9.0 8.5 8.0 7.5 7.0 6.5 6.0 5.5 5.0 4.5 4.0 3.5 3.0 2.5 2.0 1.5 1.0 0.5 0.0
f1 (ppm)

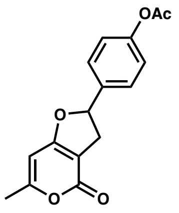

14

$^{13}C\{^{1}H\}$ NMR
126 MHz, $CDCl_3$

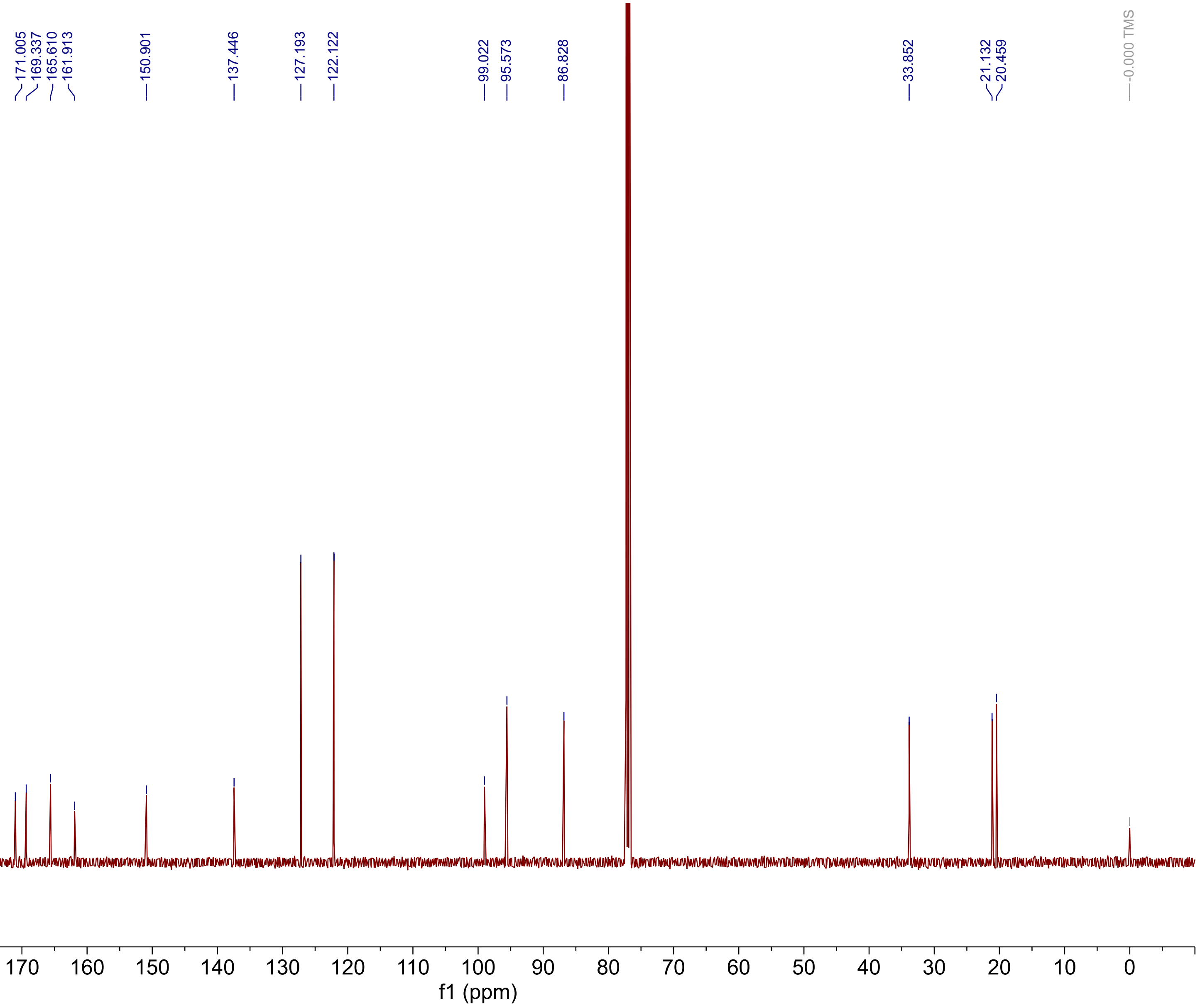

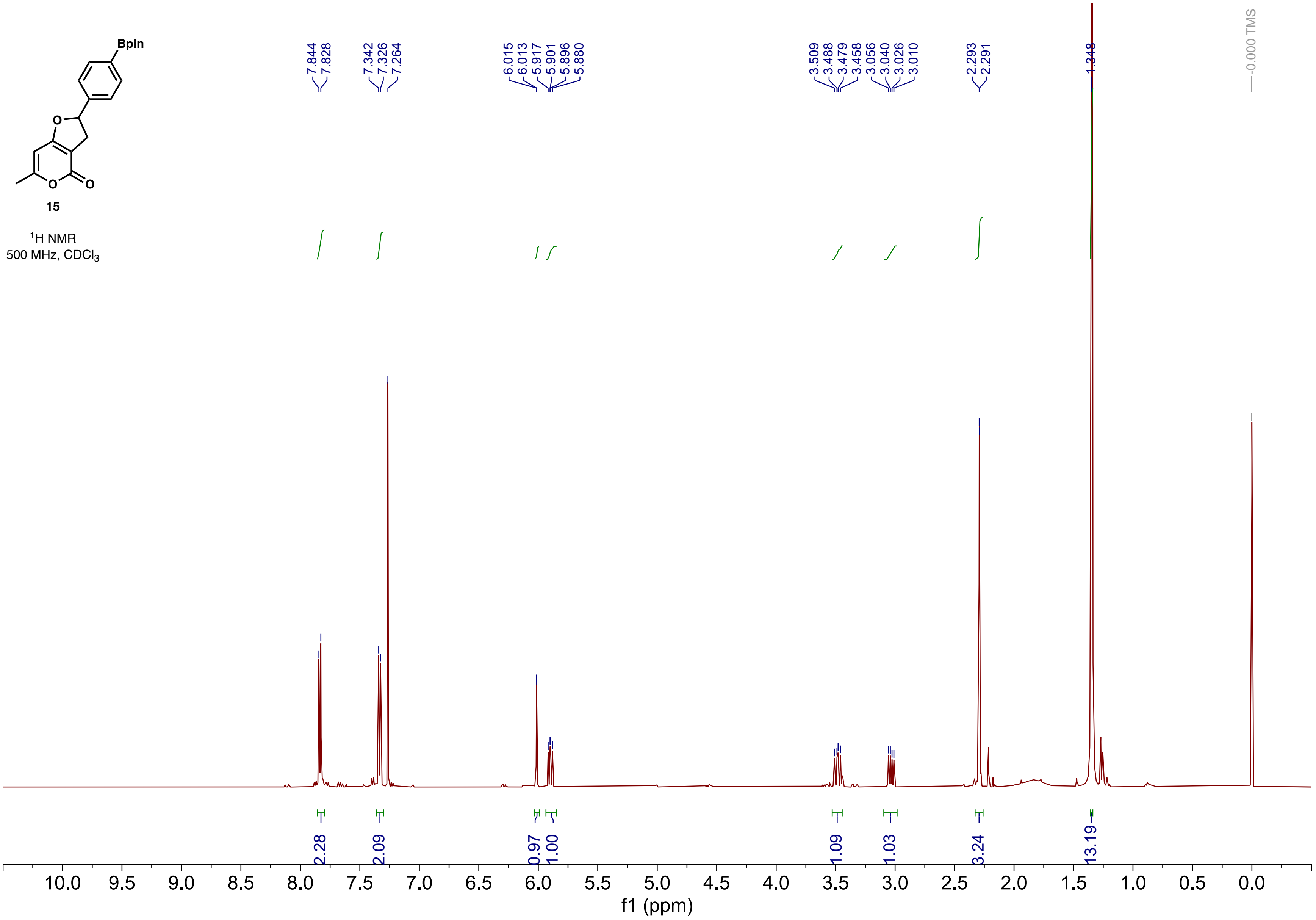
Bpin
O
O
O
15
1H NMR
500 MHz, CDCl3
7.844
7.828
7.342
7.326
7.264
6.015
6.013
5.917
5.901
5.896
5.880
3.509
3.488
3.479
3.458
3.056
3.040
3.026
3.010
2.293
2.291
1.348
0.000 TMS
2.28
2.09
0.97
1.00
1.09
1.03
3.24
13.19
10.0
9.5
9.0
8.5
8.0
7.5
7.0
6.5
6.0
5.5
5.0
4.5
4.0
3.5
3.0
2.5
2.0
1.5
1.0
0.5
0.0
f1 (ppm)

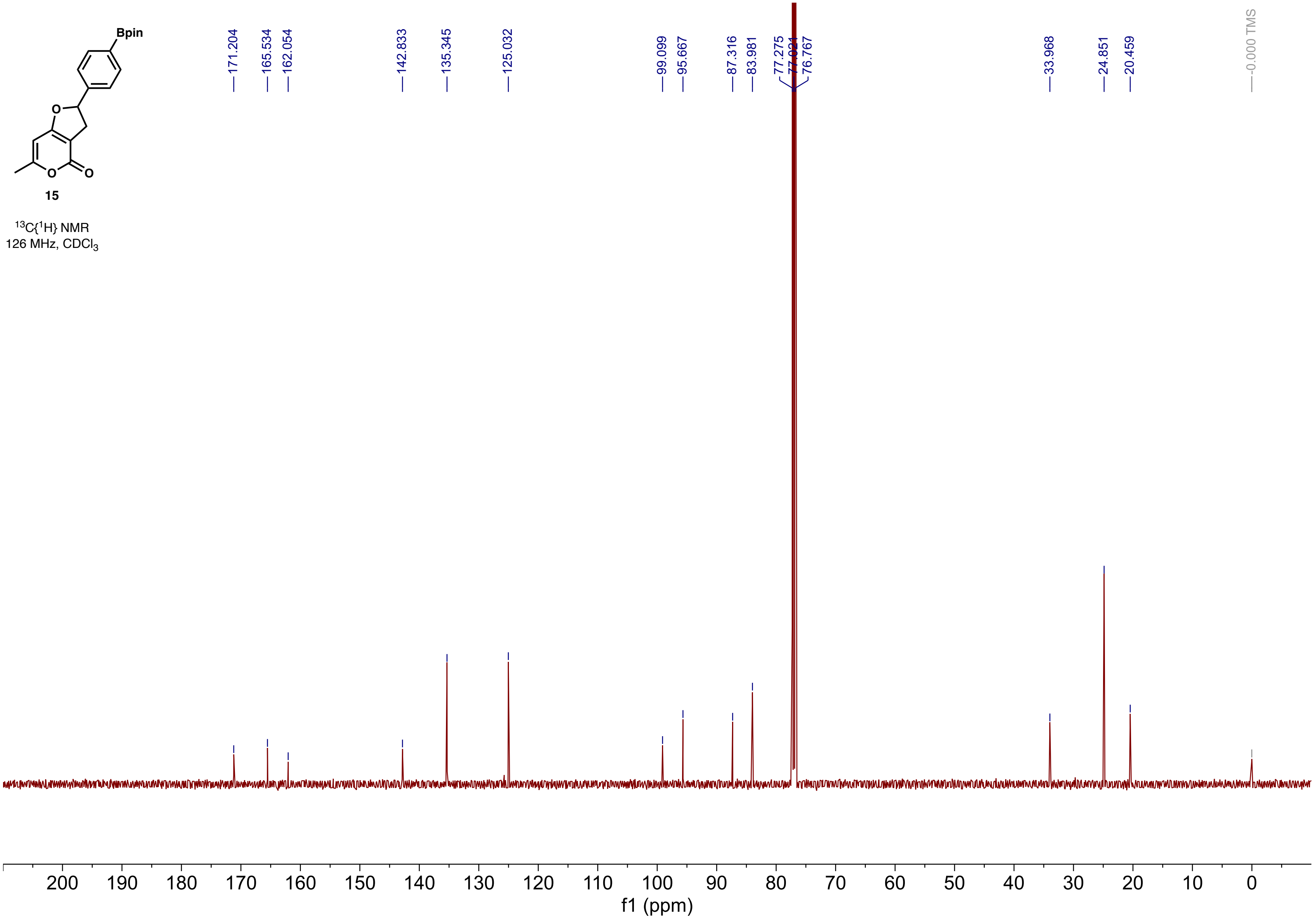
Bpin
15
$^{13}C\{^{1}H\}$ NMR
126 MHz, $CDCl_3$
171.204
165.534
162.054
142.833
135.345
125.032
99.099
95.667
87.316
83.981
77.275
76.767
33.968
24.851
20.459
0.000 TMS
200
190
180
170
160
150
140
130
120
110
100
90
80
70
60
50
40
30
20
10
0
f1 (ppm)

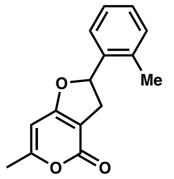

16

^1H NMR
500 MHz, $CDCl_3$

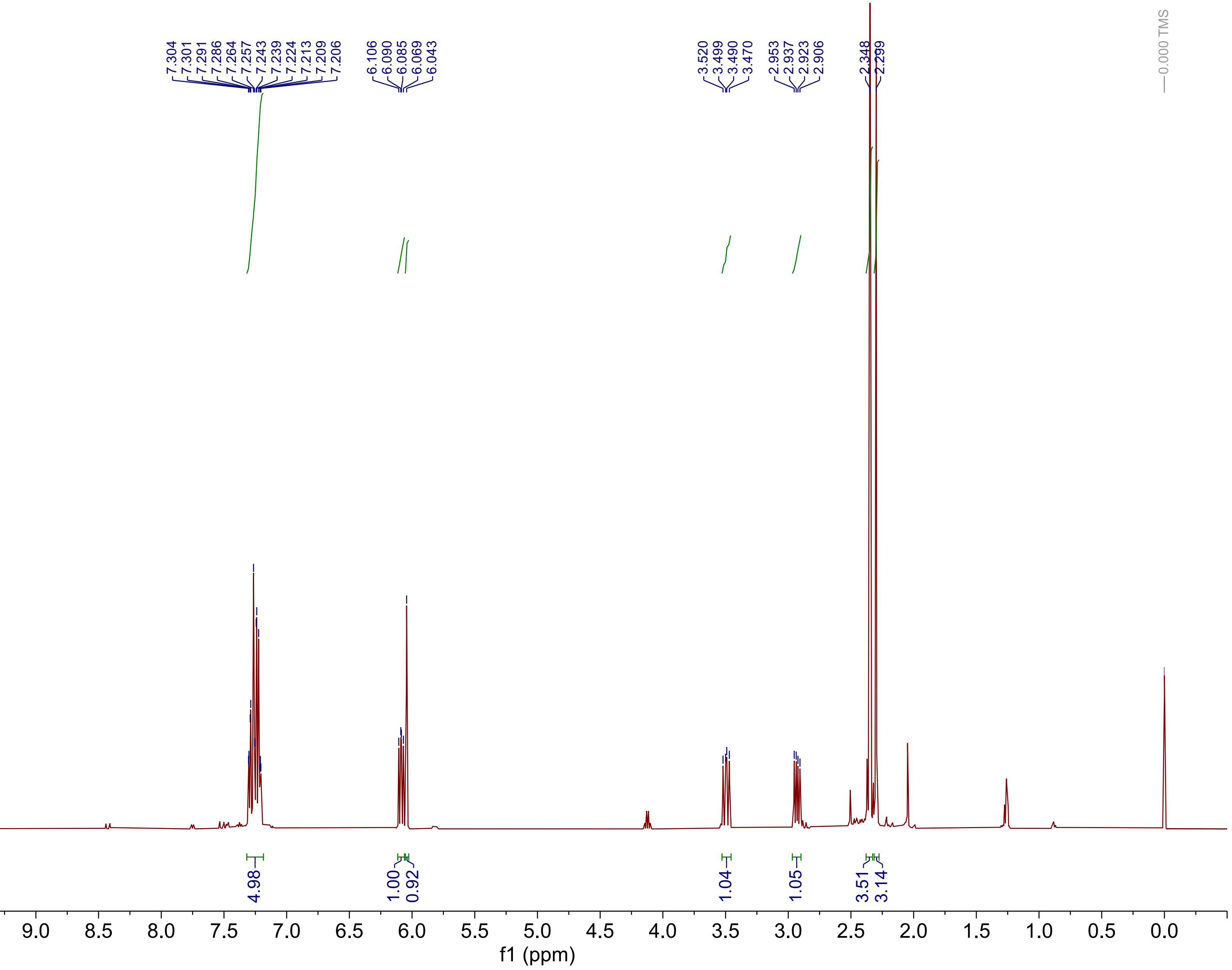

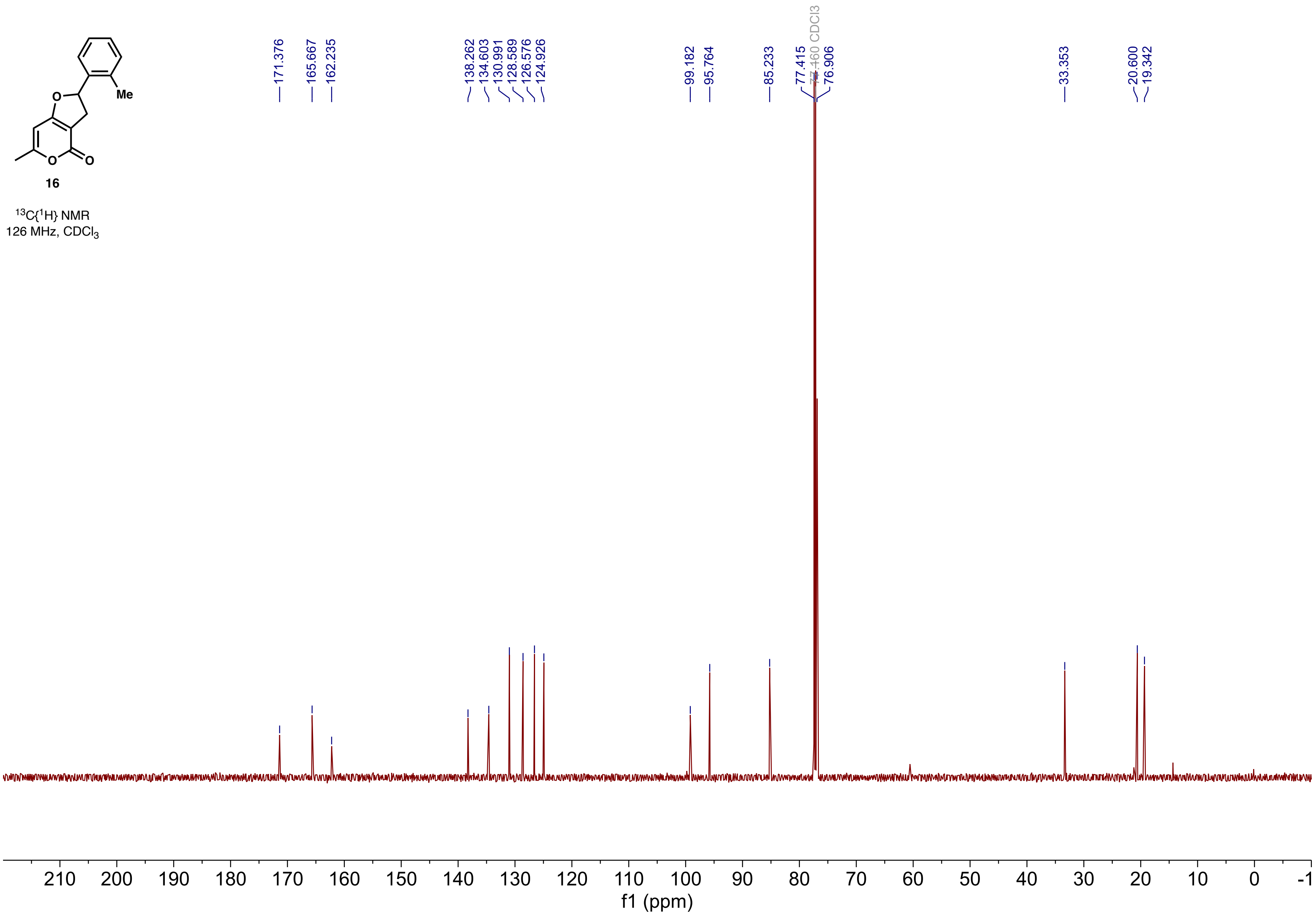
Me
O
O
O
16
^{13}C{^{1}H} NMR
126 MHz, CDCl$_3$
171.376
165.667
162.235
138.262
134.603
130.991
128.589
126.576
124.926
99.182
95.764
85.233
77.415
77.160 CDCl3
76.906
33.353
20.600
19.342
210 200 190 180 170 160 150 140 130 120 110 100 90 80 70 60 50 40 30 20 10 0 -1
f1 (ppm)

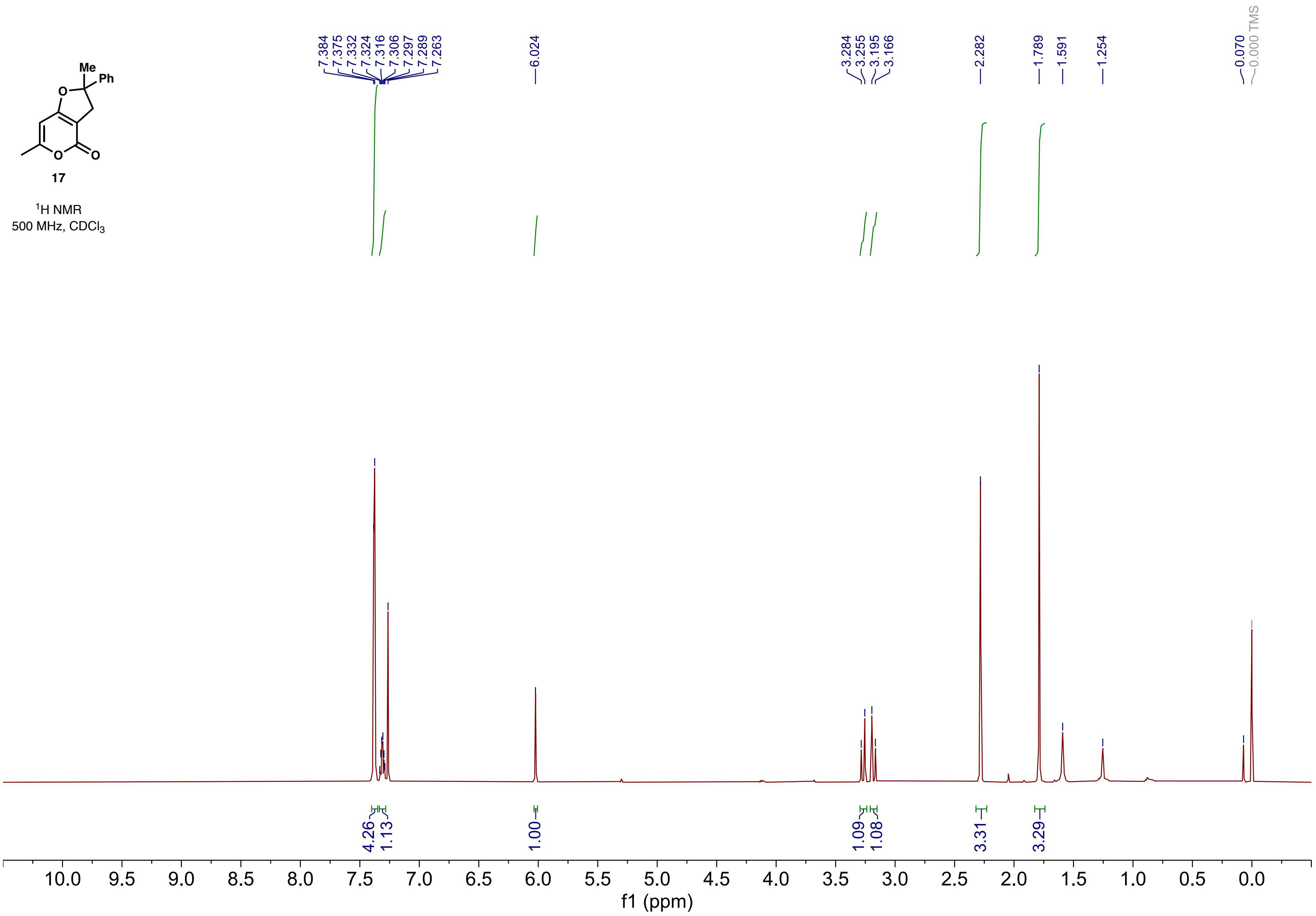

Me
Ph
O
O
O
17
¹H NMR
500 MHz, CDCl3
7.384
7.375
7.332
7.324
7.316
7.306
7.297
7.289
7.263
6.024
3.284
3.255
3.195
3.166
2.282
1.789
1.591
1.254
0.070
0.000 TMS
4.26
1.13
1.00
1.09
1.08
3.31
3.29
10.0 9.5 9.0 8.5 8.0 7.5 7.0 6.5 6.0 5.5 5.0 4.5 4.0 3.5 3.0 2.5 2.0 1.5 1.0 0.5 0.0
f1 (ppm)

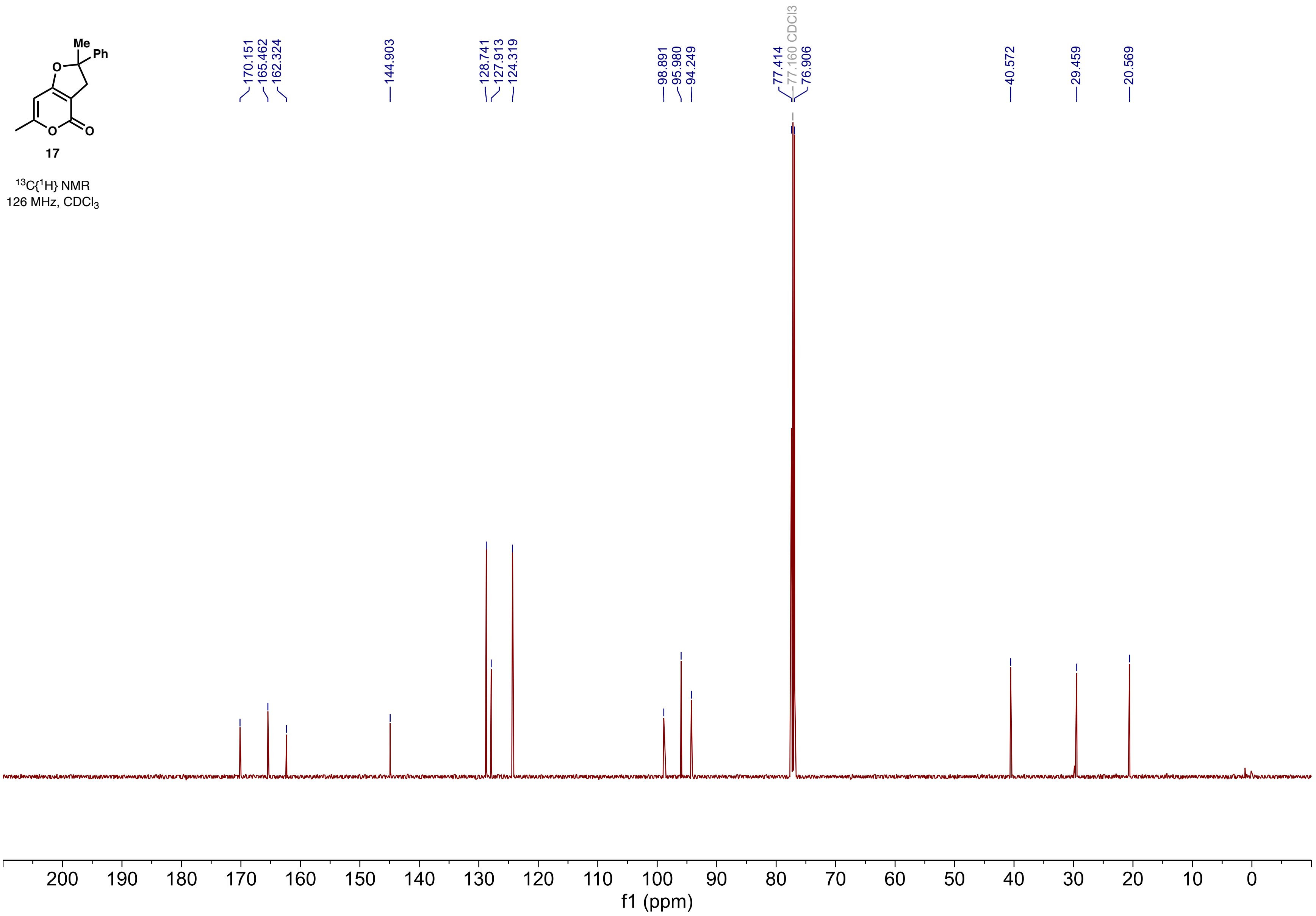
Me
Ph
O
O
O
17
$^{13}C\{^1H\}$ NMR
126 MHz, $CDCl_3$
170.151
165.462
162.324
144.903
128.741
127.913
124.319
98.891
95.980
94.249
77.414
77.160 CDCl3
76.906
40.572
29.459
20.569
200 190 180 170 160 150 140 130 120 110 100 90 80 70 60 50 40 30 20 10 0
f1 (ppm)

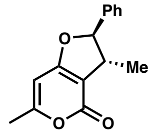

18

^{1}H NMR
500 MHz, $CDCl_3$

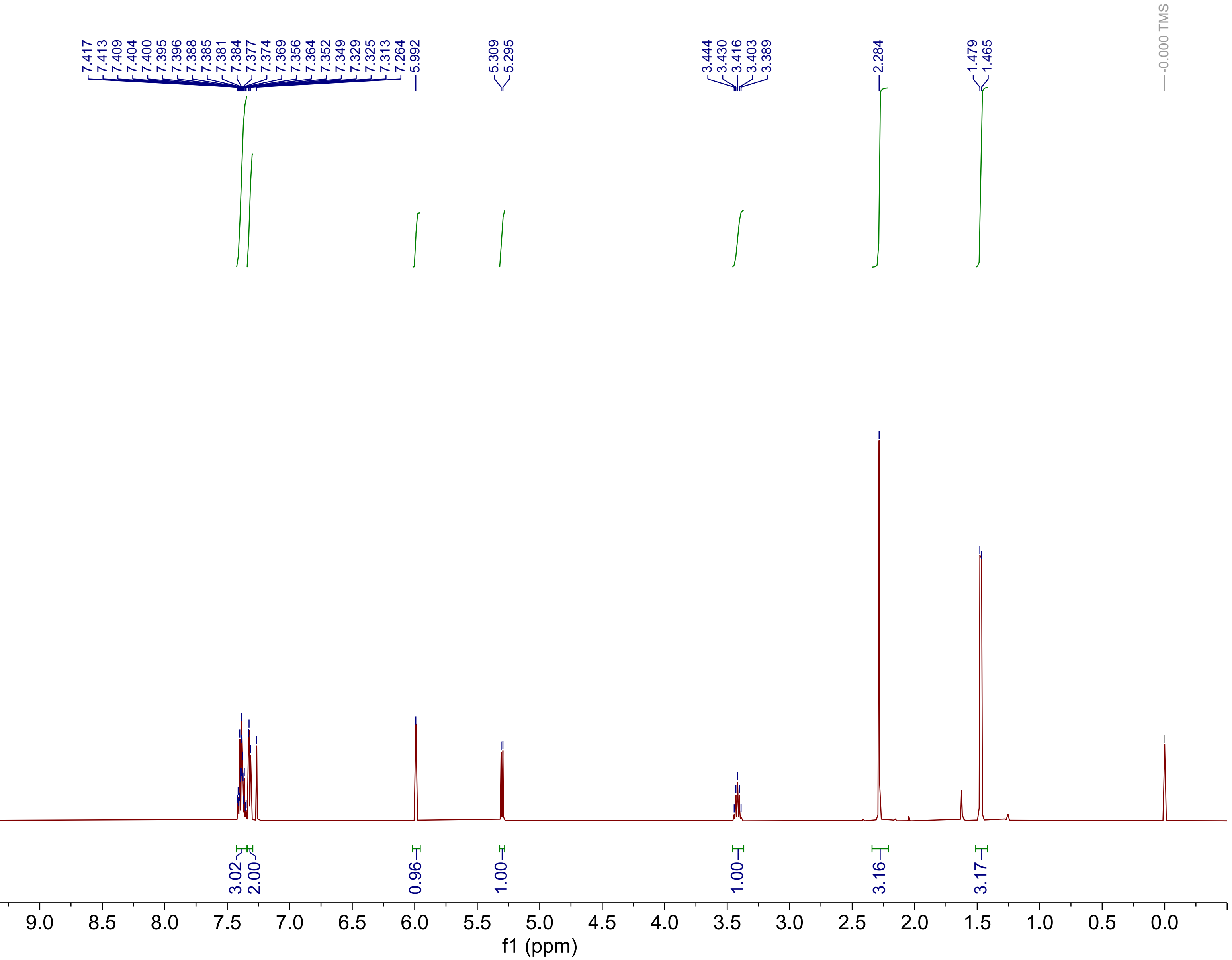

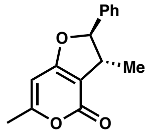

18

$^{13}C\{^1H\}$ NMR
126 MHz, $CDCl_3$

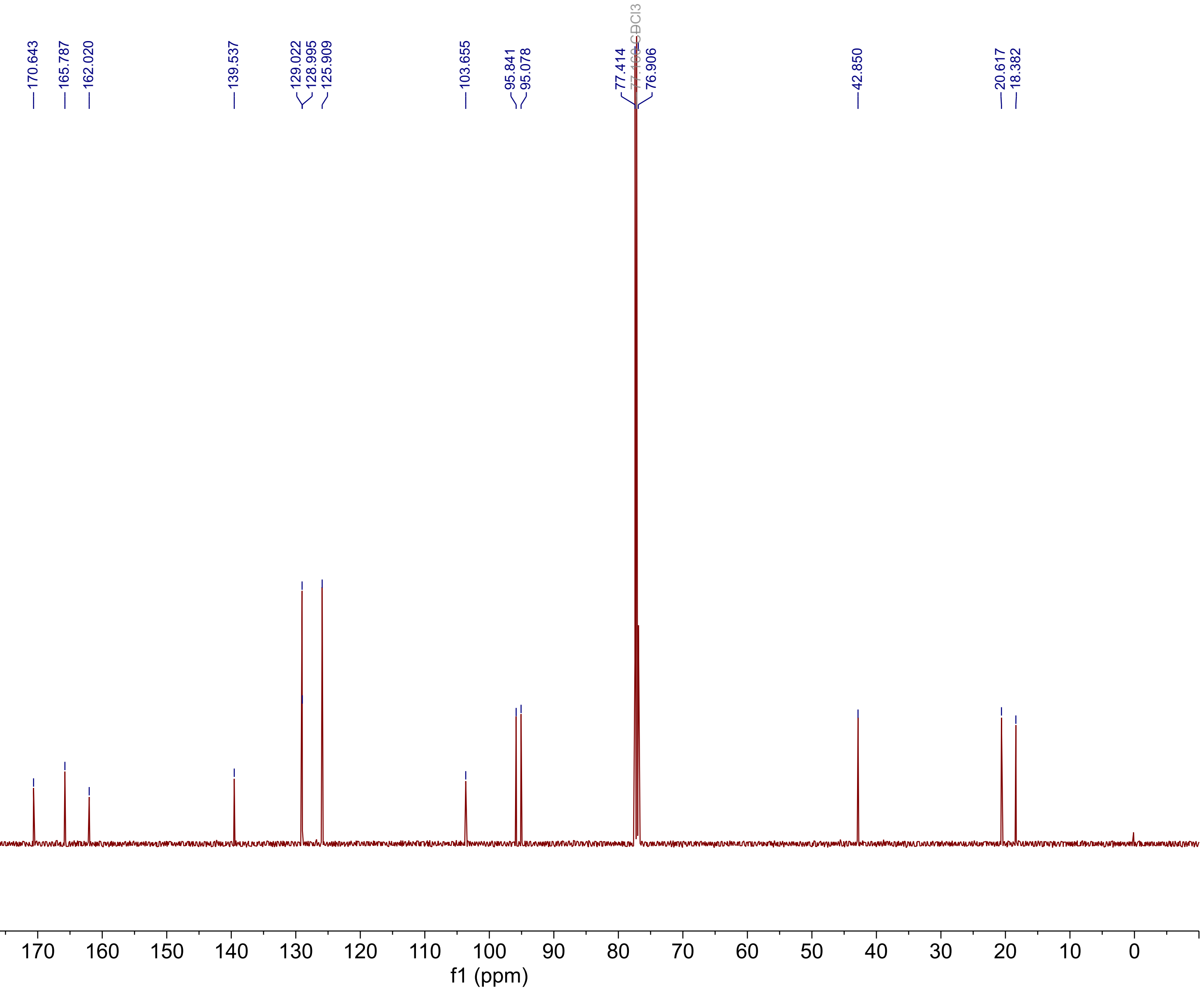

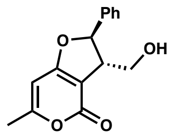

19

^{1}H NMR
500 MHz, $CDCl_3$

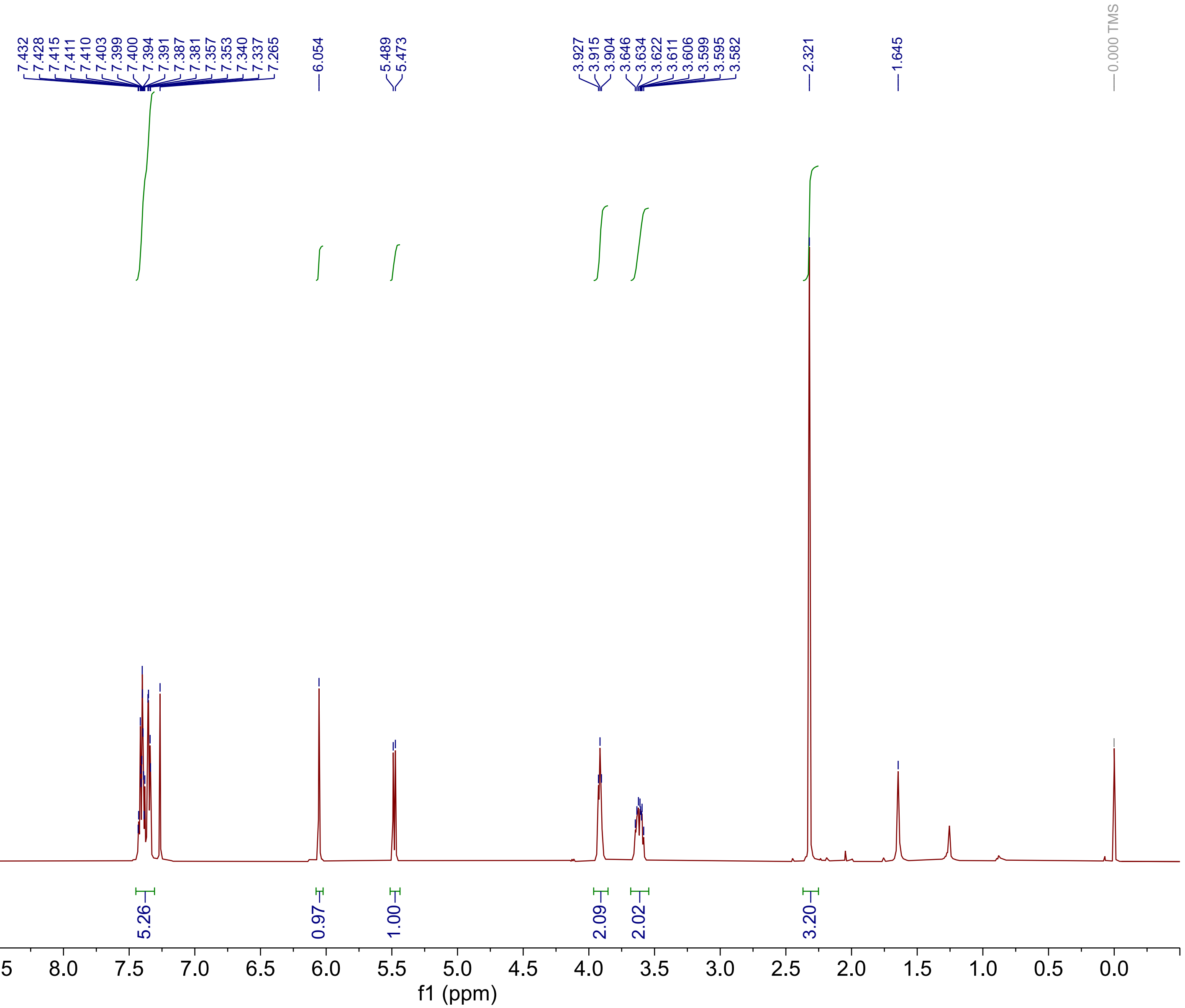

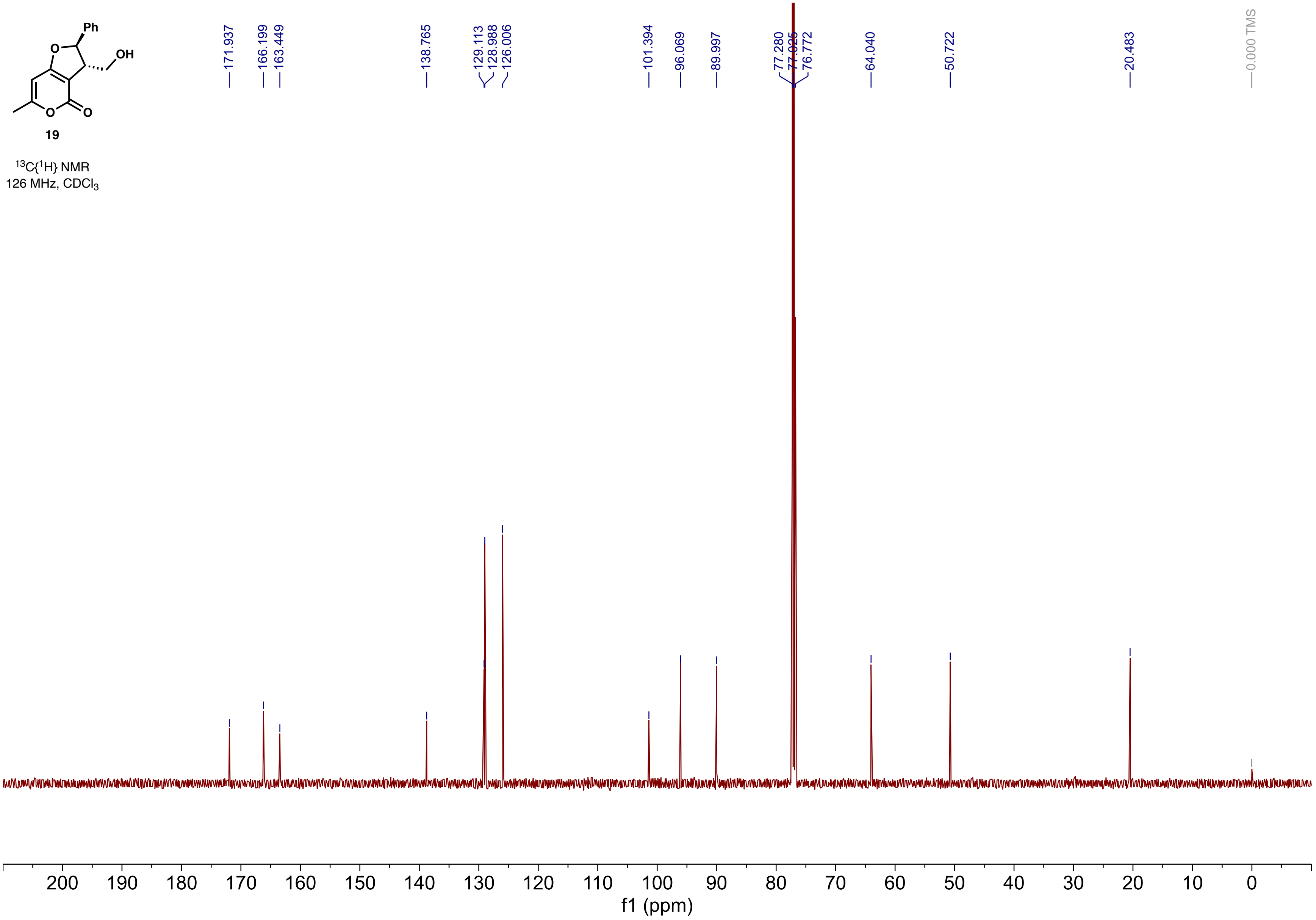
Ph
O
OH
O
O
19
$^{13}C\{^1H\}$ NMR
126 MHz, CDCl3
171.937
166.199
163.449
138.765
129.113
128.988
126.006
101.394
96.069
89.997
77.280
76.772
64.040
50.722
20.483
0.000 TMS
f1 (ppm)

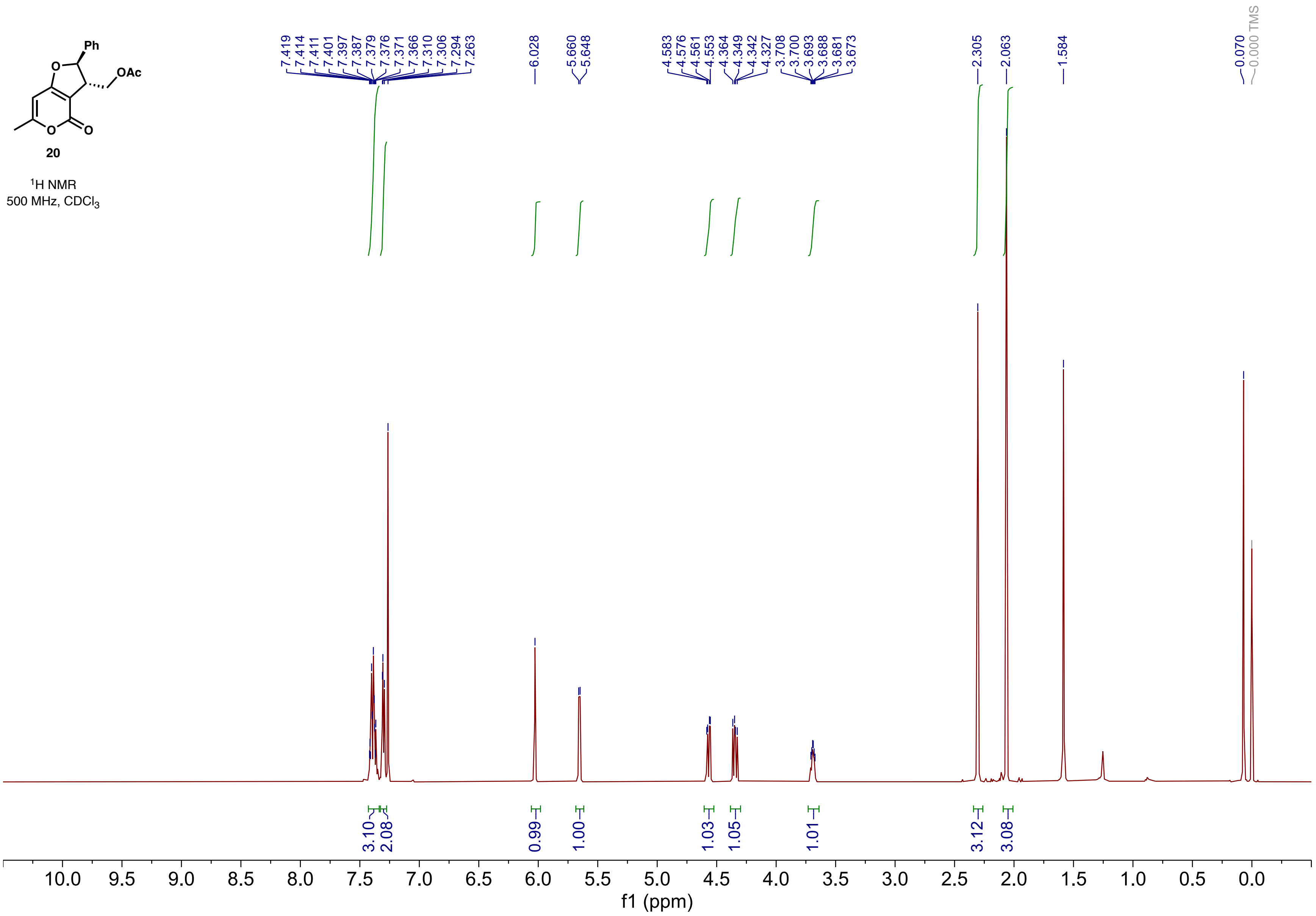
Ph
O
OAc
O
O
20
^{1}H NMR
500 MHz, CDCl$_3$
7.419 7.414 7.411 7.401 7.397 7.387 7.379 7.376 7.371 7.366 7.310 7.306 7.294 7.263
6.028
5.660 5.648
4.583 4.576 4.561 4.553 4.364 4.349 4.342 4.327 3.708 3.700 3.693 3.688 3.681 3.673
2.305
2.063
1.584
0.070
0.000 TMS
3.10 2.08 0.99 1.00 1.03 1.05 1.01 3.12 3.08
10.0 9.5 9.0 8.5 8.0 7.5 7.0 6.5 6.0 5.5 5.0 4.5 4.0 3.5 3.0 2.5 2.0 1.5 1.0 0.5 0.0
f1 (ppm)

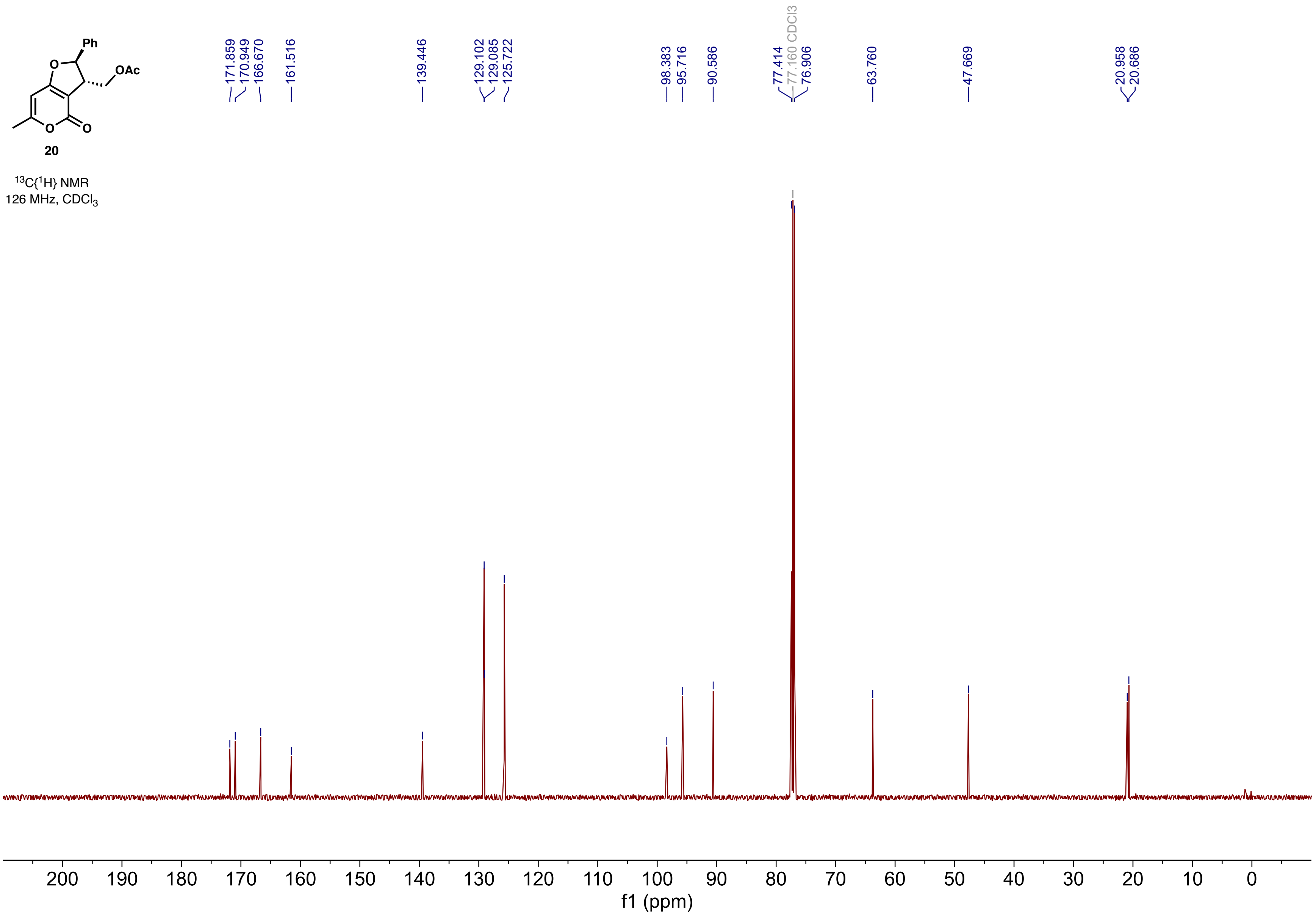
Ph
O
OAc
O
O
20
$^{13}C\{^1H\}$ NMR
126 MHz, $CDCl_3$
171.859
170.949
166.670
161.516
139.446
129.102
129.085
125.722
98.383
95.716
90.586
77.414
77.160 CDCl3
76.906
63.760
47.669
20.958
20.686
200
190
180
170
160
150
140
130
120
110
100
90
80
70
60
50
40
30
20
10
0
f1 (ppm)

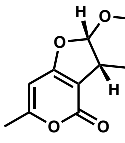

22

^{1}H NMR
500 MHz, $CDCl_3$

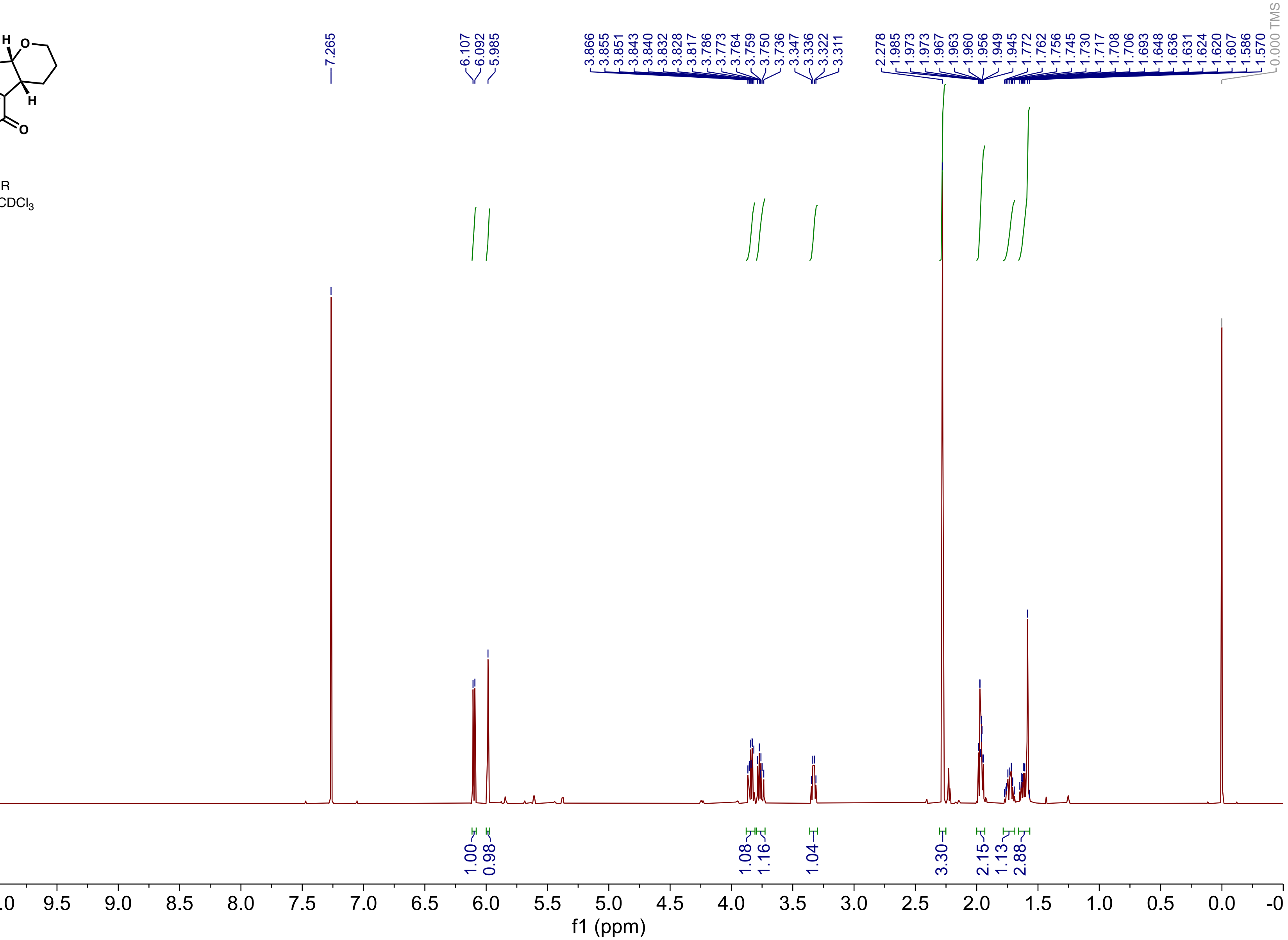

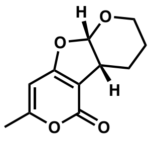

22

$^{13}C\{^1H\}$ NMR
126 MHz, $CDCl_3$

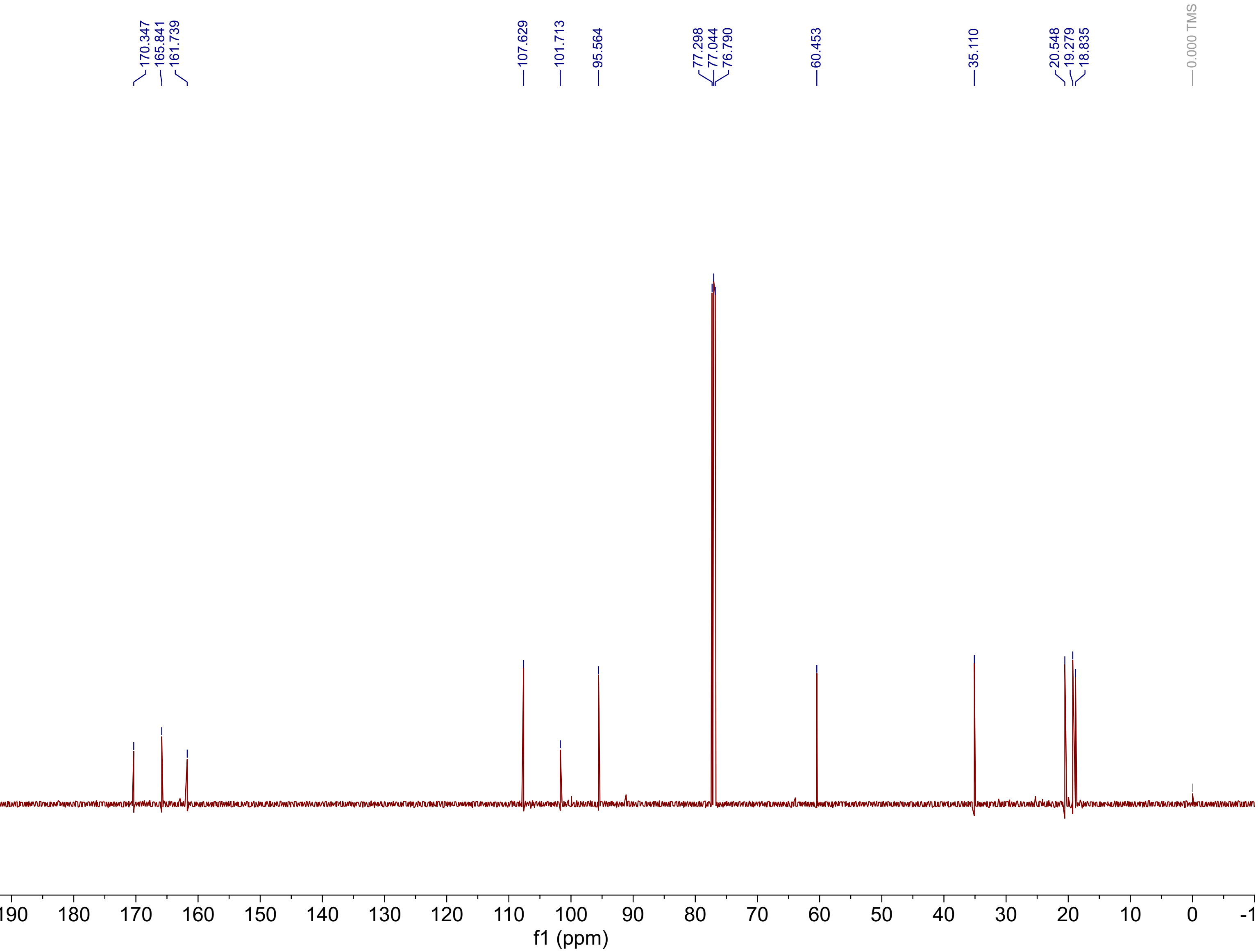

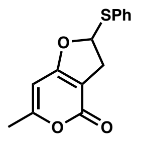

23

^{1}H NMR
500 MHz, $CDCl_3$

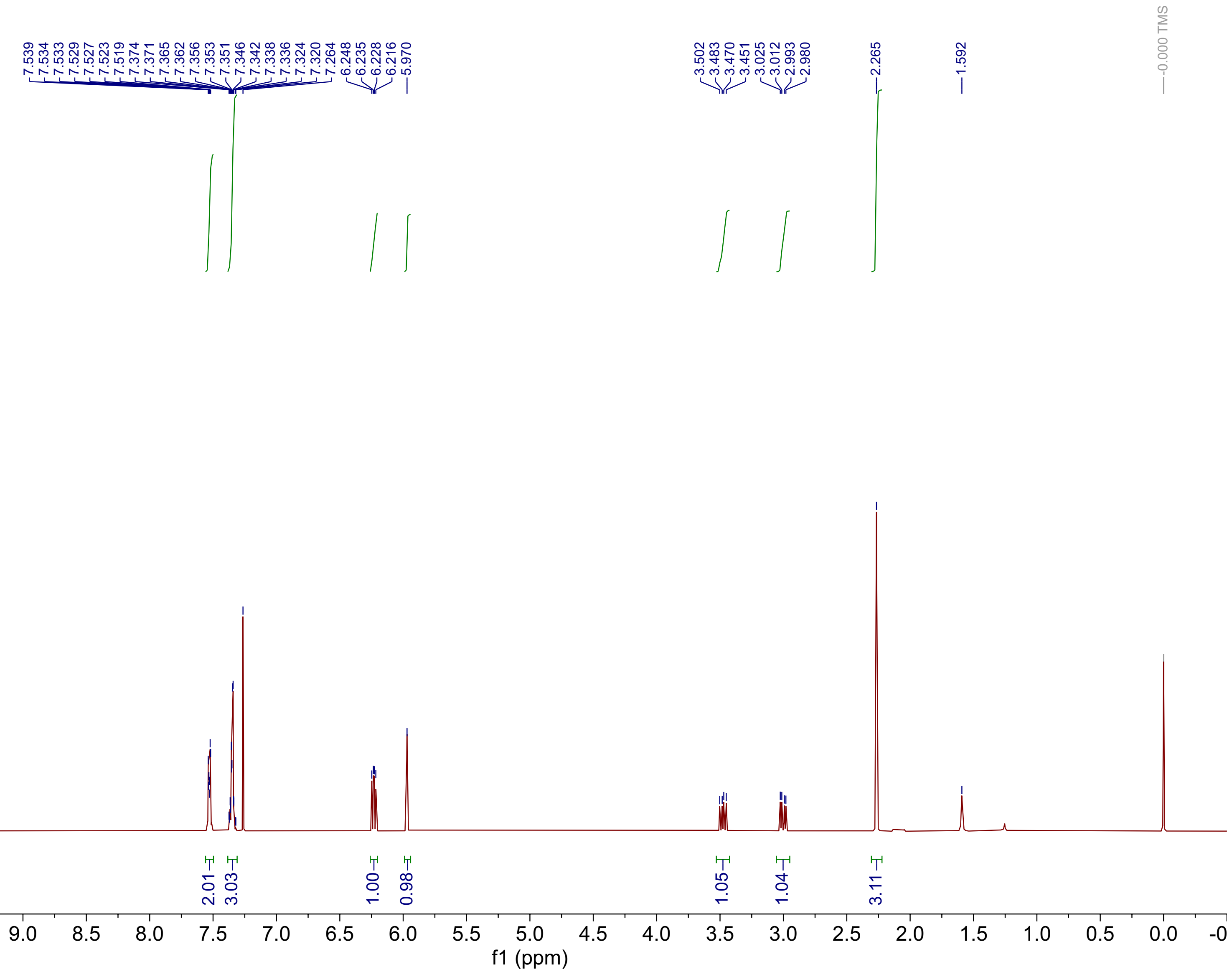

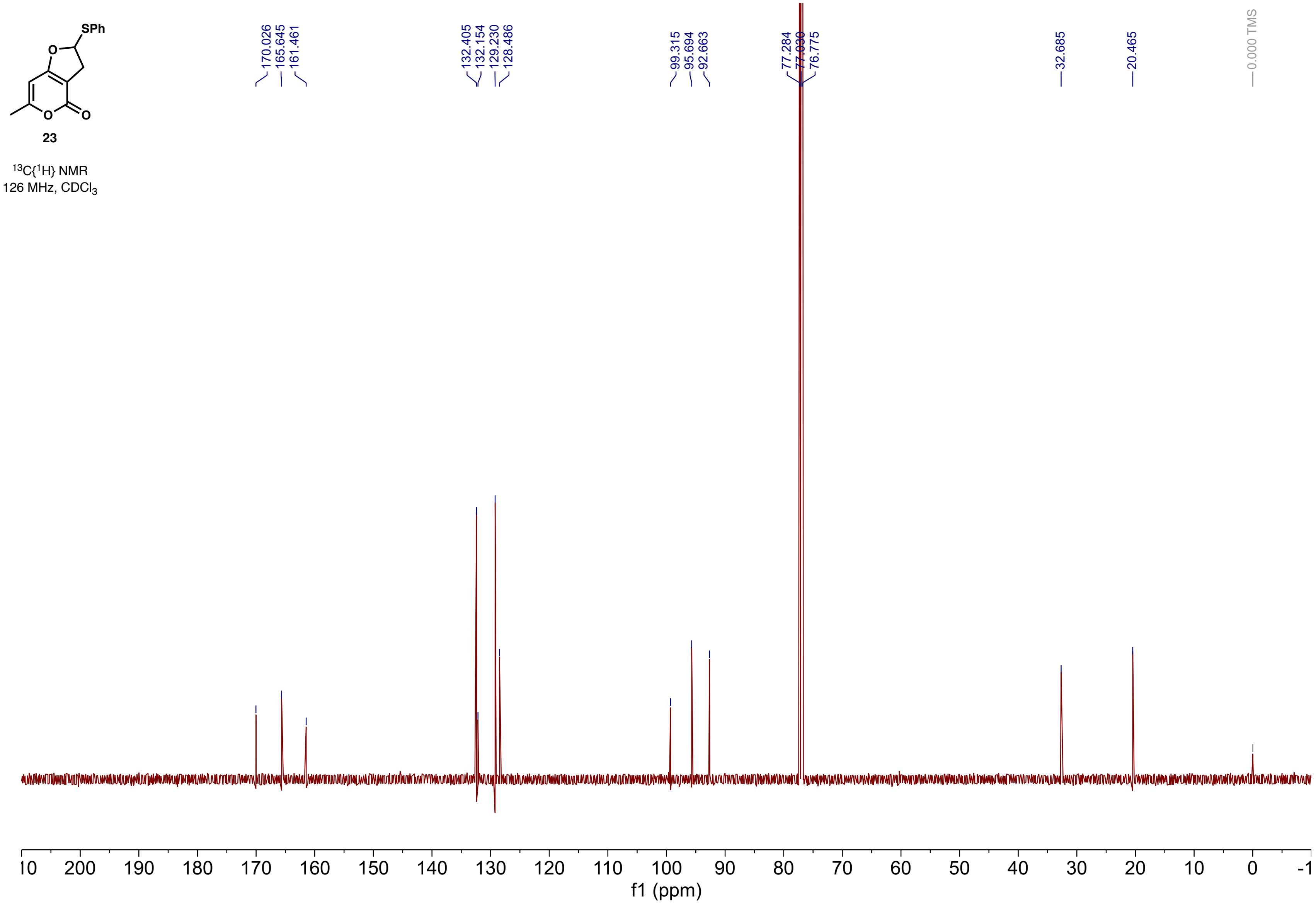

23

$^{13}C\{^{1}H\}$ NMR
126 MHz, $CDCl_3$

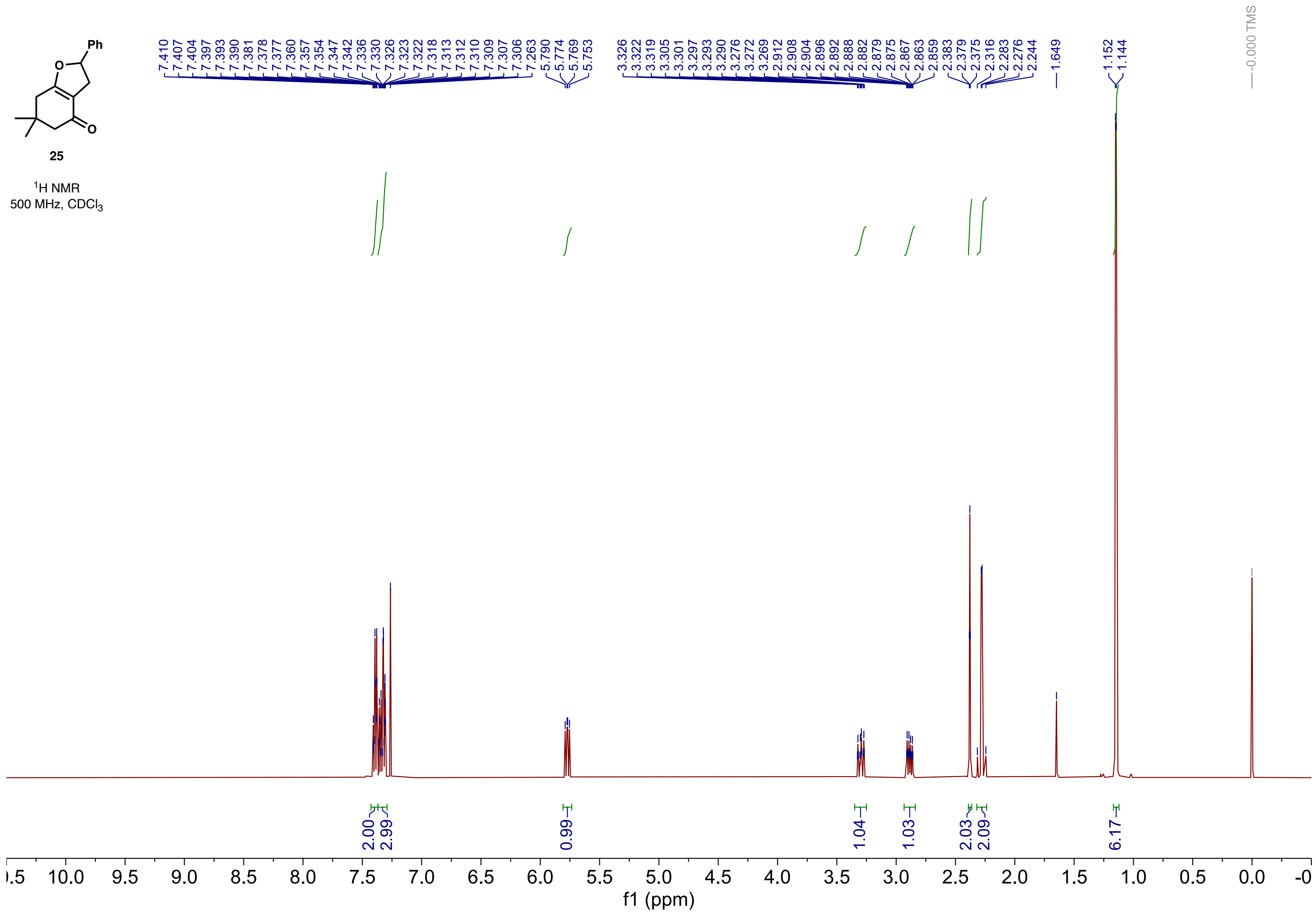
Ph
O
O
25
1H NMR
500 MHz, CDCl3
f1 (ppm)
0.000 TMS

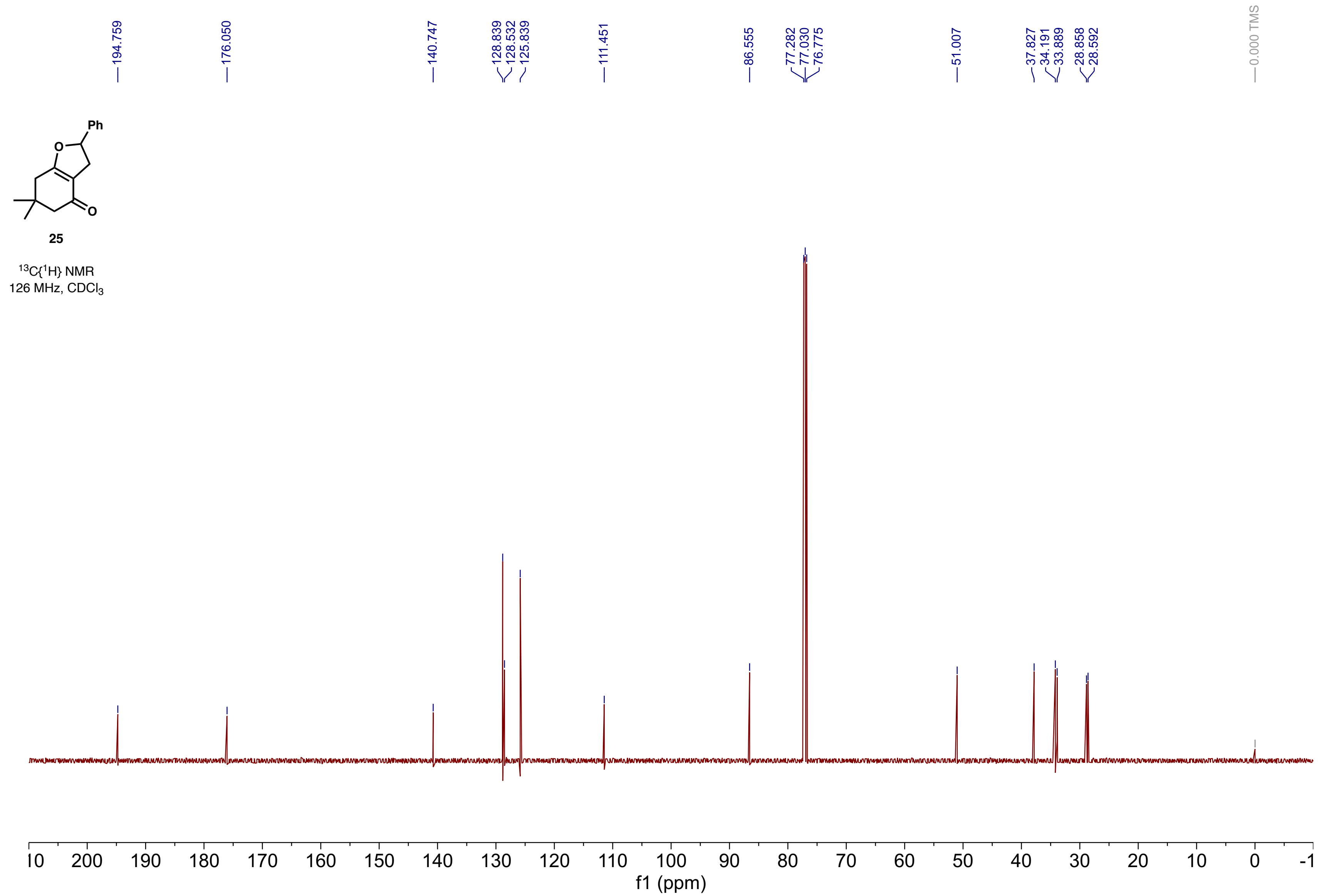

Ph
O
25
$^{13}C\{^1H\}$ NMR
126 MHz, CDCl$_3$
194.759
176.050
140.747
128.839
128.532
125.839
111.451
86.555
77.282
77.030
76.775
51.007
37.827
34.191
33.889
28.858
28.592
0.000 TMS
f1 (ppm)

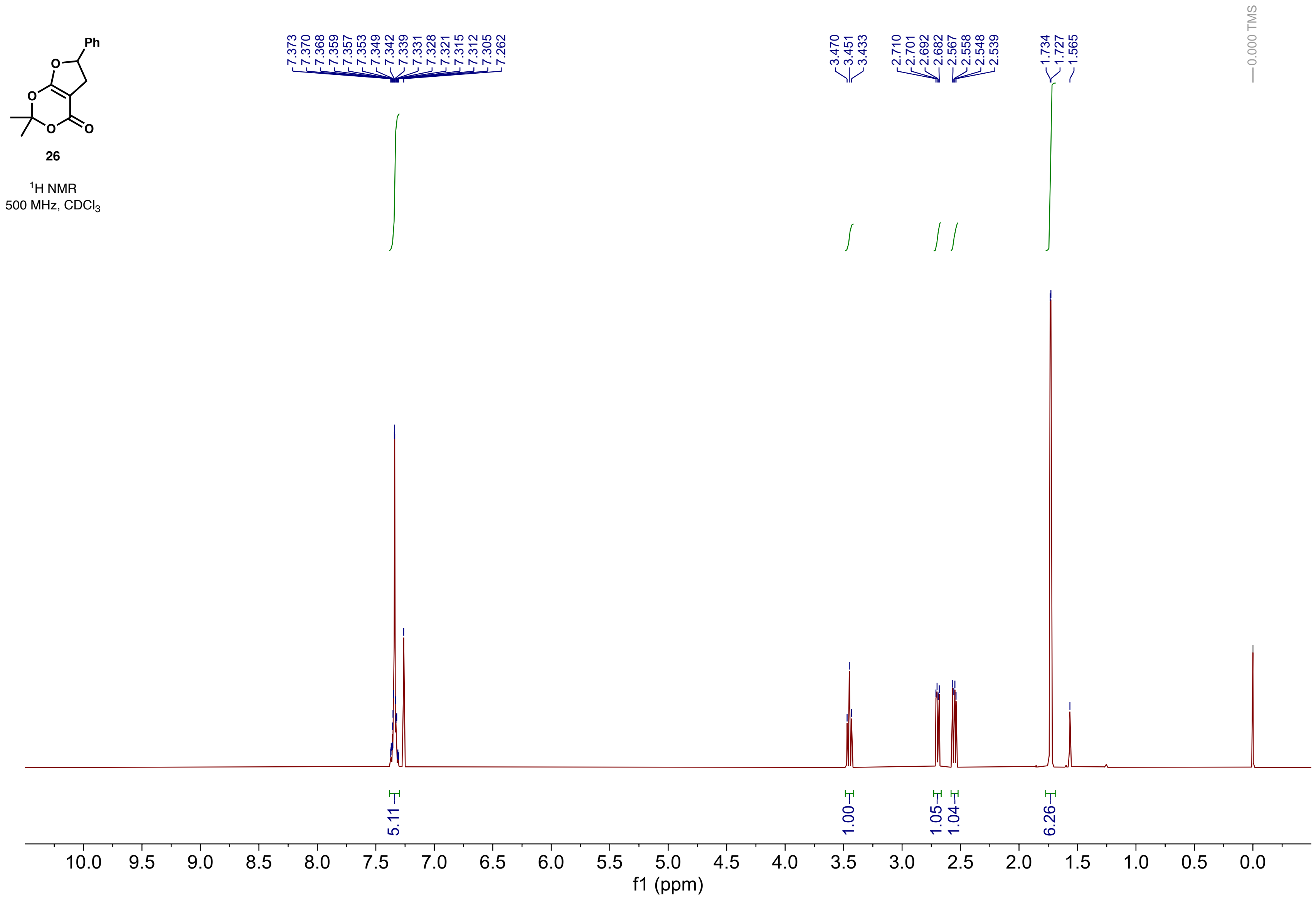
Ph
O
O
O
O
26
1H NMR
500 MHz, CDCl3
7.373
7.370
7.368
7.359
7.357
7.353
7.349
7.342
7.339
7.331
7.328
7.321
7.315
7.312
7.305
7.262
3.470
3.451
3.433
2.710
2.701
2.692
2.682
2.567
2.558
2.548
2.539
1.734
1.727
1.565
0.000 TMS
5.11
1.00
1.05
1.04
6.26
10.0 9.5 9.0 8.5 8.0 7.5 7.0 6.5 6.0 5.5 5.0 4.5 4.0 3.5 3.0 2.5 2.0 1.5 1.0 0.5 0.0
f1 (ppm)

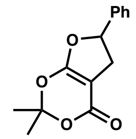

26

$^{13}C\{^{1}H\}$ NMR
126 MHz, $CDCl_3$

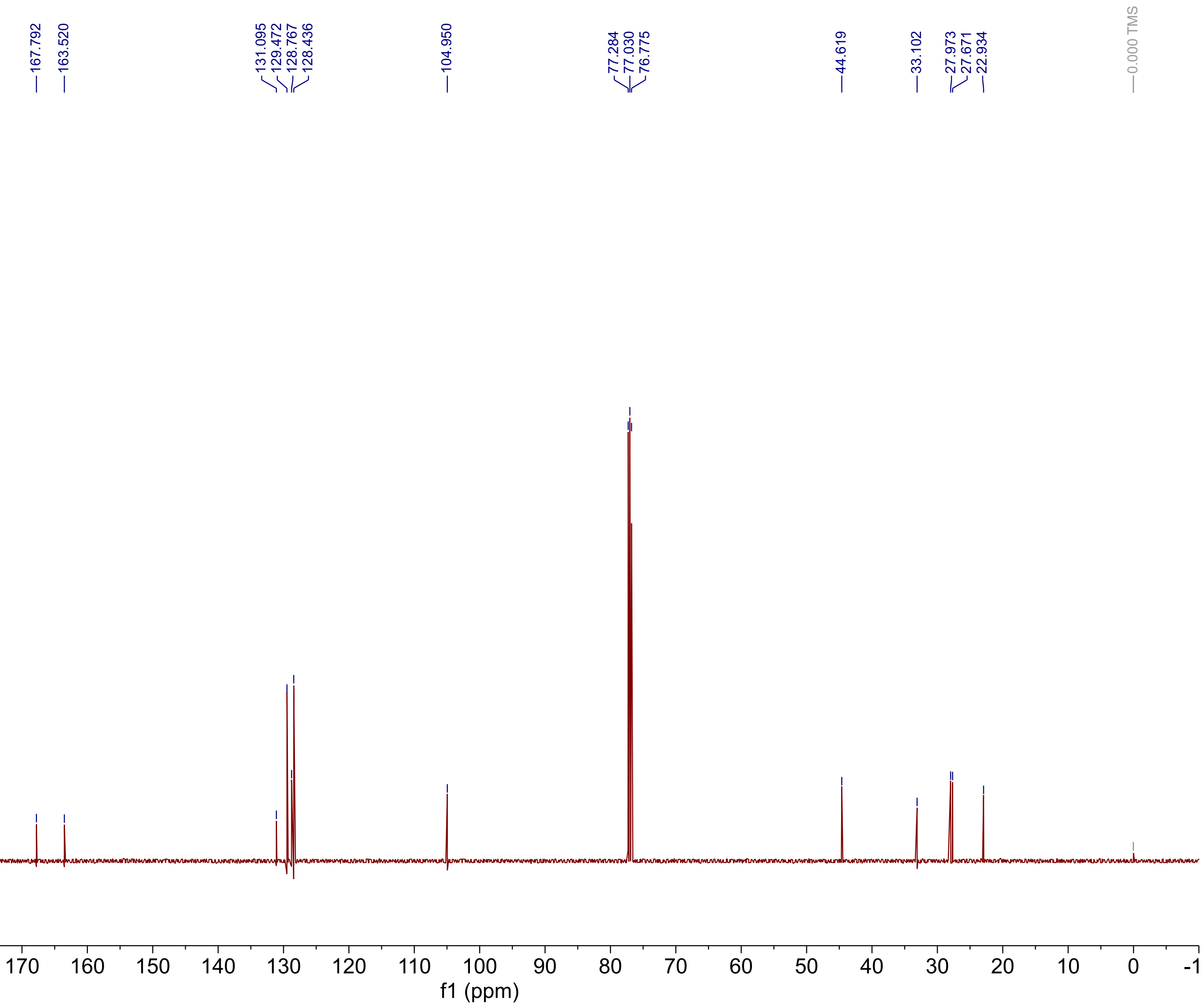

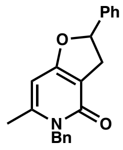

7

^{1}H NMR
500 MHz, $CDCl_3$

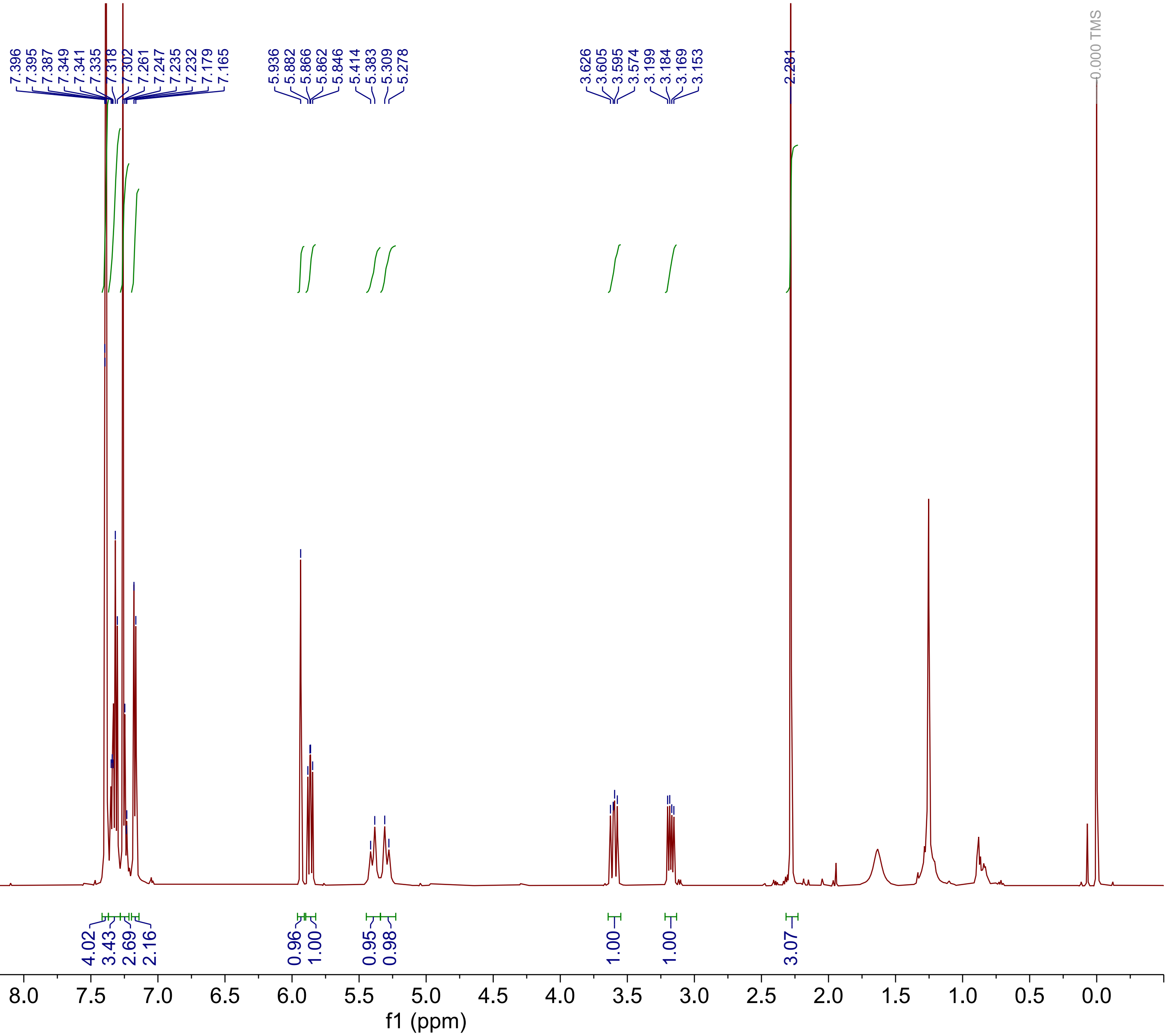

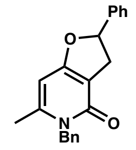

7

$^{13}C\{^{1}H\}$ NMR
126 MHz, $CDCl_3$

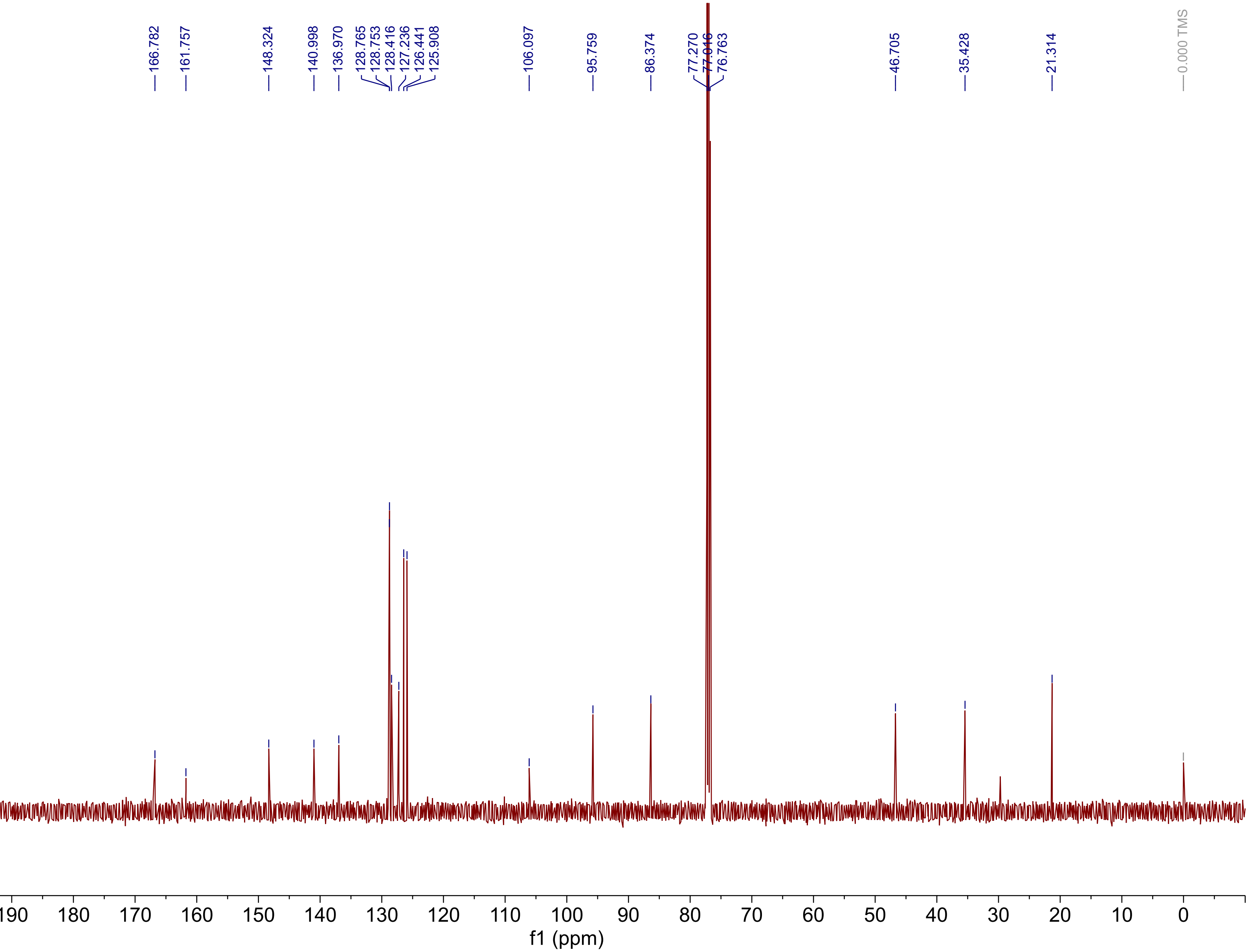

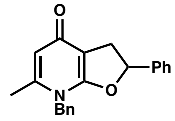

27

^{1}H NMR
500 MHz, $CDCl_3$

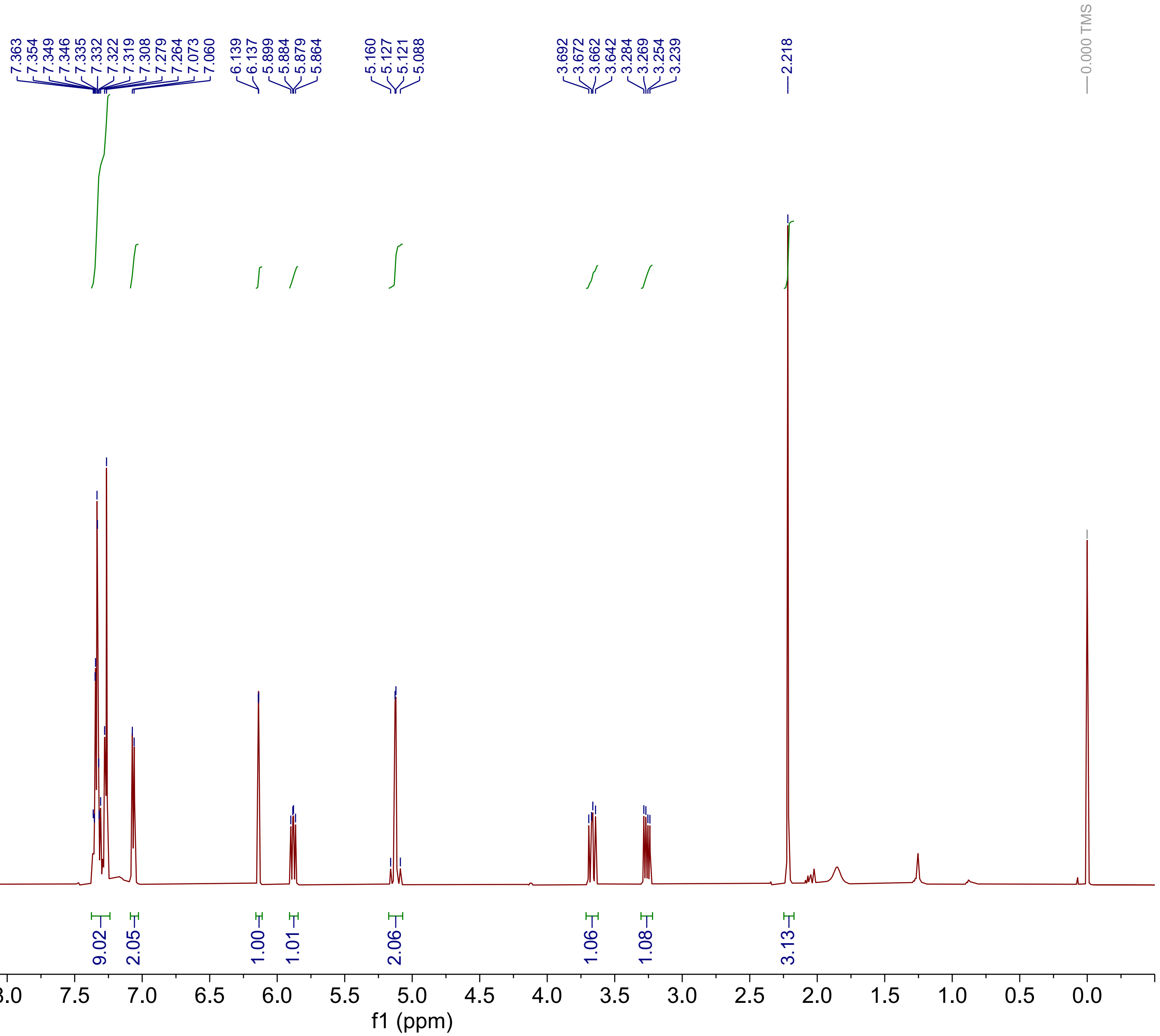

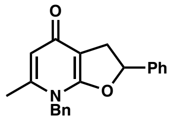

27

$^{13}C\{^{1}H\}$ NMR
126 MHz, $CDCl_3$

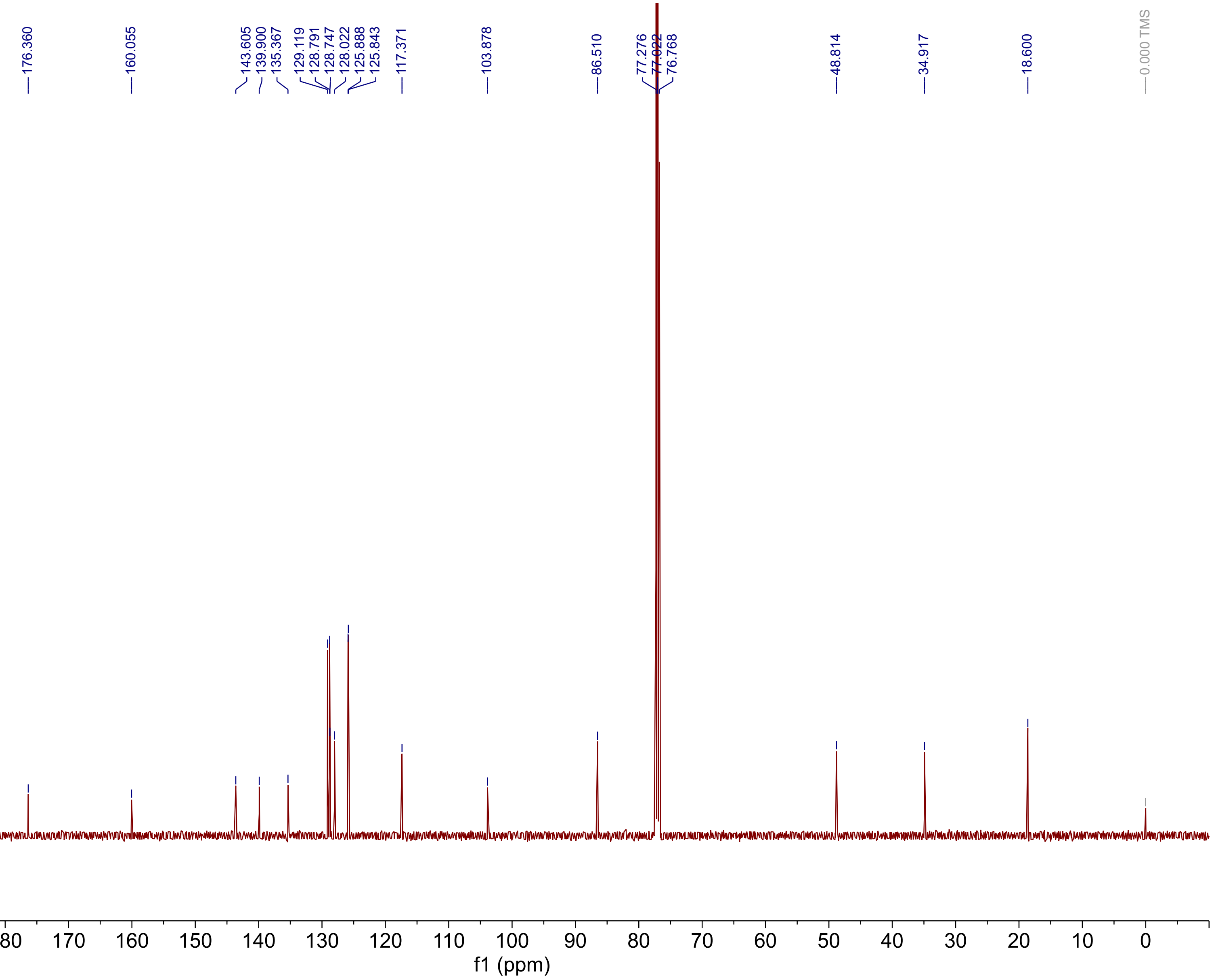

Supplement: Supplementary file 1 [file jo5c01187_si_001.pdf]
